# Predictive crystallography at scale: mapping, validating, and learning from 1000 crystal energy landscapes†‡

**DOI:** 10.1039/d4fd00105b

**Published:** 2024-06-03

**Authors:** Christopher R. Taylor, Patrick W. V. Butler, Graeme M. Day

**Affiliations:** a School of Chemistry, University of Southampton Southampton SO17 1BJ UK g.m.day@soton.ac.uk

## Abstract

Computational crystal structure prediction (CSP) is an increasingly powerful technique in materials discovery, due to its ability to reveal trends and permit insight across the possibility space of crystal structures of a candidate molecule, beyond simply the observed structure(s). In this work, we demonstrate the reliability and scalability of CSP methods for small, rigid organic molecules by performing in-depth CSP investigations for over 1000 such compounds, the largest survey of its kind to-date. We show that this highly-efficient force-field-based CSP approach is superbly predictive, locating 99.4% of observed experimental structures, and ranking a large majority of these (74%) as among the most stable possible structures (to within uncertainty due to thermal effects). We present two examples of insights such large predicted datasets can permit, examining the space group preferences of organic molecular crystals and rationalising empirical rules concerning the spontaneous resolution of chiral molecules. Finally, we exploit this large and diverse dataset for developing transferable machine-learned energy potentials for the organic solid state, training a neural network lattice energy correction to force field energies that offers substantial improvements to the already impressive energy rankings, and a MACE equivariant message-passing neural network for crystal structure re-optimisation. We conclude that the excellent performance and reliability of the CSP workflow enables the creation of very large datasets of broad utility and explanatory power in materials design.

## Abstract 2

We demonstrate the reliability and scalability of computational crystal structure prediction (CSP) methods for small, rigid organic molecules by performing in-depth CSP investigations for over 1000 such compounds.

## Graphical abstract

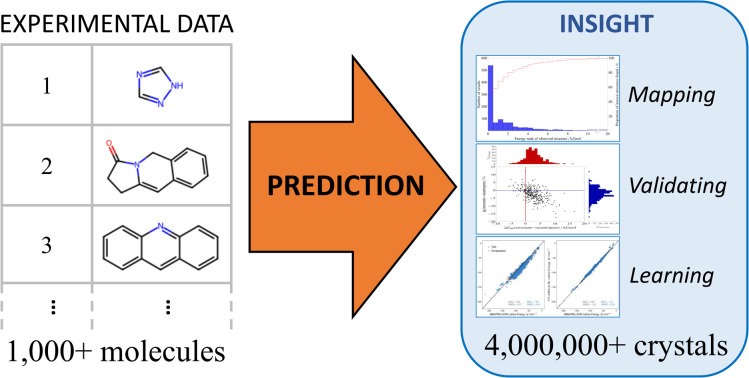

## 1 Introduction

The discovery of new materials is important for addressing many critical societal needs, including energy production and storage, pollution remediation and healthcare. Research endeavours aimed at improving the success and efficiency of functional materials discovery, based on traditional efforts developing our understanding of the rules of crystal packing and, more recently, applications of machine learning, have benefited greatly from the availability of databases of stable crystal structures.

A major resource for such efforts has been the growth of accessible, curated databases of crystal structures and (some of) their properties. The most general and widely-used include the Cambridge Structural Database (CSD)^1^ for organic and organometallic systems, and the Inorganic Crystal Structure Database.^2^ These resources are unparalleled in their volume of experimental crystal structure information, but do not currently offer information about calculated or hypothetical structures. Historically, such data was limited to specialised areas (such as the Atlas of Prospective Zeolite Structures^3^), though recent developments have made remarkable progress in generalising this concept, including extensions to the Crystallography Open Database (COD)^4–7^ and the Materials Project.^8^ In the field of organic molecular crystals, however, much of our understanding of the rules of crystal packing derive from databases of experimentally observed structures.

Modern computational chemistry, employing both molecular and solid-state simulation techniques, can add significantly to the information that is available from experimentally determined crystal structures, and identify previously unverifiable trends. Computational studies of polymorphism^9–13^ have evaluated the typical lattice energy differences between crystalline polymorphs of organic molecules, studies of conformations of flexible molecules^11,14^ in their observed crystal structures have improved understanding of the limits of molecular strain in stable structures, and studies of the thermodynamics of co-crystallisation^15–18^ have aided in rationalising a complex phenomenon with ramifications for experimental design.

A more complete and, crucially, predictive view of organic crystal packing can be obtained from crystal structure prediction (CSP).^19,20^ A core concept in CSP is the crystal energy landscape (or CSP landscape) – the set of plausible crystal packings for a chemical species (or combination thereof), representing an exploration of the crystalline configuration space to identify candidate structures predicted to be stable (which ideally includes any observed structures), ranked in terms of their energetic stability. These CSP landscapes are of great utility in understanding and rationalising the thermodynamic and kinetic behaviour of crystal systems; multiple low-energy minima that are close-lying on a CSP landscape may be indicative of a significant risk of polymorphism, while dynamical simulations exploring these landscapes give insight into the kinetic trapping of metastable forms^21^ and the observed absence of other predicted forms.^22,23^

Moreover, the energy landscape forms the foundation of the energy–structure–function maps that have in recent years demonstrated great power in materials discovery.^24–27^ By associating computed properties (*e.g.* gas uptake, charge carrier mobility) with hypothetical crystal structures from the CSP landscape, it becomes possible to predict whether a molecule is a promising candidate for creating new functional materials, *i.e.* if it has one (or more) favourably-ranked crystal structures which are predicted to achieve the desired property.

The techniques and challenges in the field of organic CSP have been reviewed;^19,20^ we provide a brief overview for the sake of context. CSP is typically considered a combination of two broad challenges: efficient and thorough sampling of the configuration space of crystal packing, and accurate, cost-effective structural optimisation, ranking, and property calculation.

The sampling of hypothetical crystal packing arrangements is made extremely difficult due to the “curse of dimensionality”—the number of independent degrees of freedom to sample creates a vast configurational space. As a result, simple grid-based sampling approaches must be eschewed in favour of more sophisticated techniques, such as low-discrepancy quasi-random sampling^28,29^ and genetic algorithm approaches.^30^

Meanwhile, the optimisation and ranking methods must be accurate enough to describe the fine balance of different intermolecular interactions (electrostatics, dispersion, hydrogen-bonding, *etc.*), resolving lattice energy differences often smaller than a kJ mol^−1^, while sufficiently cost-effective to be applied to very large numbers (≫10^5^) of trial crystal structures. Historically, this has entailed the use of simple empirical force fields, but modern developments often employ tailor-made force fields^18,31^ or machine-learned potentials derived from *ab initio* calculations. Still, empirical force fields retain their power even today due to their efficiency and broad transferability, often being the initial step of a hierarchy of increasingly accurate (and expensive) energy models employed in one CSP workflow.

Despite these two broad and ongoing challenges, organic CSP has demonstrated enormous success in diverse applications, crucially proving itself to be a truly predictive technique, guiding synthesis and discovery of novel forms of porous materials,^24^ highly-flexible pharmaceutical molecules,^31^ co-crystals,^32^ simple molecules previously thought to be monomorphic^33^ and templating of predicted metastable polymorphs.^34^ A recurrent landmark in the field is the series of Blind Tests of CSP, showcasing the diversity of methods (and success rates thereof) employed within different CSP techniques to predict experimental crystal structures without any knowledge beyond the molecular chemical diagram.^35^ Recent iterations of the Blind Test have demonstrated that CSP method development is successfully keeping apace with the complexity of molecules and crystal structures specifically selected to stress-test it.

Some of the most consequential recent developments in CSP have employed machine learning (ML) techniques in one form or another.^36^ Among the most intuitive applications is the use of ML to learn relationships between the structure and lattice energy, either by learning the difference (*i.e.* Δ-ML) between lattice energies computed at a lower level of theory to those at a higher level,^37–40^ or to learn the relationship between the lattice energy and the ML descriptors directly through the training of ML potentials.^41,42^ These approaches have achieved high accuracy predictions at a fraction of the cost of the full periodic density functional theory (DFT) reference calculations – particularly significant given the latter's ubiquity in recent Blind Test entries.

That said, ML techniques have further applications in CSP beyond improved optimisations and energy rankings. In particular, ML and related approaches applied to databases of experimental crystal structures and properties have demonstrated success in predicting NMR chemical shifts^43^ and in formulating models of molecular hydrogen bond propensities within crystals.^44^ Recent work by Cersonsky^45^ has demonstrated machine-learning of the relationship between crystal lattice energies and the relative contributions to these by different chemical functional groups, paving the way for data-driven insights into new crystal engineering techniques. ML has also been shown to have potential to enhance the analysis of crystal energy landscapes, by identifying structure–function relationships that might evade simple inspection but nevertheless offer explanatory and predictive power.^46^

Our aims in this work are threefold. Firstly, we seek to demonstrate the capability of our method for efficient, large-scale rigid-molecule organic CSP by presenting the results of the largest to-date CSP study, applying the methods to over 1000 molecules with observed crystal structures in the CSD. Secondly, we in turn assess the quality and reliability of our method by evaluating how often the experimental crystal structures of these molecules are reproduced in our CSPs, and how well they are ranked energetically compared to hypothetical structures on their CSP landscapes. Finally, we demonstrate example applications of this dataset, including assessing the distribution of CSP-derived space group preferences as a function of predicted lattice energy, spontaneous resolution of chiral molecules and the training of transferable machine-learned energetic models from a very large set of CSP landscapes.

## 2 Computational methods

### 2.1 Molecule selection

We used the CSD's ConQuest software and Python API ^1^ to search the CSD for crystal structures of rigid molecules to which our CSP methods could be applied on a very large scale. Restricting our search to solved crystal structures (*i.e.* with coordinates for all atoms in the asymmetric unit) of single chemical species (no co-crystals, solvates, inclusion compounds), we additionally filtered structures based on the following criteria: containing only elements from C, H, N, O, F; *Z*′ ≤ 1; molecular weight less than 230; and importantly containing no rotatable bonds (as defined by the CSD's internal criteria).

### 2.2 Molecular geometry optimisation

For each molecule, we began by extracting its in-crystal conformation from the corresponding CSD entry (where there is more than one entry, we select only the first listed in the database). This molecular conformation was optimised in DFT (as implemented in Gaussian^47^), using the B3LYP^48,49^ exchange–correlation functional, a Pople-type 6-311G** basis,^50^ and Grimme's D3 dispersion correction^51^ with Becke–Johnson damping.^52^

Distributed multipole analysis (DMA,^53,54^ using the GDMA package) was performed on the resulting molecular conformers' charge densities to obtain atom-centred multipoles, as part of the model potential applied during lattice energy minimisation of trial crystal structures; multipoles up to hexadecapole were calculated for all atoms. The MULFIT^55,56^ software was used to fit atomic point charges for each molecule to best describe the molecular electrostatic potential. The resulting molecular conformers and their sets of multipoles are then used as the inputs to CSP, with each unique molecule represented by a single conformer and corresponding set of atomic multipoles and charges.

### 2.3 Crystal structure generation and optimisation

CSP was performed using the Global Lattice Energy Explorer (GLEE) package,^28^ which uses quasi-random sampling of crystal packing variables to generate trial crystal structures uniformly distributed across the lattice energy landscape, followed by rigid-molecule lattice energy minimisation using an anisotropic atom–atom intermolecular force field. All resulting local energy minima are treated as possible crystal structures of the molecule.

Space group symmetry is used to reduce the dimensionality of the search space, so that only the position and orientation of molecules in the asymmetric unit are sampled, with all other molecules in the unit cell generated by symmetry. In this study, we restrict ourselves to generating crystal structures with one independent molecule in the asymmetric unit (*Z*′ = 1). We sampled the 26 most commonly observed space groups for organic molecular crystals (listed in ESI†); these space groups cover over 99.4% of *Z*′ ≤ 1 structures in the CSD. These space groups were sampled equally, irrespective of their observed frequency in the CSD: quasi-random structures are generated and lattice energy minimised until 10 000 successfully energy minimised crystal structures were found in each space group (260 000 structures per molecule). The CSP process is highly parallelisable, as each crystal structure is independent.

The trial crystal that passed geometric checks were lattice energy-minimised in three stages. Non-electrostatic interactions (principally intermolecular dispersion and exchange-repulsion) were described by the FIT *exp* − 6 force field,^57,58^ supplemented by fluorine parameters from Williams and Houpt.^59^ At the final stage of optimisation, performed using DMACRYS,^58^ the FIT potential was applied with atomic multipole electrostatics. Full details are provided in ESI.†

It is commonplace that multiple unique initial configurations optimise to the same local energy minimum. We remove these duplicates by fast comparison of simulated powder X-ray diffraction patterns, followed by structural comparisons using the COMPACK^60^ algorithm as implemented in the CSD Python API.

#### 2.3.1 Locating experimental structures on the landscapes

To assess our CSP workflow's performance, comparison of the known experimental crystal structures to the sets of predictions was automated using the COMPACK algorithm between the experimental crystal structures of these molecules and every unique crystal structure of that molecule in the CSP set.

### 2.4 Machine learned interatomic potentials

To investigate the potential of the CSP dataset to train data-derived models, a subset of the predicted crystal structures with energies within 8.0 kJ mol^−1^ of the global energy minimum on their CSP landscape, were selected for training a lattice energy correction to the FIT+DMA force field. The subset was determined by active learning *via* query-by-committee using a committee of 8 high-dimensional neural network potentials (NNPs), with selected structures evaluated by DFT+D single points at the B86bPBE+XDM level. From this, a dataset of the crystal structures and the corresponding energy correction between the FIT+DMA and B86bPBE+XDM lattice energies (Δ*E*) was created. B86bPBE+XDM lattice energies were calculated as the total energy less the energies of the isolated molecules from the unit cell calculated with the same basis set and tolerances.

The initial dataset (before the active learning iterations) was generated by randomly selecting up to 10 low energy predicted crystal structures for each compound, resulting in a dataset of 10 249 structures approximately evenly distributed across the compounds. To evaluate transferability, the total dataset was partitioned into a training dataset consisting of the CSP structures for a randomly selected *ca.* 85% of the compounds and an extrapolation test set consisting of CSP structures for the remaining compounds. A further in-domain test set was formed by randomly extracting one structure per compound from the training set. The NNPs were then trained on the remaining training dataset to yield a Δ-ML model capable of predicting the lattice energy correction. The standard deviation between the ensemble of NNPs was used to estimate the uncertainty of predictions, and was exploited in the active learning iterations to add high uncertainty candidates from the remaining low energy predicted structures of the training compounds, which overall added a further 1000 structures to the training set. Corrected CSP landscapes were calculated using the final model by adding predicted lattice energy corrections to the FIT+DMA energies (FIT+DMA+Δ-ML). Additionally, for performing unconstrained geometry optimisations MACE equivariant message-passing neural network (MPNN) models were trained using a dataset derived from that of the NNP correction model. Further details of the datasets and machine learning models are provided in the ESI.†

## 3 Results

### 3.1 Diversity of survey set

**Fig. 1:**
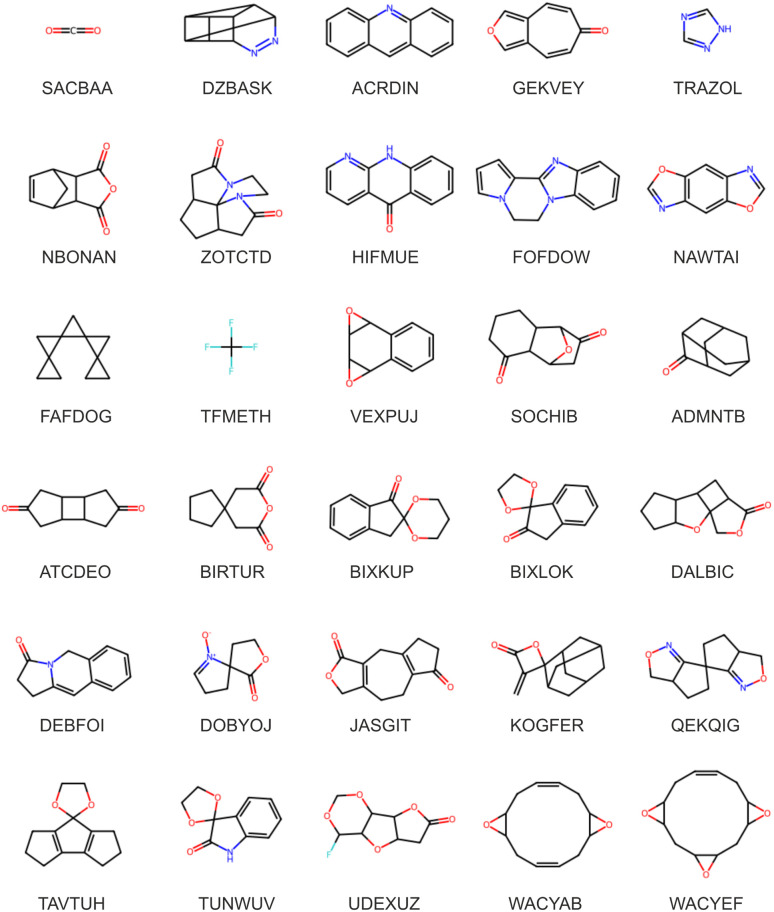
Molecular diagrams and crystal structure CSD reference codes for (top three rows) a random selection of the 1007 molecules included in the large-scale CSP study. The bottom three rows show molecules in the set with the largest differences between in-crystal and optimised molecular geometries (as measured by all-atom RMSD); the CSP landscapes for these molecules were re-optimised using the transferrable MACE model (final section).

Our aforementioned search criteria yielded 1007 distinct molecules crystallising in 1040 crystal structures observed in the CSD. The constraint of no rotatable bonds necessarily limits chemical diversity, but despite this, our candidate set still displays a variety of chemical functionalities and molecular structures, as seen in Fig. 1 and Table 1. A complete list of molecules, including formulae, CSD refcode identifiers, SMILES strings, and systematic and common names is available in the ESI.†

**Table 1:** Total counts of selected functional groups across the full set of molecules, as assessed by RDKit ^61^ from SMILES strings

Functional group	Count	Functional group	Count	Functional group	Count
Benzene	418	Ether	572	Ester	134
Pyridine	76	Amide	297	Carbonyl	702
Imidazole	35	Imide	43	Imine	13
Furan	22	Lactone	91	Secondary amine	405
Piperdine	51	Epoxide	73	Tertiary amine	537
Urea	22	Ketone	269	Halogen (F)	240

#### 3.1.1 Deviation between experimental and gas-phase conformations

Despite restricting our set to molecules containing no rotatable bonds, this does not preclude molecular flexibility entirely. More complex collective motions cannot be described in terms of a single torsional angle about a covalent bond, and so molecules displaying such conformational flexibility are present in this candidate set – the prototypical example is a ring “flip” or buckling, such as the boat-chair interconversion of cyclohexane rings.

**Fig. 2:**
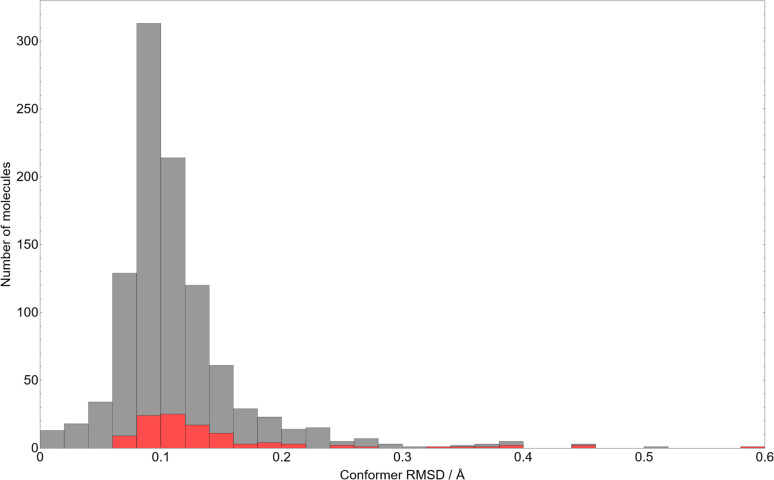
A histogram of the RMSD between all atomic positions in the gas-phase optimised CSP candidate molecules, relative to their initial in-crystal conformations. The red portions of each bar indicate the molecules in that bin with spiro carbon centres.

As a measure of the typical deviation in molecular geometry between the observed crystal structures and the DFT optimised molecules used in CSP, we present in Fig. 2 the histogram of all-atom root-mean-squared deviations (RMSD) in atomic positions between the gas-phase conformers used in CSP and their in-crystal initial conformations as extracted from the CSD.

As might be expected from such rigid molecules, the average RMSD after gas-phase optimisation is very small – approximately 0.11 Å, which corresponds to *e.g.* adjusting the C–H bond lengths in fluorobenzene by 0.16 Å. While the distribution is skewed towards small conformational changes, the outliers with larger RMSD values demonstrate the limitations of defining molecular flexibility in terms of rotatable bonds alone. The largest RMSD values correspond to systems where changes in ring conformation cause large overall molecular changes – the largest observed RMSD of 0.60 Å occurs in 7-oxa-1-azaspiro(4.4)non-1-en-6-one 1-oxide (CSD reference code: DOBYOJ), in which a 5-membered saturated ring can twist about a spiro carbon centre. The highest molecular RMSD values are largely associated with molecules containing spiro carbon atoms (denoted by the red portion of the bars in Fig. 2).

Regardless, the overall inflexibility displayed by this set serves as a strong indicator that our assumption of a single, near-crystalline conformer of each molecule is reasonable, and unlikely to be a systematic source of error in predicting many known structures of molecules in this set.

#### 3.1.2 Crystalline diversity

**Fig. 3:**
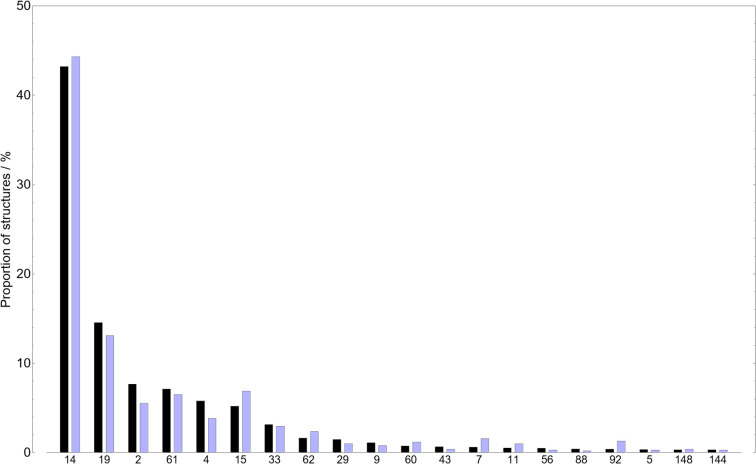
The relative frequency of space groups observed for crystal structures (where *Z*′ ≤ 2) of molecules in the CSD with a molecular weight under 230 (black) and the subset (blue) of these molecules with no rotatable bonds selected for our CSP surveys. The distributions are presented for only the 20 most common space groups (of the general molecule case) for clarity.

Fig. 3 shows a comparison of the distribution of crystallographic space groups for most small molecules in the CSD to the rigid-molecule subset selected for our CSP survey. There is no significant difference in the relative occurrence of different crystalline symmetries between the two sets, though the relative ordering of space groups by frequency varies slightly. For example, space group 15 (*C*2/*c*) is observed slightly more frequently in our rigid set and space group 2 (*P*1̄) slightly less so. Regardless, our rigid molecule set is reasonably representative of the range of crystal packing symmetries observed in the CSD.

In contrast, our rigid molecule set displays a diminished frequency of hydrogen bonding (H-bonding) in the observed crystal structures compared to the CSD more generally, as might be expected by excluding rotatable bonds. 26.7% (277) of crystal structures of molecules in our subset contain at least one intermolecular H-bond. This compares to 62.3% of structures of molecules of similar size without rotatable bond restrictions, demonstrating the bias introduced by the omission of common H-bonding groups due to their flexibility, including alcohols (–OH), carboxylic acids (–COOH), and primary amines (–NH_2_). However, the proportion of H-bonded systems is still significant enough that meaningful H-bond chemistry is incorporated in our survey, albeit underrepresented. Consequently, our set in turn over-represents chemistry such as π–π stacking and “weak” (*i.e.* more isotropic, less localised) interactions, and assessments of our CSP energy model's performance must be made with these biases in mind.

### 3.2 Quality assessment of the CSP results

We propose that the dataset of predicted crystal structure landscapes across a large, diverse set of molecules is valuable for the development of future predictive models. To evaluate the quality of the dataset, we assess three aspects: completeness of the landscapes; how well, geometrically, the CSP calculations reproduce the known crystal structures of these molecules; and the quality of the relative energies of the predicted structures.

The 26 space groups included in our standard search include those with an observed frequency above 0.05% in the CSD. Naturally, in a very large survey of molecules, we include some that crystallise in less frequent space groups; for these 8 cases, we added the space group of the observed crystal structure to the search. In 3 additional cases, the symmetry of the observed structure means that it could not have been sampled without performing CSP with multiple independent molecules (*Z*′ > 1); in these cases, we include the datasets in our study, knowing that the observed crystal structures could not have been located. Of those that could have been located, the searches find matches for 1034 of the 1040 observed crystal structures: reasons for the 6 missed matches are discussed below.

**Fig. 4:**
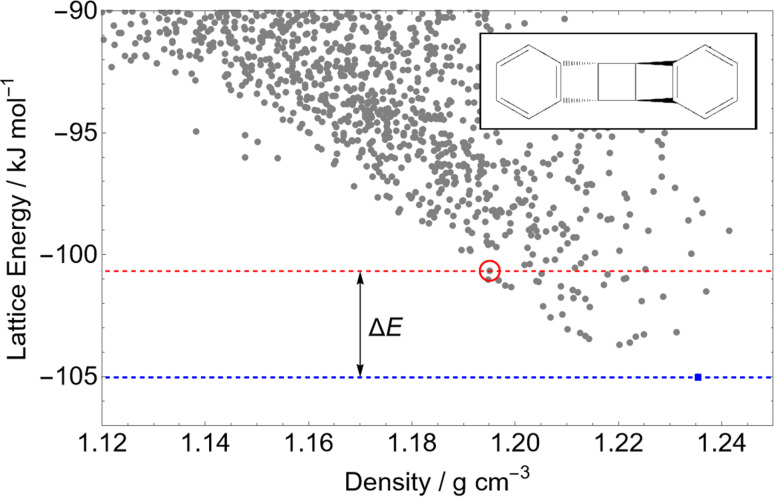
An example CSP landscape for a single molecule 3,4.7,8-dibenzo-tricyclo(4.2.0.0^2,5^)octa-3,7-diene – the set of predicted lattice energy minima from our CSP workflow. Each point represents a local energy minimum, and thus stable hypothetical crystal packing. The blue square point is the global energy minimum, which in the absence of experimental information is taken to be the most likely crystal structure. If the red circled point is a match to the experimentally-observed crystal structure, Δ*E* is the difference in energy between it and the global minimum, the energy rank used in this work as a measure of the quality of the calculated energies.

**Fig. 5:**
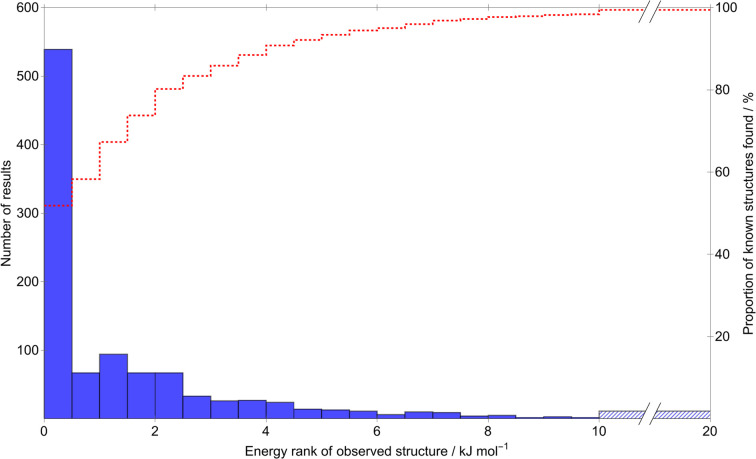
A histogram of the frequency (blue bars) with which our CSP workflow achieves a match to the experimentally-known structures of our molecule set, grouped by the relative energy of that match compared to the CSP global minimum (0.5 kJ mol^−1^ bins). The red dashed line relates to the secondary (right) *y*-axis, the proportion of known structures located successfully as a function of the relative energy at that bin and below. Note the broken *x*-axis; the highest-energy bin (blue hatching) encompasses all matches with relative energy greater than 10 kJ mol^−1^.

A main assumption in CSP is that the observed crystal structures of a molecule correspond to the lowest energy possible structures. We use this assumption to assess the quality of the calculated energies. Fig. 5 summarises the energetic ranking of experimentally observed crystal structures within the CSP landscapes *via* the distribution of Δ*E*, the energy difference between the prediction matching the experimental crystal structure and the global lattice energy minimum crystal structure on their parent molecule's CSP landscape; an example landscape is shown in Fig. 4. In the case of molecules with chiral centers and only one stereoisomer present, Δ*E* is calculated only among Sohncke space groups (those whose symmetry elements contain only translations, rotations and roto translations).

Of the 1034 experimentally determined crystal structures where a matching structure was identified on the CSP landscape, 424 (41%) correspond to the global lattice energy minimum from CSP. Those that do not correspond to global energy minima could be due to inaccuracy of the model potential (FIT+DMA), neglect of other contributions to the lattice free energy or where the kinetics of crystallisation favour a metastable structure. Nyman and Day found that, for crystals of rigid molecules, lattice vibrational contributions to room temperature free energy differences between polymorphs rarely exceed 2 kJ mol^−1^;^9,10^ 767 (74%) of the observed crystal structures are found within 2 kJ mol^−1^ of the global energy minimum – the estimated error introduced by neglecting lattice vibrations and thermal expansion in the CSP calculations.

Furthermore, the known structure(s) almost always lie within 8 kJ mol^−1^ of the global lattice energy minimum (1011, 97.8%, of the observed crystal structures), consistent with the observation^10^ that known polymorphic pairs of small, rigid molecules are rarely separated by more than this energy difference. Thus, the energy model used here, combining empirically parameterised repulsion–dispersion with atomic multipole electrostatics, provides energy ranking of crystal structures that is consistent with observed polymorphism, within the limits of temperature-free lattice energy-based predictions.

**Fig. 6:**
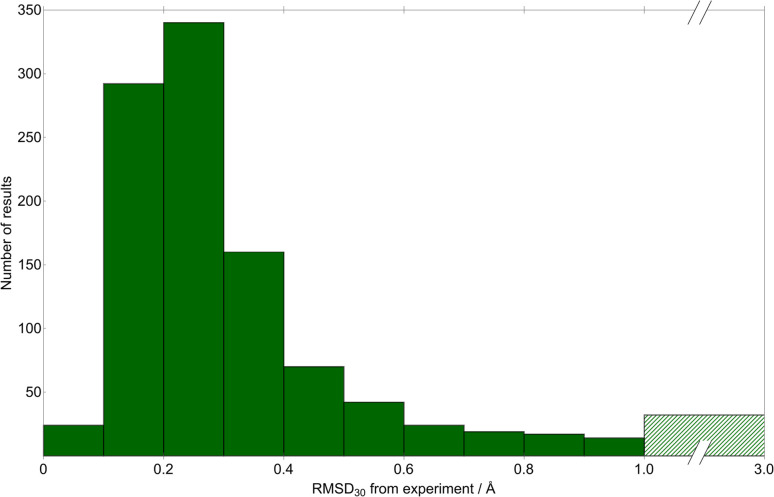
A histogram of the geometric deviation between experimentally-determined crystal structures and the corresponding matching structures from CSP. The deviation is measured as the RMSD in atomic positions within 30-molecule clusters from experimental and CSP structures. Note the broken *x*-axis – the largest-deviation bin (green hatching) includes all matches with RMSD_30_ greater than 1.0 Å.

**Fig. 7:**
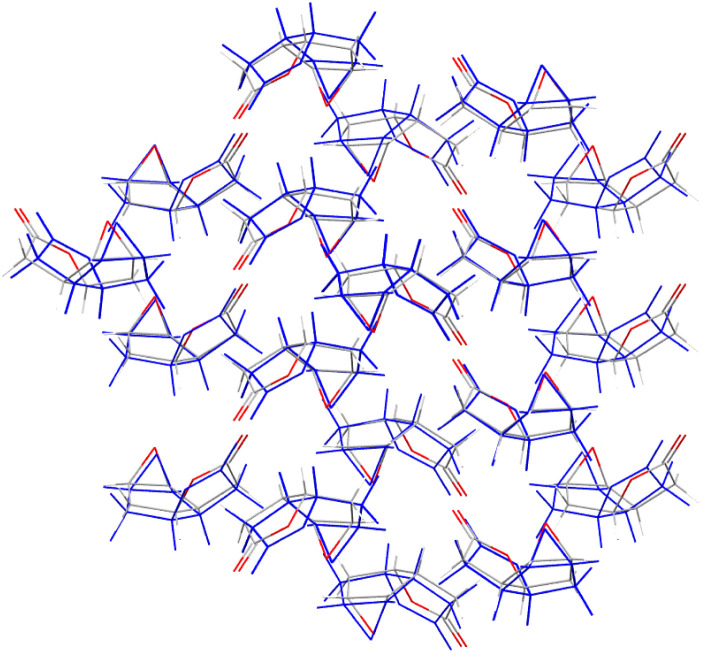
Overlay of 30-molecule clusters from the X-ray determined crystal structure (atoms coloured by element, CSD reference code SIBJIX) with the matching prediction (blue) – the global energy minimum structure for (1a*R*,2a*S*,5a*S*,5b*S*)-perhydro-4*H*-oxireno(3,4)cyclopenta(1,2-*b*)furan-4-one. For this match, RMSD_30_ = 0.393 Å.

We quantify the geometric quality of the predictions using an all-atom RMSD within 30-molecule clusters (RMSD_30_) from experimentally-determined crystal structures and their corresponding match within the CSP sets. A histogram of RMSD_30_ (Fig. 6) shows that geometric agreement is generally very good: RMSD_30_ is below 0.4 Å in 78.9% (816) of matches. As a visualisation of this level of agreement, Fig. 7 shows an overlay of the X-ray determined crystal structure of (1a*R*,2a*S*,5a*S*,5b*S*)-perhydro-4*H*-oxireno(3,4)cyclopenta(1,2-*b*)furan-4-one ^62^ (CSD reference code SIBJIX) and the predicted global lattice energy minimum, with RMSD_30_ = 0.393 Å. As a reference for these values, consider the RMSD_30_ between structural determinations of the same crystal structure at ambient and low temperature: RMSD_30_ = 0.204 Å between neutron diffraction crystal structures of naphthalene at 5 K and 295 K, and RMSD_30_ = 0.160 Å between 20 K and 330 K crystal structures of form I paracetamol.^63^ 327 matches have RMSD_30_ below 0.204 Å, *i.e.* have geometric deviations that are of a magnitude that can be explained by the temperature-free nature of structural optimisations used in CSP. While known crystal structures are reproduced very well by CSP in most cases, there are a small number where agreement is less satisfactory: in 32 cases (3% of structures), RMSD_30_ > 1 Å. Despite what might be assumed, we find no significant correlation between the RMSD_30_ of the experimental match and the molecular RMSD of the parent gas-phase conformer used for CSP. Assuming an experimental match is found, the geometric quality of the match is only weakly sensitive to the difference in the molecular conformation used for CSP; even the most extreme outlier in molecular conformational change (the aforementioned DOBYOJ) achieves a reasonable geometric match to the experimental crystal structure, with RMSD_30_ = 0.654 Å.

It is evident that an overwhelming proportion of such rigid molecules can successfully be treated with the CSP workflow implemented here. Our sampling procedure followed by a cost-effective, approximate minimisation method successfully locates the vast majority of observed crystal structures with excellent geometric agreement with the experimental structure, and routinely ranks them as among the most stable structures on the landscape.

#### 3.2.1 Analysis of individual outliers

The cases where no match whatsoever is found to the experimental structure are limited – approximately 0.5% of the experimental structures considered.

One missed match was for the molecule 4,8*b*-dihydropyrrolo[3,4-*b*]indole-1,3(2*H*,3a*H*)-dione (CSD reference code BUVGAC), which displays a large deviation in molecular geometry between the observed crystal and the gas phase optimised geometry used in CSP. Flexibility in the fused ring system allows the molecule to “fold up” in the gas-phase optimisation, sufficient to change its packing behaviour. As a result, the experimental crystal structure is not a minimum on a CSP landscape derived from the gas-phase conformer.

In the case of DNNEPH10 (1,8-dinitroso-naphthalene), we fail to find the experimental structure despite an apparently rigid, planar molecule that changes very little in the gas-phase optimisation. However, even optimising the known crystal structure using our FIT+DMA energy model results in a final structure that does not match, *i.e.* the experimental structure appears to be unstable at our level of theory. This may indicate a failure of our energy model for a case of somewhat unusual chemistry.

Two of the missed matches are unusual cases in which a refcode “stem” (the initial six letters, typically shared by a “family” of multiple CSD entries of the same species) is used by crystal structures containing distinct chemical bonding arrangements. Our procedure takes a single representative of a CSD refcode family as the source for the molecular connectivity, which was assumed to stay fixed. In the case of refcodes XUGHUD/XUHGUD01, there is a tautomeric difference, which led to no match with XUHGUD01. For IHEPUG/IHEPG02, the subject molecule is a diastereomeric fused ring system, which exists in an *anti* configuration in IHEPUG, but a *syn* configuration in IHEPUG02. It is arguable that these crystal structures should not be considered part of the same “family”, as these distinct bonding arrangements are not interconvertible. CSP was performed with the isomer found in IHEPUG, so no crystal structure matching IHEPUG02 was located. These two systems demonstrate a shortcoming of our approach, in that we assumed that a given CSD refcode “stem” always denotes the same molecular connectivity, including protonation states. Fortunately, these are the only instances of this assumption failing in the entire set.

Our final missed match occurs for QIBCEK, benzo(*f*)phthalazin-4(3*H*)-one, another rigid planar molecule. However, upon inspection, we posit that there are flaws in the experimental determination of this structure as held in the CSD – there are extremely close hydrogen contacts (<1.4 Å) and an unsatisfied potential hydrogen-bonding arrangement despite 1 : 1 donor–acceptor availability. While we retain it as a missed match for the purposes of conservatively assessing our CSP method's performance, we also emphasise that such a large-scale, unbiased workflow has potentially identified an incorrect experimental structure simply through its absence from the CSP landscape.

### 3.3 Revisiting empirical rules

Large databases of experimentally determined crystal structures have been analysed to uncover trends in the packing preferences of organic molecules. The availability of high quality crystal energy landscapes should allow organic solid state researchers to gain deeper insight into these preferences and, we hope, to discover new rules that will benefit the field of crystal engineering. We give two examples here: the unequal frequency with which molecules occupy the possible space groups and the spontaneous resolution of chiral molecules.

#### 3.3.1 Space group preferences

There are strong space group preferences for experimentally observed crystal structures: over 80% of molecular crystals occupy 5 of the 230 three-dimensional space groups. Having applied an approach in generating trial crystal structures that is unbiased in how the 26 space groups included in our searches are sampled, we analyse the results of CSP to investigate space group preferences within the low energy structures on the set of crystal energy landscapes.

**Fig. 8:**
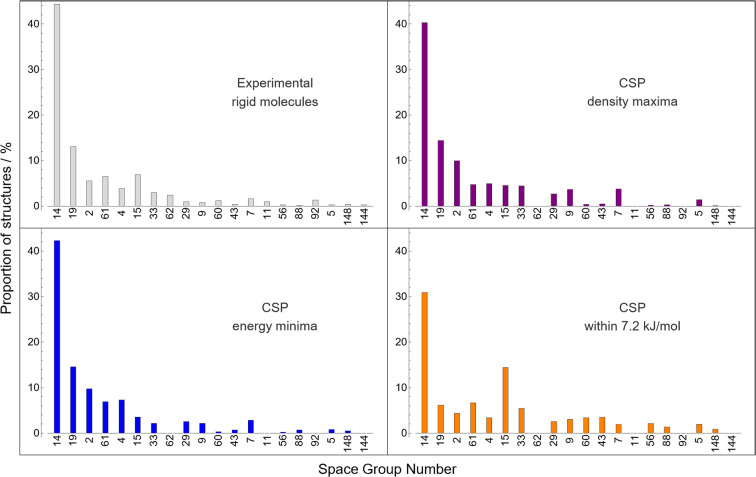
The relative frequencies of space groups of crystal structures. Grey (top left) are those of rigid molecules in the CSD (as in Fig. 3), while the rest are obtained from CSP landscapes at the global density maximum (purple, top right), at the global energy minimum (blue, bottom left), and within 7.2 kJ mol^−1^ of the global energy minimum (orange, bottom right). The ordering of space groups on the *x*-axis is chosen to match that in Fig. 3. Only the 20 most common space groups for rigid molecules are presented for clarity.

We preface our analysis by emphasising that the space group frequencies presented in Fig. 8 are those for the CSP landscapes, *i.e.* only those of structures generated using an asymmetric unit containing a complete molecule (in these single-species systems, *Z*′ = 1) and without detecting and assigning additional symmetry after the minimisation. In contrast, those of the observed crystal structures in the CSD are the full, maximal-symmetry space group (allowing fractions of molecules in the asymmetric unit, *i.e. Z*′ ≤ 1). Hence the space groups enumerated for the CSP set can be thought of as “lower bounds” on the symmetry – higher-symmetry space groups may be assignable if the molecules present internal symmetry.

The global lattice energy minimum for each molecule is the energetically preferred packing; the distribution of space groups among the global minima structures are highly consistent with the observed statistics – perhaps as expected, as CSP has been demonstrated in this work to often identify observed structures of these molecules as global minima.

Space group frequencies are often explained by close packing arguments: the commonly observed space groups have combinations of symmetry elements that facilitate close packing of irregular shapes.^64^ Examining the space group distribution amongst the densest predicted crystal structure for each molecule gives a similar distribution to that from global energy minima; minimising energy and maximising density lead to similar space group preferences. However, there are differences after the three most popular space groups: the next few have almost equal frequencies among high density structures, suggesting that they are equally good at promoting close packing and that observed differences between these space groups relate to subtler influences of symmetry on lattice energy.

Considering hypothetical structures higher on the CSP energy landscape (up to the usual energy limit of polymorphism, 7.2 kJ mol^−1^, in the bottom right panel of Fig. 8), we see a further flattening of the distribution, and an over-representation of space group 15 (*C*2/*c*) compared to global energy minima structures. These changes in distribution with energy, which we do not examine in deeper detail here, are only available from access to complete energy landscapes and are relevant in the discovery of high energy, metastable materials, which have sometimes been observed to have attractive properties.^24,65^ Thus, we feel that large datasets of CSP landscapes hold potential for generalising our understanding of symmetry preferences in molecular crystals.

#### 3.3.2 Spontaneous resolution of chiral molecules

As a second example of insight that can be gained from large numbers of crystal energy landscapes, we examine the tendency for spontaneous resolution of chiral molecules. It is generally accepted that crystallisation from a racemic solution of a chiral molecule more frequently yields racemic crystals rather than undergoing spontaneous resolution into a mixture of crystals, each containing a single stereoisomer.^66^ However, information in structural databases alone is limited: knowing whether crystals were grown from a racemic or enantiomerically pure solution is necessary to interpret the incidence rate of spontaneous resolution and the molecular characteristics that influence this behaviour. Furthermore, where enantiomers separate upon crystallisation, it is not possible to grow racemic crystals, so that comparison of racemic *vs.* enantiomerically pure crystal structures is not possible. Computed crystal energy landscapes make it possible to compare the structures and relative energies of the alternative crystallisation outcomes.

**Fig. 9:**
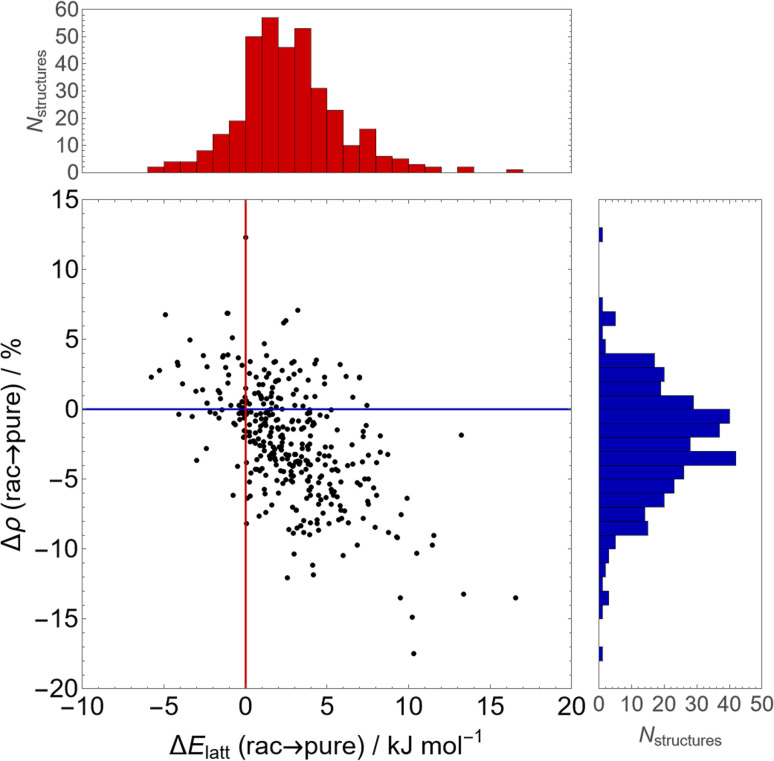
The difference in lattice energy (Δ*E*_latt_, top red histogram) and relative density (Δ*ρ*, right blue histogram) between the energy minimum in Sohncke (enantiopure) space groups and that in racemic space groups, for all molecules in our set containing at least one chiral centre. The scatter plot (center) displays the relationship between these values for each comparison (red and blue lines indicate the origin, *i.e.* no change in either quantity).

In Fig. 9, we show the difference in stability and density between the energy minimum across all Sohncke (*i.e.* enantiopure) space groups and the minimum across enantiogenic (*i.e.* racemic) space groups for the 356 molecules in our set containing at least one chiral centre. This energy difference represents the propensity for spontaneous resolution of a racemic solution compared to a racemic crystal of both enantiomers.

In general, there is a slight but consistent lattice energy penalty to enantiopurity – the average difference in lattice energy between the enantiopure global minimum and the racemic global minimum is 2.7 kJ mol^−1^, favouring the racemate. Of the 356 chiral molecules, racemic crystallisation is preferred in 86% (305) of molecules and spontaneous resolution is predicted to occur for approximately 14% (51) of molecules, although those with small lattice energy differences could be influenced by thermal contributions that are not included here. Moreover, this is accompanied (or perhaps driven) by improved packing in the racemate – on average, optimal enantiopure structures are 2.3% less dense than optimal racemic structures, consistent with the empirical Wallach's rule.^67,68^

### 3.4 Machine learned interatomic potentials

One of the more straightforward applications of machine learning in CSP is for the prediction of high quality lattice energies, to reduce the cost of geometry optimisations or of the final energy ranking of structures.^69^ Such approaches have been demonstrated in molecular organic CSP by training models on the landscapes of individual molecules.^37,39,40^ The CSP dataset developed in this work has significant potential for training data-derived models for organic crystals that could be applied more broadly.

#### 3.4.1 Transferable Δ-ML lattice energy corrections

To illustrate the use of CSP to train transferable machine-learned energy models, we trained a committee neural network potential using atom-centred symmetry functions^70,71^ for lattice energy corrections to the force field used in this work (FIT+DMA), correcting the lattice energies to the B86bPBE+XDM level. An initial model was trained on 7950 selected crystal structures from *ca.* 85% of the CSP landscapes (up to 9 crystal structures per landscape), randomly selected from within 8 kJ mol^−1^ of the global energy minimum of each landscape. This corresponds to just under 5% of the crystal structures (166 395) within this energy range for these landscapes. One crystal structure per landscape was withheld as an in-domain test set, while 10 crystal structures per landscape from the remaining CSP landscapes are used as an extrapolation test set. Following initial training, active learning was applied to identify crystal structures from the training landscapes with highest uncertainty in the lattice energy correction. After two iterations (adding 1000 training structures), a slight decrease in errors was observed in the test set, but no improvement in the extrapolation set (see ESI†), so training was halted. We also tried a third iteration of active learning with a wider energy window on each landscape, potentially including more diverse structures, but this did not lower the errors on the test or extrapolation sets. Consequently, we decided to proceed with the model after two iterations of active learning.

**Fig. 10:**
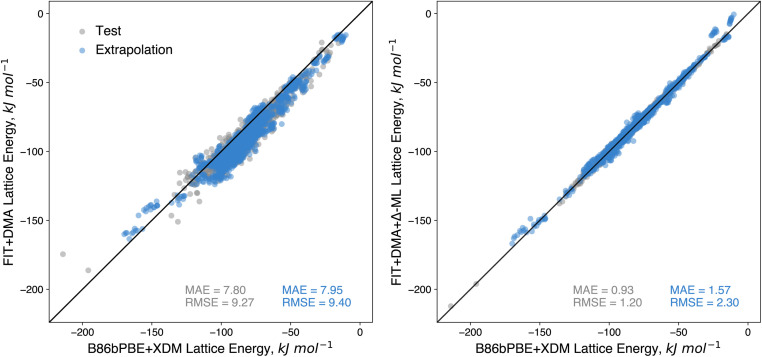
Correlation of (left) force field (FIT+DMA) lattice energies and (right) force field with machine learned correction *vs.* DFT (B86bPBE+XDM) lattice energies. The test set (grey data points) consists of unseen crystal structures from CSP landscapes of molecules that were seen during training. The extrapolation set consists of predicted crystal structures from molecules withheld from training.

The performance of the resulting model on the test set shows remarkably low error (Fig. 10), returning an MAE of just 0.93 kJ mol^−1^. Moreover, a similarly low MAE of 1.57 kJ mol^−1^ is achieved on the extrapolation test set, which contained crystal structures of compounds not included in the training of the correction. Compared to the errors for the baseline FIT+DMA, which returned MAEs of 7.80 and 7.95 kJ mol^−1^ on the test set and extrapolation set respectively, the correction offers a marked improvement in accuracy. The fact that the errors for the correction are slightly higher on the extrapolation set shows that there are limitations to the transferability of the correction. We expect that better transferability can be achieved as we increase the number of CSP landscapes available for training; the generation of additional landscapes can be targeted to weaknesses in the underlying force field and molecular types where the current machine learned correction has large errors.

Although the performance of the correction on the test sets is encouraging, an important question is whether the improved energies are significant enough to produce improved stability rankings of organic crystals. To evaluate the influence on ranking, we applied the correction to all of the CSP landscapes, re-ranking all the predicted structures based on the corrected energies. Thereafter, we compared the ranking of the matches to the experimental structures in terms of both their ranking and their energy above the global minimum with that found using FIT+DMA. Structures with predicted uncertainties greater than 25 kJ mol^−1^ were omitted from this analysis. This amounted to 257 structures (out of 3.9 million total) with examination indicating the structures were predominately high energy structures containing voids.

**Fig. 11:**
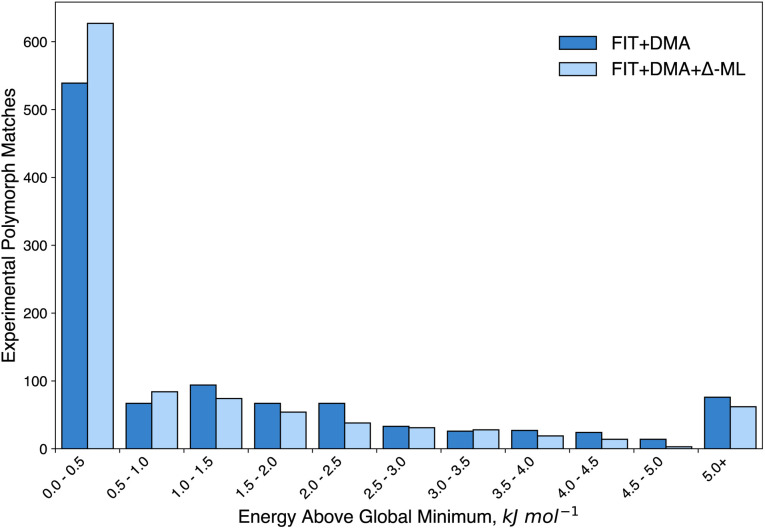
Histogram of the relative energies of matches to the experimentally-determined crystal structures over all CSPs before (FIT+DMA) and after (FIT+DMA+Δ-ML) applying the data-derived B86bPBE+XDM lattice energy correction. Separate distributions for molecules from the training and extrapolation sets are shown in the ESI.†

The resulting distributions (Fig. 11) over both the training and extrapolation compounds illustrate clearly that the corrected energies in general improve the rankings of the experimental structures. For instance, the correction results in an increase from 424 (FIT+DMA) to 501 (FIT+DMA+Δ-ML) of the observed structures ranked as global energy minima, and an increase from 767 (FIT+DMA) to 839 (FIT+DMA+Δ-ML) within 2 kJ mol^−1^ of the global energy minimum (the approximate limit of vibrational contributions to free energy differences). These improvements are observed in the CSP landscapes of the extrapolation and training molecules (see ESI†). Importantly, the correction also greatly improves the worst ranked experimental matches with the proportion ranked above 35 in energy ranking decreasing by over a third, from 69 (FIT+DMA) to 45 (FIT+DMA+Δ-ML) (see ESI†).

#### 3.4.2 Transferable MACE total energy model

While the Δ-ML approach successfully improves the quality of the CSP energy rankings, some of the CSP matches to experimental crystal structures are still relatively high on their energy landscape, even after applying the lattice energy correction. Considering the demonstrated accuracy of the correction, the high relative energies could be the result of limitations with the rigid-body lattice energy minimised geometries, which an energy correction is unable to remedy. Indeed, many of the structures with high relative energies are for compounds which had large geometric deviations between the experimental in-crystal molecular conformations and the gas-phase optimised molecular geometries used (and kept rigid) during CSP. Improving the performance of CSP for these more flexible molecules in our study likely requires re-optimising the predicted crystal structures and relaxing the rigid-molecule constraint.

The FIT+DMA+Δ-ML model is not suitable for this task because it is only a correction to the intermolecular contribution to the lattice energy. Therefore, using a dataset derived from the CSP structures selected for the energy correction model, with perturbed atomic coordinates to sample conformational degrees of freedom, we trained a total energy MACE (higher order equivariant message passing neural network) model. Full details are provided in the ESI.† The trained MACE model was then applied to geometry optimise the predicted structures of 15 compounds with large differences in molecular geometry between the observed crystal structure and the DFT-optimised molecule (see Fig. 1, bottom three rows).

**Fig. 12:**
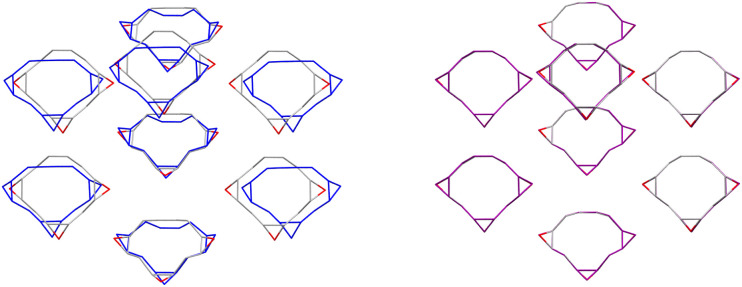
Overlay of the experimentally determined crystal structure (atoms coloured by element) of (*exo*,*exo*,*exo*)-1,2:4,5:7,8-triepoxycyclododec-10-ene (CSD reference code WACYEF) with: (left) the matching structure from the force field (FIT+DMA) CSP (blue); and (right) the matching CSP structure after re-optimisation with the transferable MACE model (purple). Hydrogen atoms are hidden for clarity. The large structural deviation in the FIT+DMA structure is driven by deviation in the molecular geometry.

Re-optimisation with the trained MACE model yielded considerable improvements in the geometric agreement of predicted structures with experiment and of their energy ranking on the CSP landscapes (Table 2). RMSD_30_ between experimental and predicted structures decreased upon re-optimisation for 14 of the 15 compounds, by up to 1.4 Å, and moved the match to the observed structure closer to the global energy minimum in all but 2 cases, with 7 becoming the global energy minimum structure. Fig. 12 illustrates the improved geometric agreement for one of these molecules.

**Table 2:** Crystal structure RMSD_30_ and energetic ranking of matches to the experimentally-determined crystal structures within CSP landscapes for 15 molecules re-optimised with the transferable MACE model. Changes between the rigid-molecule force field results (FIT+DMA) and MACE are shown in parentheses. Bold entries highlight where MACE re-optimisation leads to improvement

Crystal structure	FIT+DMA		MACE	
	RMSD_30_ (Å)	Δ*E* (kJ mol^−1^)	RMSD_30_ (Å)	Δ*E* (kJ mol^−1^)
ATCDEO	0.341	7.26	**0.125 (−0.216)**	**6.67 (−0.59)**
BIRTUR	0.396	6.06	**0.391 (−0.005)**	**0.02 (−6.03)**
BIXKUP	0.813	16.32	**0.208 (−0.605)**	**0.00 (−16.32)**
BIXLOK	1.048	4.95	**0.265 (−0.783)**	**0.00 (−4.95)**
DALBIC	0.343	1.45	**0.206 (−0.137)**	3.70 **(**+2.25)
DEBFOI	1.208	4.18	**0.110 (−1.098)**	**0.00 (−4.18)**
DOBYOJ	0.654	1.27	**0.640 (−0.014)**	3.55 **(**+2.28)
JASGIT	1.837	15.77	**1.776 (−0.061)**	**9.72 (−6.05)**
KOGFER	0.247	0.37	0.248 **(**+0.001)	**0.00 (−0.37)**
QEKQIG	0.824	4.84	**0.139 (−0.684)**	**0.00 (−4.84)**
TAVTUH	1.751	12.03	**0.325 (−1.426)**	**1.87 (−10.16)**
TUNWUV	1.103	9.55	**0.399 (−0.704)**	**0.00 (−9.55)**
UDEXUZ	0.725	7.41	**0.216 (−0.509)**	**0.75 (−6.66)**
WACYAB	0.721	9.81	**0.216 (−0.505)**	**0.92 (−8.89)**
WACYEF	0.975	14.02	**0.200 (−0.775)**	**0.00 (−14.02)**

Re-optimisation of CSP structures was also run for 4,8*b*-dihydropyrrolo[3,4-*b*]indole-1,3(2*H*,3a*H*)-dione, where no match to the experimental crystal structure (CSD reference code BUVGAC) was identified in the CSP. However, no match was identified after re-optimisation with the MACE model; in this case, molecular flexibility would have been required during structure generation, rather than post-CSP re-optimisation. Now that a transferable MACE model has been trained, it could potentially be implemented earlier in the CSP workflow.

It should be noted though that the MACE model is a semi-local model and so neglects long-range interactions that can be important for highly accurate relative energies. Overall, the results of the unconstrained optimisations confirm that the high relative energies of these crystal structure matches estimated by FIT+DMA+Δ-ML are largely a result of limitations with the rigid-body lattice energy minimised geometries. It is worth reiterating that the limitations of the CSP geometries only affected a small number of structures; in the vast majority of cases, as shown earlier, the predicted structures at the FIT+DMA level achieved high quality matches to the experimentally-determined crystal structures. Undoubtedly, the success of the FIT+DMA+Δ-ML energy correction is a reflection of the performance of the baseline empirical force field.

## 4 Conclusions

We have presented (to our knowledge) the largest CSP dataset produced to-date, serving as a computational survey of crystal packing in the organic solid state for small, rigid molecules. Using established, well-characterised CSP methods, we produced crystal structure energy landscapes for over 1000 such molecules, all of which have at least one known, solved crystal structure available in the CSD. In total, our CSP landscapes contain over 4 million unique crystal structures, each with an associated lattice energy at a consistent level of theory.

We have assessed the quality of the dataset by evaluating its reliability at predicting the known crystal structures of these molecules, both in terms of the quality of the geometric match of the crystal structures and the resulting energy ranking of the experimental forms on their respective landscapes compared to other hypothetical structures. Our CSP approach is overwhelmingly successful at predicting crystal structures of these simple molecules – over 99% of all experimental structures have a match located in our CSP searches. 41% of experimental structures are predicted to be the global energy minimum on their landscapes, and 74% are found within 2 kJ mol^−1^ of this minimum, a margin equivalent to the estimated error introduced by ignoring thermal effects and ranking based solely on static, 0 K lattice energies. Geometrically, the typical discrepancy between experimental structures and their closest predicted matches is comparable to the thermal fluctuations in experimental crystal structure solutions of the same solid form obtained at low temperatures *versus* ambient conditions. Such remarkable performance demonstrates the consistency and accuracy of our chosen methods for optimising and ranking these structures.

Such a large dataset of many possible crystal packings should prove a valuable resource for identifying a variety of crystal packing trends, and we make this data available to the community as part of this work. Herein, we studied space group distributions among low-energy hypothetical structures compared to those observed in the CSD, and find substantial overlap, particularly among the most common space groups. We also demonstrated the potential of this varied dataset to explore chirality in the organic solid state, finding very good agreement with established empirical rules concerning the propensity of racemic mixtures to crystallise in racemic crystal structures as opposed to separate enantiopure crystals.

Additionally, we have shown the power of such large-scale CSP to train transferable machine learned potentials for organic solid-state systems. A committee neural network potential trained on single-point periodic DFT lattice energies achieved excellent accuracy in correcting our force field energy landscapes to the DFT level, reducing energy errors by approximately 8 fold. While the NNP performance is reduced on molecules reserved as an extrapolation set compared to those seen in training, the potential still demonstrates improvements to the quality of the resulting CSP rankings increasing the number of experimental structures ranked as the global energy minimum by 18% overall. We further demonstrated the development of a transferable MACE potential using structures derived from the CSP landscapes to allow re-optimisation of crystal structures, testing it successfully on those molecules in our set where the molecular geometry distortion between the conformation in the known crystal structure and the gas-phase-optimised one was largest. The results showed improved structural agreement with experimental structures in almost all cases and much improved energy rankings, moving several poorly-ranked observed structures to the global energy minimum.

Using these mature, well-tested CSP methods alongside modern machine learning approaches, we have demonstrated the ability of CSP to create very large, diverse datasets of hypothetical crystal structures, and the utility of this information in both understanding broad trends in organic crystal structures and in training more advanced energetic models for refinement and transferability. It is our hope that the variety and quantity of CSP data presented here, alongside our demonstrations of possible applications, enables the greater organic solid-state computational community to develop even more sophisticated models and techniques in pursuit of truly predictive, computational data-driven discovery of novel materials.

## Author contributions

Christopher R. Taylor: conceptualisation, methodology, software, investigation, validation, formal analysis, data curation, writing – original draft, writing – review & editing. Led on production, curation and analysis of large-scale CSP datasets. Patrick W. V. Butler: methodology, software, investigation, validation, formal analysis, writing – original draft, writing – review & editing. Training of ML energy models and their evaluation. Graeme M. Day: conceptualisation, validation, formal analysis, resources, writing – original draft, writing – review & editing, supervision, funding acquisition.

## Conflicts of interest

There are no conflicts to declare.

## Supplementary Material

FD-256-D4FD00105B-s001

## References

[cit1] Groom C. R., Bruno I. J., Lightfoot M. P., Ward S. C. (2016). Acta Crystallogr., Sect. B: Struct. Sci., Cryst. Eng. Mater..

[cit2] Zagorac D., Müller H., Ruehl S., Zagorac J., Rehme S. (2019). J. Appl. Crystallogr..

[cit3] Treacy M. M. J., Rivin I., Balkovsky E., Randall K. H., Foster M. D. (2004). Microporous Mesoporous Mater..

[cit4] Le Bail A. (2005). J. Appl. Crystallogr..

[cit5] Gražulis S., Chateigner D., Downs R. T., Yokochi A. F. T., Quirós M., Lutterotti L., Manakova E., Butkus J., Moeck P., Le Bail A. (2009). J. Appl. Crystallogr..

[cit6] Vaitkus A., Merkys A., Gražulis S. (2021). J. Appl. Crystallogr..

[cit7] Vaitkus A., Merkys A., Sander T., Quirós M., Thiessen P. A., Bolton E. E., Gražulis S. (2023). J. Cheminf..

[cit8] Jain A., Ong S. P., Hautier G., Chen W., Richards W. D., Dacek S., Cholia S., Gunter D., Skinner D., Ceder G., Persson K. A. (2013). APL Mater..

[cit9] Nyman J., Day G. M. (2016). Phys. Chem. Chem. Phys..

[cit10] Nyman J., Day G. M. (2015). CrystEngComm.

[cit11] Cruz-Cabeza A. J., Bernstein J. (2014). Chem. Rev..

[cit12] Cruz-Cabeza A. J., Reutzel-Edens S. M., Bernstein J. (2015). Chem. Soc. Rev..

[cit13] Sacchi P., Neoptolemou P., Davey R. J., Reutzel-Edens S. M., Cruz-Cabeza A. J. (2023). Chem. Sci..

[cit14] Thompson H. P. G., Day G. M. (2014). Chem. Sci..

[cit15] Chan H. C. S., Kendrick J., Neumann M. A., Leusen F. J. J. (2013). CrystEngComm.

[cit16] Taylor C. R., Day G. M. (2018). Cryst. Growth Des..

[cit17] LeBlanc L. M., Dale S. G., Taylor C. R., Becke A. D., Day G. M., Johnson E. R. (2018). Angew. Chem., Int. Ed..

[cit18] Hoja J., Tkatchenko A. (2018). Faraday Discuss..

[cit19] Day G. M. (2011). Crystallogr. Rev..

[cit20] Beran G. J. O. (2023). Chem. Sci..

[cit21] Yang S., Day G. M. (2022). Commun. Chem..

[cit22] Francia N. F., Price L. S., Nyman J., Price S. L., Salvalaglio M. (2020). Cryst. Growth Des..

[cit23] Butler P. W. V., Day G. M. (2023). Proc. Natl. Acad. Sci. U. S. A..

[cit24] Pulido A., Chen L., Kaczorowski T., Holden D., Little M. A., Chong S. Y., Slater B. J., McMahon D. P., Bonillo B., Stackhouse C. J., Stephenson A., Kane C. M., Clowes R., Hasell T., Cooper A. I., Day G. M. (2017). Nature.

[cit25] Campbell J. E., Yang J., Day G. M. (2017). J. Mater. Chem. C.

[cit26] Day G. M., Cooper A. I. (2018). Adv. Mater..

[cit27] Zhao C., Chen L., Che Y., Pang Z., Wu X., Lu Y., Liu H., Day G. M., Cooper A. I. (2021). Nat. Commun..

[cit28] Case D. H., Campbell J. E., Bygrave P. J., Day G. M. (2016). J. Chem. Theory Comput..

[cit29] Karamertzanis P. G., Pantelides C. C. (2005). J. Comput. Chem..

[cit30] Curtis F., Li X., Rose T., Vásquez-Mayagoitia A., Bhattacharya S., Ghiringhelli L. M., Marom N. (2018). J. Chem. Theory Comput..

[cit31] Neumann M. A., Streek J. v. d., Fabbiani F. P. A., Hidber P., Grassmann O. (2015). Nat. Commun..

[cit32] Bučar D.-K., Day G. M., Halasz I., Zhang G. G. Z., Sander J. R. G., Reid D. G., MacGillivray L. R., Duer M. J., Jones W. (2013). Chem. Sci..

[cit33] Taylor C. R., Mulvee M. T., Perenyi D. S., Probert M. R., Day G. M., Steed J. W. (2020). J. Am. Chem. Soc..

[cit34] Case D. H., Srirambhatla V. K., Guo R., Watson R. E., Price L. S., Polyzois H., Cockcroft J. K., Florence A. J., Tocher D. A., Price S. L. (2018). Cryst. Growth Des..

[cit35] Reilly A. M., Cooper R. I., Adjiman C. S., Bhattacharya S., Boese A. D., Brandenburg J. G., Bygrave P. J., Bylsma R., Campbell J. E., Car R., Case D. H., Chadha R., Cole J. C., Cosburn K., Cuppen H. M., Curtis F., Day G. M., DiStasio Jr R. A., Dzyabchenko A., van Eijck B. P., Elking D. M., van den Ende J. A., Facelli J. C., Ferraro M. B., Fusti-Molnar L., Gatsiou C.-A., Gee T. S., de Gelder R., Ghiringhelli L. M., Goto H., Grimme S., Guo R., Hofmann D. W. M., Hoja J., Hylton R. K., Iuzzolino L., Jankiewicz W., de Jong D. T., Kendrick J., de Klerk N. J. J., Ko H.-Y., Kuleshova L. N., Li X., Lohani S., Leusen F. J. J., Lund A. M., Lv J., Ma Y., Marom N., Masunov A. E., McCabe P., McMahon D. P., Meekes H., Metz M. P., Misquitta A. J., Mohamed S., Monserrat B., Needs R. J., Neumann M. A., Nyman J., Obata S., Oberhofer H., Oganov A. R., Orendt A. M., Pagola G. I., Pantelides C. C., Pickard C. J., Podeszwa R., Price L. S., Price S. L., Pulido A., Read M. G., Reuter K., Schneider E., Schober C., Shields G. P., Singh P., Sugden I. J., Szalewicz K., Taylor C. R., Tkatchenko A., Tuckerman M. E., Vacarro F., Vasileiadis M., Vazquez-Mayagoitia A., Vogt L., Wang Y., Watson R. E., de Wijs G. A., Yang J., Zhu Q., Groom C. R. (2016). Acta Crystallogr., Sect. B: Struct. Sci., Cryst. Eng. Mater..

[cit36] Behler J., Csányi G. (2021). Eur. Phys. J. B.

[cit37] Musil F., De S., Yang J., Campbell J. E., Day G. M., Ceriotti M. (2018). Chem. Sci..

[cit38] McDonagh D., Skylaris C.-K., Day G. M. (2019). J. Chem. Theory Comput..

[cit39] Egorova O., Hafizi R., Woods D. C., Day G. M. (2020). J. Phys. Chem. A.

[cit40] Butler P. W. V., Hafizi R., Day G. M. (2024). J. Phys. Chem. A.

[cit41] Kapil V., Engel E. A. (2022). Proc. Natl. Acad. Sci. U. S. A..

[cit42] Kovács D. P. , Moore J. H., Browning N. J., Batatia I., Horton J. T., Kapil V., Witt W. C., Magdău I.-B., Cole D. J. and Csányi G., MACE-OFF23: Transferable Machine Learning Force Fields for Organic Molecules, arXiv, 2023, preprint, arXiv:2312.15211[physics], 10.48550/arXiv.2312.15211 PMC12123624 40387214

[cit43] Paruzzo F. M., Hofstetter A., Musil F., De S., Ceriotti M., Emsley L. (2018). Nat. Commun..

[cit44] Galek P. T. A., Fábián L., Motherwell W. D. S., Allen F. H., Feeder N. (2007). Acta Crystallogr., Sect. B: Struct. Sci..

[cit45] Cersonsky R. K., Pakhnova M., Engel E. A., Ceriotti M. (2023). Chem. Sci..

[cit46] Yang J., De S., Campbell J. E., Li S., Ceriotti M., Day G. M. (2018). Chem. Mater..

[cit47] Frisch M. J. , Trucks G. W., Schlegel H. B., Scuseria G. E., Robb M. A., Cheeseman J. R., Scalmani G., Barone V., Mennucci B., Petersson G. A., Nakatsuji H., Caricato M., Li X., Hratchian H. P., Izmaylov A. F., Bloino J., Zheng G., Sonnenberg J. L., Hada M., Ehara M., Toyota K., Fukuda R., Hasegawa J., Ishida M., Nakajima T., Honda Y., Kitao O., Nakai H., Vreven T., Montgomery Jr J. A., Peralta J. E., Ogliaro F., Bearpark M., Heyd J. J., Brothers E., Kudin K. N., Staroverov V. N., Kobayashi R., Normand J., Raghavachari K., Rendell A., Burant J. C., Iyengar S. S., Tomasi J., Cossi M., Rega N., Millam J. M., Klene M., Knox J. E., Cross J. B., Bakken V., Adamo C., Jaramillo J., Gomperts R., Stratmann R. E., Yazyev O., Austin A. J., Cammi R., Pomelli C., Ochterski J. W., Martin R. L., Morokuma K., Zakrzewski V. G., Voth G. A., Salvador P., Dannenberg J. J., Dapprich S., Daniels A. D., Farkas Ö., Foresman J. B., Ortiz J. V., Cioslowski J. and Fox D. J., Gaussian09 Revision D.01

[cit48] Lee C., Yang W., Parr R. G. (1988). Phys. Rev. B: Condens. Matter Mater. Phys..

[cit49] Becke A. D. (1993). J. Chem. Phys..

[cit50] Raghavachari K., Binkley J. S., Seeger R., Pople J. A. (1980). J. Chem. Phys..

[cit51] Grimme S., Antony J., Ehrlich S., Krieg H. (2010). J. Chem. Phys..

[cit52] Becke A. D., Johnson E. R. (2005). J. Chem. Phys..

[cit53] Stone A. J., Alderton M. (2002). Mol. Phys..

[cit54] Stone A. J. (2005). J. Chem. Theory Comput..

[cit55] Winn P. J., Ferenczy G. G., Reynolds C. A. (1997). J. Phys. Chem. A.

[cit56] Ferenczy G. G., Winn P. J., Reynolds C. A. (1997). J. Phys. Chem. A.

[cit57] Coombes D. S., Price S. L., Willock D. J., Leslie M. (1996). J. Phys. Chem..

[cit58] Price S. L., Leslie M., Welch G. W. A., Habgood M., Price L. S., Karamertzanis P. G., Day G. M. (2010). Phys. Chem. Chem. Phys..

[cit59] Williams D. E., Houpt D. J. (1986). Acta Crystallogr., Sect. B: Struct. Sci..

[cit60] Chisholm J. A., Motherwell S. (2005). J. Appl. Crystallogr..

[cit61] RDKit: Open-Source Cheminformatics, https://www.rdkit.org

[cit62] Peifer C., Tschertsche M., Schollmeyer D., Laufer S. (2007). Acta Crystallogr., Sect. E: Struct. Rep. Online.

[cit63] Wilson C. (2000). Z. Kristallogr. – Cryst. Mater..

[cit64] Buerger M. J. (1962). Science.

[cit65] Aitchison C. M., Kane C. M., McMahon D. P., Spackman P. R., Pulido A., Wang X., Wilbraham L., Chen L., Clowes R., Zwijnenburg M. A., Sprick R. S., Little M. A., Day G. M., Cooper A. I. (2020). J. Mater. Chem. A.

[cit66] Brock C. P., Dunitz J. D. (1994). Chem. Mater..

[cit67] Wallach O. (1895). Justus Liebigs Ann. Chem..

[cit68] Brock C. P., Schweizer W. B., Dunitz J. D. (1991). J. Am. Chem. Soc..

[cit69] Clements R. J., Dickman J., Johal J., Martin J., Glover J., Day G. M. (2022). MRS Bull..

[cit70] Behler J. (2011). J. Chem. Phys..

[cit71] Gastegger M., Schwiedrzik L., Bittermann M., Berzsenyi F., Marquetand P. (2018). J. Chem. Phys..

