# Supplementary material for: Predictive crystallography at scale: mapping, validating, and learning from 1000 crystal energy landscapes
Source: Faraday Discuss. 2024 Jun 3;256:434–58. doi: 10.1039/d4fd00105b (PMC11413732; doi:10.1039/d4fd00105b)
Supplement: FD-256-D4FD00105B-s001 [file FD-256-D4FD00105B-s001.pdf]

Electronic Supplementary Material (ESI) for Faraday Discussions.
This journal is © The Royal Society of Chemistry 2024

Predictive crystallography at scale: mapping, validating, and learning from 1,000 crystal energy landscapes—Supporting Information

Christopher R. Taylor[a], Patrick W. V. Butler[a], and Graeme M. Day[a*]
[a]School of Chemistry, University of Southampton, Southampton, SO17 1BJ, UK
*Corresponding author: g.m.day@soton.ac.uk

Contents

S1 Computational Details **S3**
S1.1 Crystal Structure Generation and Optimisation S3
S1.2 Committee Neural Network Potentials S5
S1.3 MACE Potentials S5
S1.4 Lattice Energy Correction Dataset S5
S1.5 PBE+D3 Total Energy Dataset S6

S2 Results **S7**
S2.1 Learning Curves of Δ-ML Lattice Energy Correction S7
S2.2 Compound Distribution in Training Dataset S7
S2.3 Lattice Energy Errors S8
S2.4 Δ-ML Lattice Energy Corrected Experimental Structure Ranking S9

S3 Molecular Data **S10**

References **S12**

S1 Computational Details

S1.1 Crystal Structure Generation and Optimisation

CSP was performed using the Global Lattice Energy Explorer (GLEE) package,[1] which uses quasi-random sampling of crystal packing variables to generate trial crystal structures uniformly distributed across the lattice energy landscape, followed by rigid-molecule lattice energy minimisation using an anisotropic atom-atom intermolecular force field. All resulting local energy minima are treated as possible crystal structures of the molecule.

For a given molecular conformer, we generate quasi-random crystal packings using a Sobol low-discrepancy sequence to sample molecular positions and orientations, and unit cell lengths and angles (subject to space group symmetry constraints). Successful trial structures are those that satisfy simple geometric requirements of avoiding overlap between atomic van der Waals' radii while maintaining reasonably dense packing (i.e. limiting the volume of the unit cell as a multiple of the molecular van der Waals' volume, $V_{\text{cell}} \leq 2.5 \times Z \times V_{\text{molecule}}$, where Z is the number of molecules in the unit cell).

Space group symmetry is used to reduce the dimensionality of the search space, so that only the position and orientation of molecules in the asymmetric unit are sampled, with all other molecules in the unit cell generated by symmetry. In this study, we restrict ourselves to generating crystal structures with one independent molecule in the asymmetric unit ($Z' = 1$). We sampled the 26 most commonly observed space groups for organic molecular crystals, listed in Table S1. These space groups cover over 99.4% of $Z' \leq 1$ structures in the CSD.

A very small minority of molecules in our set (9 out of 1007) crystallise in uncommon symmetries whose $Z' = 1$ equivalent structures would not be found in the 26 most common space groups. We elected to run additional, ad hoc sampling for these molecules only in the space groups that (in conjunction with an asymmetric unit of $Z' = 1$) could lead to the experimental structure. The additional space groups and the molecules for which the additional sampling was performed are shown in Table S2.

The space groups were sampled equally, irrespective of their observed frequency in the CSD: quasi-random structures are generated and lattice energy minimised in each space group until 10,000 successfully energy minimised crystal structures were found (260,000 crystal structures per molecule). The CSP process is highly parallelisable, as each crystal structure structure is independent.

Trial crystal that passed geometric checks were lattice energy-minimised in a three-stage process. Non-electrostatic interactions (principally intermolecular dispersion and exchange-repulsion) were described by the FIT $exp - 6$ force field [2, 3], supplemented by fluorine parameters from Williams and Houpt[4] with F...X heteroatomic interaction parameters derived from combining rules applied to the parameters for homoatomic interactions: geometric mean values are used for the pre-exponential and dispersion parameters, and arithmetic mean for the length-scale in the exponential repulsion term.

Firstly, structures were optimised with PMIN [5] with fixed unit cell angles to avoid very acute unit cell angles being reached during optimisation. The resulting crystal structures were re-optimised using DMACRYS [3] with a small external pressure of 0.1 GPa to assist with convergence, allowing all crystalline degrees of freedom (that preserve the space group symmetry) to adjust. Atomic point charge electrostatics was used in these first two stages. Finally, the resulting structures were re-optimised in DMACRYS, now using the full set of atomic multipoles and no external pressure to yield the final crystal structures and their energy ranking on the landscape.

It is commonplace that multiple unique initial configurations optimise to the same local energy minimum. We remove these duplicates via a two-step procedure. A initial, fast identification of duplicate crystal structures was performed by comparing simulated powder X-ray diffraction (PXRD) patterns (generated in the PLATON package) [6] using a constrained dynamic time-warping approach we have

employed previously [7], using strict comparison criteria to remove only near-identical structures. We then further filter this set based on structural comparisons using the COMPACK [8] algorithm as implemented in the CSD Python API, overlaying 30-molecule clusters of the crystal structures being compared. For efficiency, only structures within 1.0 kJ mol^{-1} of lattice energy and 0.05 g cm^{-3} in density were compared; structures were considered duplicates if interatomic distances and angles within 30-molecule clusters matched using distance and angle tolerances of 30% and 30° respectively. In both duplicate removal steps, we choose to retain the lowest-energy representative of a group of duplicate structures.

IUCr Tables Number	Short Symbol
1	$P1$
2	$P\bar{1}$
4	$P2_1$
5	$C2$
7	Pc
9	Cc
13	$P2/c$
14	$P2_1/c$
15	$C2/c$
18	$P2_12_12$
19	$P2_12_12_1$
20	$C222_1$
29	$Pca2_1$
33	$Pna2_1$
43	$Fdd2$
56	$Pccn$
60	$Pbcn$
61	$Pbca$
76	$P4_1$
86	$P4_2/n$
88	$I4_1/a$
96	$P4_32_12$
145	$P3_2$
148	$R3$
154	$P3_221$
169	$P6_1$

Table S1: The list of space groups sampled in all CSPs for all molecules in our set, ordered in accordance with their IUCr Tables number. Note that, due to our CSP workflow requiring asymmetric units of $Z' = 1$, meaning that the appropriate space group to locate an experimental match is not necessarily the space group assigned in the CSD (i.e. if the CSD structure has $Z' < 1$).

IUCr Tables Number	Short Symbol	Compounds
18	$P2_12_12$	MEGNES
45	$Iba2$	GEYFOF, NTCHPE
92	$P4_12_12$	IHEPUG, QEGSOL
110	$I4_1cd$	NICREW
114	$P\bar{4}2_1c$	CUDJIU01, CUMJOJ
122	$I\bar{4}2d$	JEZZOD

Table S2: The list of uncommon space groups that were additionally sampled on an ad-hoc basis for a small number of compounds whose experimental crystal symmetries would not be covered by the general set in Table S1, with the list of compounds for which each space group was included (named according to their CSD refcode).

S1.2 Committee Neural Network Potentials

All NNPs that constituted our lattice energy correction were developed with the n2p2 package[9, 10] and used an input of weighted atom-centred symmetry functions (ACSFs)[11, 12] with a radial cutoff of 8.0 Å. A total of 64 radial and angular symmetry functions were used per element, with the ACSFs determined by a CUR decomposition using the data from a large pool of ACSFs calculated on 30,000 randomly selected structures from the CSPs. The neural network architecture of the NNPs consisted of two hidden layers containing 30 neurons each. In all cases the softplus activation function was used in the hidden layers. The network parameters were initialised by the Nguyen-Widrow scheme and were subsequently optimised during training by the Kalman filter method. A 90:10 train:validation split was used throughout and the parameters from the epoch with the lowest validation energy MAE were selected following training. For each member of the ensemble the random number generator for initialising the parameters and splitting the dataset was independently seeded.

The full NNP-corrected CSP landscapes and the NNP model itself are available from DOI: 10.5258/SOTON/D3094.

S1.3 MACE Potentials

For computational efficiency in the geometry optimisations small MACE[13] models were trained consisting of 96 channels, max L of 0, and a radial cutoff of 4.5 Å. The number of message passing layers was set to 2 and the body-order to 4. The models were trained on total energies and forces with the ScaleShiftMACE model type and swa strategy. A 90:10 train:validation split was employed and the parameters were optimised using the ADAM method. Geometry optimisations of CSP structures using the MACE models were conducted through ASE[14] using the preconLBFGS optimizer and the ExpCellFilter to allow relaxation of the cell parameters. The optimisations were continued until the max atomic force was less than 0.005 eV Å^{-1}.

S1.4 Lattice Energy Correction Dataset

The dataset for training the lattice energy correction consists of crystal structures sampled from the CSP landscapes and the corresponding energy corrections between the FIT+DMA and the B86bPBE+XDM lattice energies. The energy correction was calculated as follows

$$\Delta E_{lattice} = E_{lattice}^{B86bPBE+XDM} - E_{lattice}^{FIT+DMA} \tag{1}$$

Where the B86bPBE+XDM lattice energies are calculated by removing the isolated molecular energy of all N molecules in the unit cell from the total crystal energy,

$$E_{lattice}^{B86bPBE+XDM} = E_{total}^{B86bPBE+XDM} - \sum^{N} E_{molecule,i}^{B86bPBE+XDM} \tag{2}$$

In all cases the B86bPBE+XDM calculations were conducted using the FHI-aims code[15–17] with a tight basis set of numeric atom-centred orbitals and total energy and force convergence criteria of 10^{-6} eV and 10^{-4} eV/Å, respectively. For the total energy calculations of crystal structures a k-point grid with spacing of 0.05 Å^{-1} was used.

The initial dataset was created by randomly sampling 10 structures from each CSP landscape with relative energy less than 8.0 kJ mol^{-1}. In cases where 10 structures could not be sampled (due to there being less than 10 structures with relative energies less than 8.0 kJ mol^{-1}), all the low energy structures were selected. As stated in the main text this initial dataset was partitioned into a training set, an in-domain test set, and an extrapolation test set. The training and test set contained structures for 891

compounds while the extrapolation set contained all the sampled structures for 143 compounds. It should be noted that the datasets for the ML potential contains 23 compounds not present in the CSP results. This is because these were molecules selected for which the known crystal structures could not have been matched given our CSP assumptions (limits on space group and Z'); we include them in NNP training as their landscapes represent plausible Z'=1 structures of molecules observed to crystallise in the CSD that satisfy our other chemical criteria.

An initial committee of 8 NNPs was trained, and thereafter, we performed two iterations of active learning on the remaining low energy structures of the training compounds. This involved selecting the 500 highest uncertainty structures at each iteration and adding them to the training dataset before retraining the model to update the uncertainties. Using query-by-committee, the uncertainties were estimated as the standard deviation of the energy predictions of the 8 NNPs. Notably, we converted the energies and the standard deviations to units of $kJ\ mol^{-1}\ \sqrt{N}^{\ -1}$ where N is the number of atoms in the structure. This accounts for difference in the size of the molecules, which is not accounted for by the conventional units of kJ mol^{-1} (per molecule). Overall the two iterations of active learning resulted in an additional 1000 structures being added to the training set. The active learning was terminated after two iterations since we found that the improvement to the errors on the test and extrapolation sets was small and thus likely no longer affected the re-ranking of structures significantly.

To assess whether the energy cutoff was limiting the model, we conducted a third iteration of active learning across all remaining candidates of the training compounds (i.e. without a relative energy cutoff). However, this did not offer much improvement and even slightly reduced performance on the lowest energy structures. Consequently, the second iteration model was used in the reported results. The learning curves over the active learning iterations is presented in Figure S1.

The MACE re-optimised CSP landscapes and the MACE model itself are available from DOI: 10.5258/SOTON/D3094.

S1.5 PBE+D3 Total Energy Dataset

The dataset for training MACE models used in unconstrained geometry optimisations was derived from the entire lattice energy correction dataset. Specifically, the atom coordinates of 11,325 structures were randomly perturbed to generate structures with greater conformational diversity. The perturbations were conducted through the ASE python package[14] using a standard deviation of 0.03 Å. The resulting structures were then evaluated with DFT using the PBE exchange-correlation functional and D3(BJ)[18] dispersion correction (PBE+D3) as implemented in VASP[19–22]. For all calculations, a 600 eV cutoff was set on the planewave basis set and a maximum k-point spacing of 0.05 $Å^{-1}$ was used.

While models trained solely on rattled CSP structures are robust for unconstrained geometry optimisations, we have observed that the predicted relative energies can have high errors. Training a second iteration model with a dataset augmented by structures optimised with the first iteration model can reduce these errors significantly. Hence, we applied this here, randomly selecting 5 CSP structures per compound with relative energies less than 8.0 kJ mol^{-1} and optimising them with an initial MACE model trained on the dataset of only rattled structures. Energies and forces were evaluated for these optimised structures with the same PBE+D3 method and combined with the previous dataset. This procedure resulted in the second iteration dataset containing 16,271 structures. The second iteration MACE model trained on this dataset was used in to perform the reported unconstrained geometry optimisations.

S2 Results

S2.1 Learning Curves of Δ-ML Lattice Energy Correction

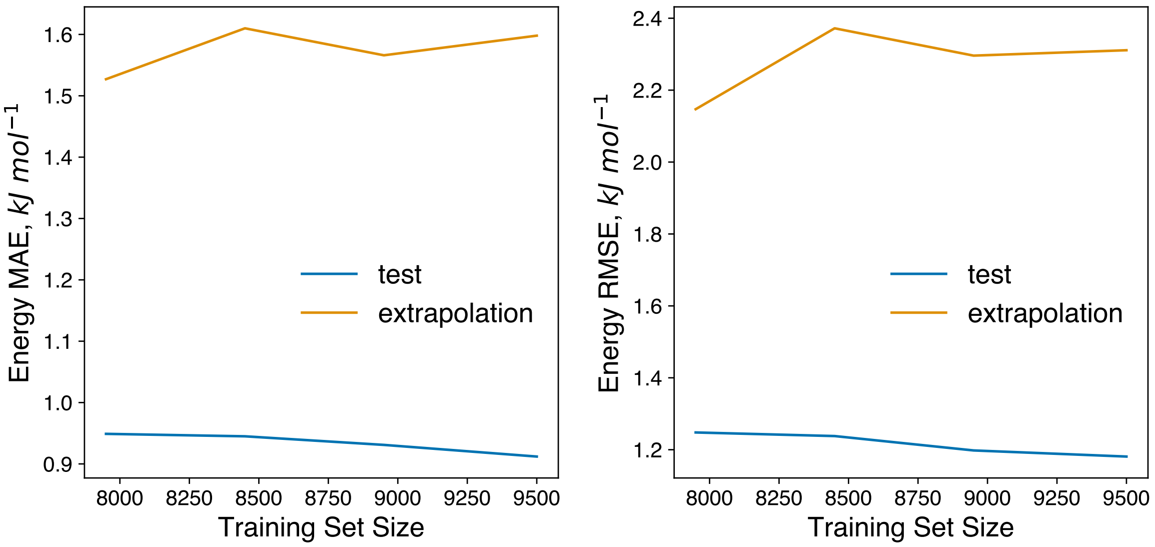

Figure S1: The MAE (left) and RMSE (right) learning curves for the FIT+DMA+Δ-ML model on the (in-domain) test and extrapolation sets. Includes the initial random sampling iteration and 3 iterations of active learning.

S2.2 Compound Distribution in Training Dataset

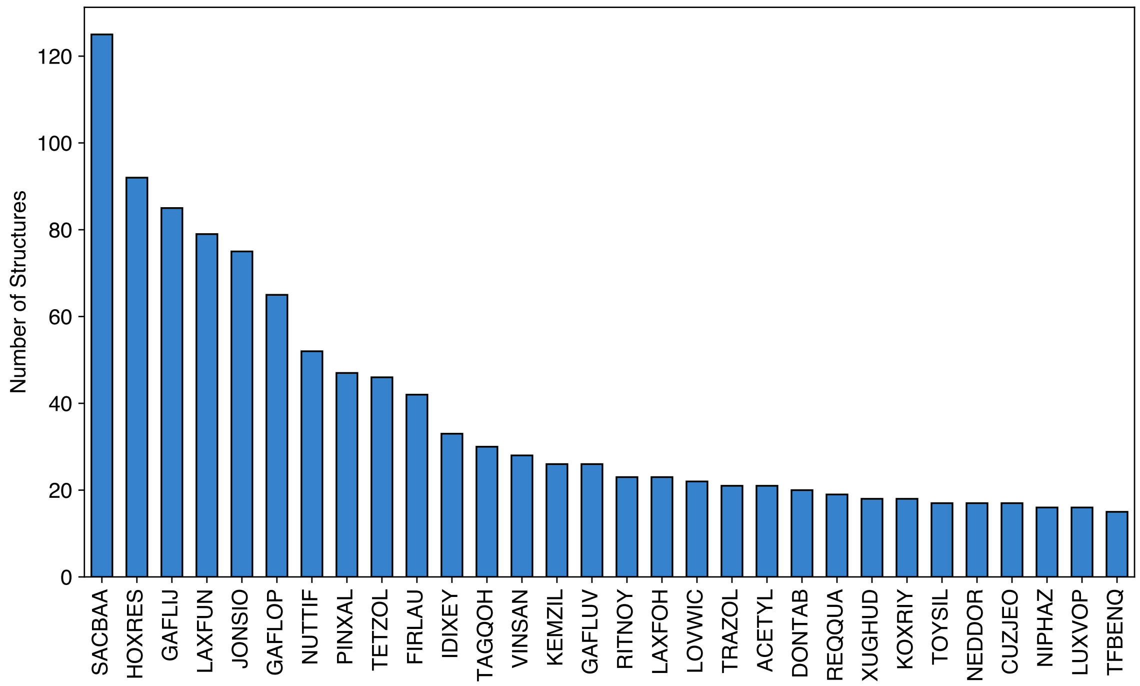

Figure S2: The number of structures for the 30 most prominent compounds in the training set following the initial sampling and two iterations of active learning (8950 total structures). Notably 4 out of the top 5 are compounds containing fluorine functional groups.

S2.3 Lattice Energy Errors

FIT+DMA

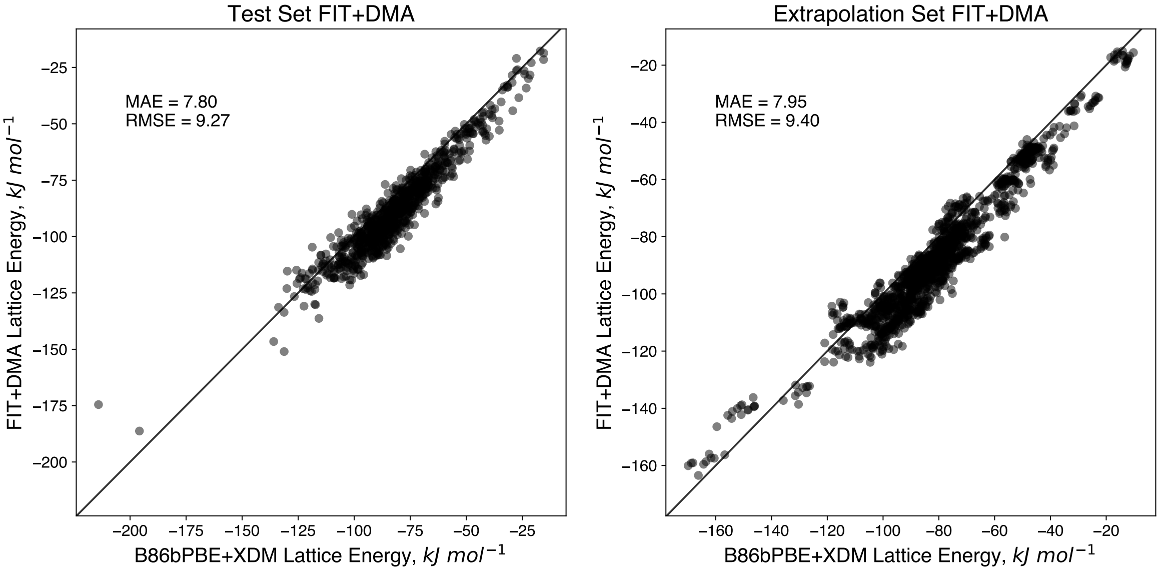

Figure S3: Lattice energy errors of FIT+DMA for the test (left) and extrapolation (right) sets relative to B86bPBE+XDM lattice energies.

FIT+DMA+Δ-ML

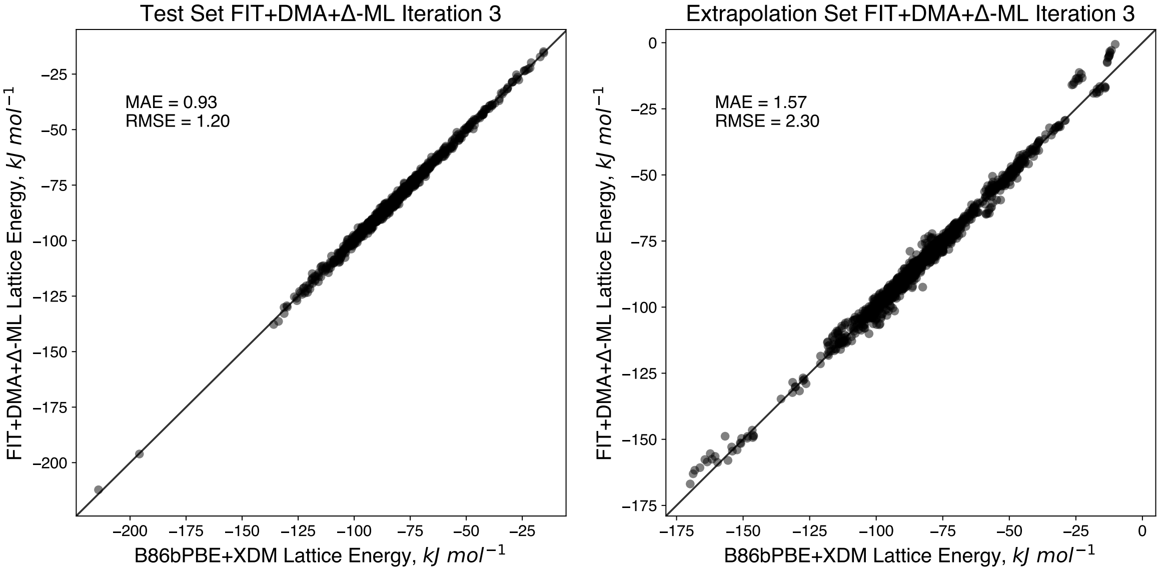

Figure S4: Lattice energy errors of FIT+DMA+Δ-ML (iteration 3) for the test (left) and extrapolation (right) sets relative to B86bPBE+XDM lattice energies.

S2.4 Δ-ML Lattice Energy Corrected Experimental Structure Ranking

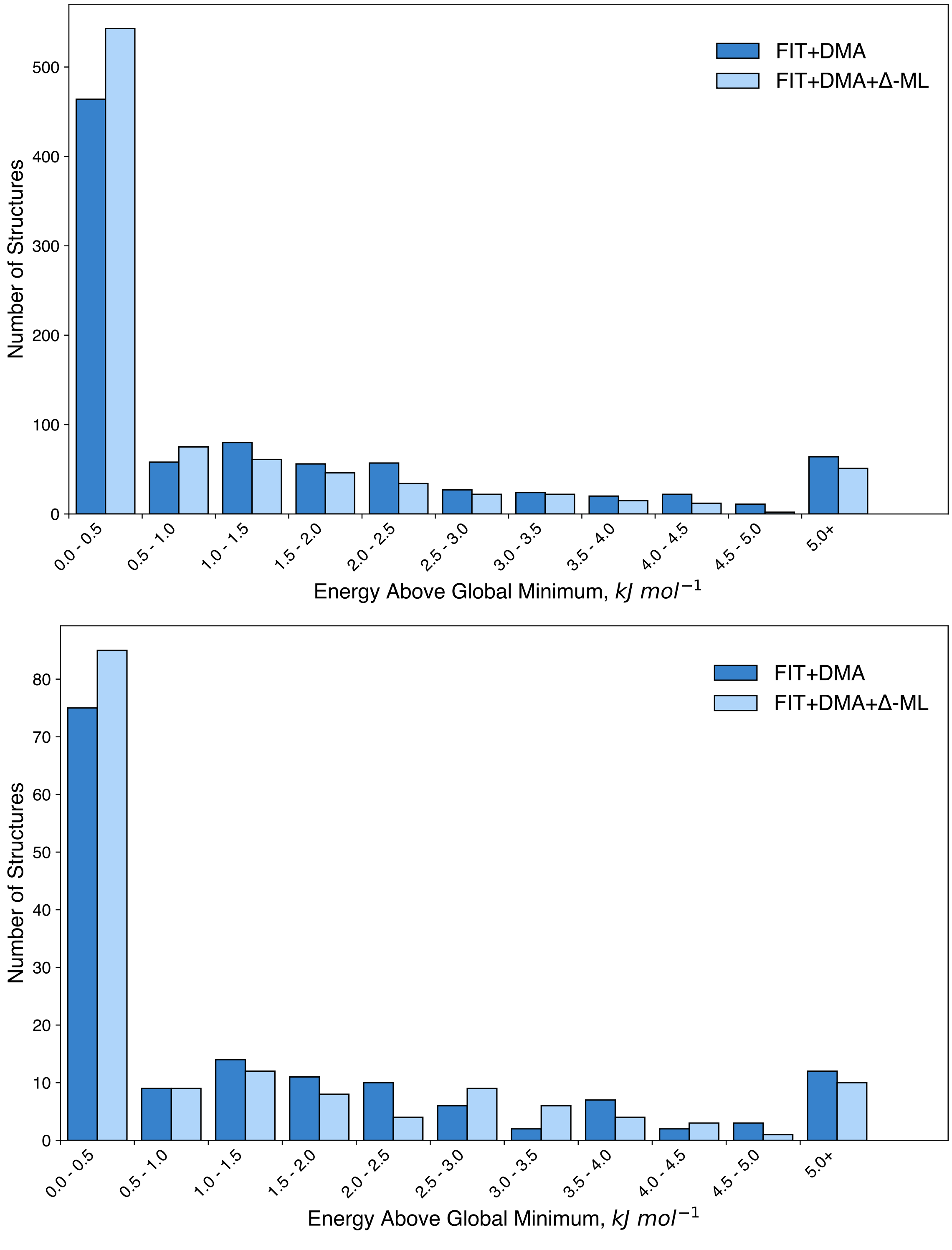

Figure S5: Distribution of the experimental structures with the two energy models in terms of energies above the global minimum for the training compounds (above) and extrapolation compounds (below).

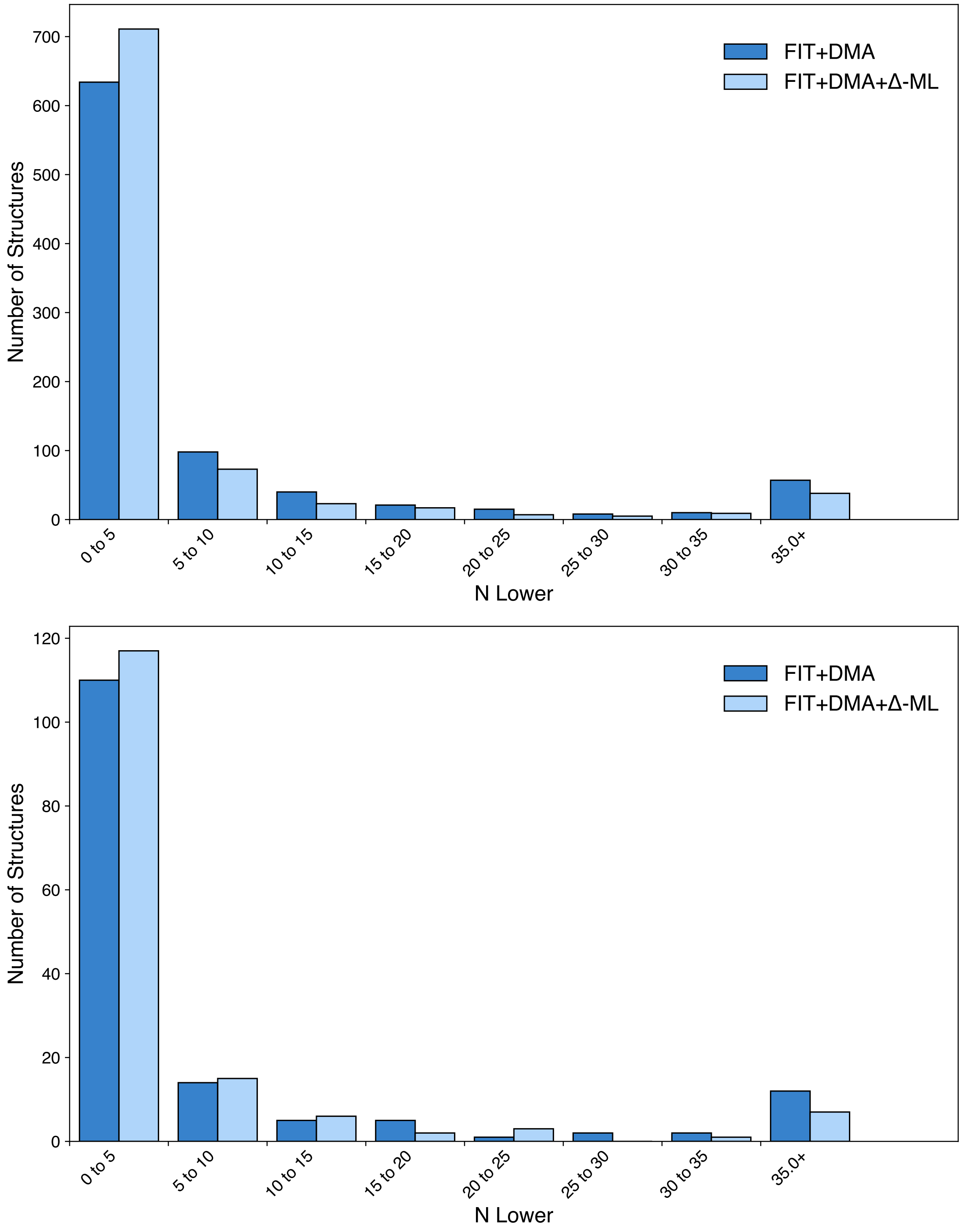

Figure S6: Distribution of the experimental structures with the two energy models in terms of the number of lower ranked structures (N lower) for the training compounds (above) and extrapolation compounds (below).

S3 Molecular Data

Table S3 (presented at end of document for legibility) is the complete list of CSD refcodes for which matches were sought on our CSP energy landscapes, totalling 1041 distinct crystal structures of 1007 unique molecular species. All entries have a SMILES string (generated using the CSD's ConQuest

software) except for 3 systems containing delocalised bonds for which a conventional SMILES string could not be produced: BDEHAN10, DONTAB, and KOYLIT. The 6 entries which were not located on our CSP landscapes (i.e. for which no match to experiment was found) are denoted with energy ranks, $RMSD_{30}$ values, and numerical ranks of "None".

This data is also available in a structured, machine-readable format (CSV file) alongside the optimised crystal structures (as concatenated CIF files) and lattice energy rankings for each landscape (CSV files) from DOI: 10.5258/SOTON/D3094.

References

(1) Case, D. H.; Campbell, J. E.; Bygrave, P. J.; Day, G. M. *Journal of chemical theory and computation* **2016**, *12*, 910–924.

(2) Coombes, D. S.; Price, S. L.; Willock, D. J.; Leslie, M. *The Journal of Physical Chemistry* **1996**, *100*, 7352–7360.

(3) Price, S. L.; Leslie, M.; Welch, G. W. A.; Habgood, M.; Price, L. S.; Karamertzanis, P. G.; Day, G. M. *Phys. Chem. Chem. Phys.* **2010**, *12*, 8478–8490.

(4) Williams, D. E.; Houpt, D. J. *Acta Crystallographica Section B* **1986**, *42*, 286–295.

(5) Holden, J. R.; Du, Z.; Ammon, H. L. *Journal of Computational Chemistry* **1993**, *14*, _eprint: https://onlinelibrary.wiley.com/doi/pdf/10.1002/jcc.540140406, 422–437.

(6) Spek, A. L. *Acta Cryst. Sect. D* **2009**, *65*, 148–155.

(7) Cui, P.; McMahon, D. P.; Spackman, P. R.; Alston, B. M.; Little, M. A.; Day, G. M.; Cooper, A. I. *Chemical Science* **2019**, *10*, Publisher: The Royal Society of Chemistry, 9988–9997.

(8) Chisholm, J. A.; Motherwell, S. *J. Appl. Crystallogr.* **2005**, *38*, 228–231.

(9) Singraber, A.; Morawietz, T.; Behler, J.; Dellago, C. *Journal of Chemical Theory and Computation* **2019**, *15*, 3075–3092.

(10) Singraber, A.; Behler, J.; Dellago, C. *Journal of Chemical Theory and Computation* **2019**, *15*, 1827–1840.

(11) Behler, J. *The Journal of Chemical Physics* **2011**, *134*, 074106.

(12) Gastegger, M.; Schwiedrzik, L.; Bittermann, M.; Berzsenyi, F.; Marquetand, P. *The Journal of Chemical Physics* **2018**, *148*, 241709.

(13) Batatia, I.; Kovacs, D. P.; Simm, G.; Ortner, C.; Csanyi, G. *Advances in Neural Information Processing Systems* **2022**, *35*, 11423–11436.

(14) Larsen, A. H.; Mortensen, J. J.; Blomqvist, J.; Castelli, I. E.; Christensen, R.; Dułak, M.; Friis, J.; Groves, M. N.; Hammer, B.; Hargus, C., et al. *Journal of Physics: Condensed Matter* **2017**, *29*, 273002.

(15) Blum, V.; Gehrke, R.; Hanke, F.; Havu, P.; Havu, V.; Ren, X.; Reuter, K.; Scheffler, M. *Computer Physics Communications* **2009**, *180*, 2175–2196.

(16) Havu, V.; Blum, V.; Havu, P.; Scheffler, M. *Journal of Computational Physics* **2009**, *228*, 8367–8379.

(17) Marek, A.; Blum, V.; Johanni, R.; Havu, V.; Lang, B.; Auckenthaler, T.; Heinecke, A.; Bungartz, H.-J.; Lederer, H. *Journal of Physics: Condensed Matter* **2014**, *26*, 213201.

(18) Grimme, S.; Ehrlich, S.; Goerigk, L. *Journal of computational chemistry* **2011**, *32*, 1456–1465.

(19) Kresse, G.; Hafner, J. *Physical review B* **1993**, *47*, 558.

(20) Kresse, G.; Furthmüller, J. *Computational materials science* **1996**, *6*, 15–50.

(21) Kresse, G.; Furthmüller, J. *Physical review B* **1996**, *54*, 11169.

(22) Kresse, G.; Joubert, D. *Physical review b* **1999**, *59*, 1758.

Table S3: The complete list of all crystal structures of molecules for which our CSP workflow was attempted. The numerical ranking indicates the number of predicted structures lower in energy than the experimental match to this refcode, i.e. 0 indicates the match is the global minimum on the energy landscape.

CSD Refcode	Formula	Space Group symbol	Space Group number	SMILES	Energy rank ΔE(CSP) / kJ/mol	$RMSD_{30}$ of CSP match / Å	Numerical rank
ABUDAD	C13 H23 N1 O1	P21/c	14	O=C1NC2CCCCCCCCCCC12	0.04	0.129	1
ABUDAD01	C13 H23 N1 O1	P21/c	14	O=C1NC2CCCCCCCCCCC12	0.00	0.216	0
ACNAQU01	C12 H6 O2	P212121	19	O=C1C(=O)c2cccc3cccc1c23	0.00	0.211	0
ACRDIN04	C13 H9 N1	P21/n	14	c1ccc2nc3ccccc3cc2c1	0.25	0.541	1
ACRDIN12	C13 H9 N1	P21/n	14	c1ccc2nc3ccccc3cc2c1	0.00	0.144	0
ADAMAN02	C10 H16	P-421c	114	C1C2CC3CC1CC(C2)C3	0.00	0.234	0
ADAREE	C6 H6 F6	Pna21	33	FC1C(F)C(F)C(F)C(F)C1F	0.00	0.285	0
ADMNTB03	C10 H14 O1	Cmc21	36	O=C1C2CC3CC(C2)CC1C3	0.00	0.195	0
ADPRLA	C8 H7 N5 O1	Pbca	61	O=C1CCN2C=Nc3ncnc(N1)c23	1.30	0.217	2
AFUNEV	C10 H11 N1 O3	P21/c	14	O=C1CC2CCC1C1C2C(=O)NC1=O	0.17	0.363	1
ALOPUR	C5 H4 N4 O1	P21/c	14	O=C1NC=NC2=C1C=NN2	0.12	0.210	1
ANNULE01	C16 H16	P21/c	14	C1=CC=CC=CC=CC=CC=CC=CC=C1	0.70	0.313	2
ANONIN11	C10 H8 O4	Pbca	61	O=C1OC2(CCC32OC(=O)C=C3)C=C1	0.00	0.249	0
ANTCEN	C14 H10	P21/a	14	c1ccc2cc3ccccc3cc2c1	0.00	0.167	0
ANTQUO13	C14 H8 O2	P21/c	14	O=C1c2ccccc2C(=O)c2ccccc12	0.06	0.289	1
APUHOJ	C10 H14 N2 O2	P212121	19	O=C1C2CCCN2C(=O)C2CCCN12	0.00	0.440	0
APYFEB01	C4 H8 N2 O1	Pnma	62	O=C1NCCCN1	0.00	0.189	0
ATCDEO	C10 H12 O2	Pbca	61	O=C1CC2C(C1)C1CC(=O)CC21	7.26	0.341	118
ATIBAH	C8 H10 N2 O3	P212121	19	O=C1CC2NC(=O)N3CCC(O1)C23	1.69	0.967	1
AWUSAO	C10 H14 O4	P21/c	14	O=C1CCC2(CC3OCCCC3O2)O1	0.35	0.215	1
AWUWOE	C9 H15 N1 O1	C2/c	15	O=C1CC2(CCCCC2)CN1	0.34	0.195	1
AXALER	C10 H9 N1 O3	P21/n	14	O=C1CCC2N1CC1=C(OC=C1)C2=O	5.86	0.444	6
AZAKIW	C10 H9 N1 O1	Pbca	61	O=C1Nc2ccccc2C21CC2	0.51	0.247	1
AZALAO	C10 H12 O3	Pca21	29	O=C1COC2OCC3C=CCC1C23	0.40	0.247	1
AZCYHO	C6 N4 O4	P21/a	14	O=C1C(=O)C(=[N+]=[N-])C(=O)C(=O)C1=[N+]=[N-]	0.00	0.260	0
AZPACN	C12 H8 N2	P21/n	14	C1=CC2=CC=C3C=CC4=CC=C1N2N34	0.00	0.308	0
AZPNOD	C7 H10 N2 O2	P212121	19	O=C1CCC2(CCC(=O)N2)N1	7.10	0.255	10

Table S3 (continued)

CSD Refcode	Formula	Space Group symbol	Space Group number	SMILES	Energy rank ΔE(CSP) / kJ/mol	$RMSD_{30}$ of CSP match / Å	Numerical rank
AZTCDO10	C9 H13 N1 O1	P21/n	14	O=C1NC2CC3CCC2CC13	0.00	0.201	0
AZURAC01	C3 H3 N3 O2	P212121	19	O=C1NN=CC(=O)N1	0.48	0.156	5
BACKEY	C4 H4 N2	Pmnn	58	c1cnccn1	0.00	0.255	0
BAGKEB	C9 H8 O1	P21/c	14	C1C2OC2c2ccccc12	0.00	0.152	0
BAPFEF	C6 H7 F1 O3	P212121	19	FC1OC2COC1C1OC21	0.83	0.264	2
BAPFIJ	C6 H7 F1 O3	P212121	19	FC1OC2COC1C1OC21	3.54	0.283	6
BAPFOP	C6 H7 F1 O3	P21	4	FC1OC2COC1C1OC21	0.00	0.196	0
BAPFUV	C6 H7 F1 O3	P212121	19	FC1OCC2OC1C1OC21	0.91	0.228	2
BAPGAC	C6 H7 F1 O3	P212121	19	FC1C2COC(O2)C2OC12	0.89	0.230	2
BAPGEG	C6 H7 F1 O3	P212121	19	FC1C2COC(O2)C2OC12	0.00	0.217	0
BAPGIK	C6 H7 F1 O3	P1	1	FC1C2OC1C1OCC2O1	0.97	0.231	3
BAPGOQ	C6 H7 F1 O3	P212121	19	FC1OC2OCC1C1OC21	0.00	0.181	0
BARBAC01	C4 H4 N2 O3	P21/c	14	O=C1CC(=O)NC(=O)N1	3.50	0.313	17
BASCON	C16 H12	P21/n	14	c1ccc2C=Cc3ccccc3C=Cc2c1	0.96	0.273	1
BASDOO	C12 H16 O3	P21/n	14	O=C1CCCCC(O1)=C1CCCCC1=O	0.00	0.174	0
BATVOH	C10 H20	P21/n	14	C1CCCCCCCCC1	0.03	0.223	1
BAWVUQ01	C6 H12 N4 O4	P-1	2	C1OOCN2CN1N1COOCN2C1	1.73	0.593	1
BAYHOB	C12 H7 N1 O2	P21/c	14	O=C1NC(=O)c2c1ccc1ccccc21	1.42	0.222	2
BAYROI	C10 H10 O2	P21/n	14	O=C1OCCC21C=CC=CC=C2	0.00	0.117	0
BAZOCT	C10 H12 N2	P21/n	14	C1CN2CC1Nc1ccccc21	0.26	0.177	1
BAZYAC01	C10 H9 N1 O4	P21/c	14	N1C23C4OC4C4OC4C12C1OC1C1OC31	0.00	0.492	0
BCHXEN01	C12 H20	P-1	2	C1CCC(CC1)=C1CCCCC1	0.36	0.257	2
BCOCHY	C10 H14 N2 O2	C2/m	12	O=C1NC(=O)C2(CC3CCCC3C2)N1	1.15	0.312	2
BCODYN02	C16 H8	P21/n	14	c1ccc2C#Cc3ccccc3C#Cc2c1	0.00	0.574	0
BCYBUE01	C10 H10	P-1	2	C1Cc2cc3CCc3cc12	0.00	0.275	0
BDEHAN10	C14 H10	P21/c	14		0.00	0.157	0
BEBXOV	C9 H6 N4	P21/n	14	C1=CC=C2N3N=CN=C3N=C2C=C1	1.57	0.452	2
BEJPEL	C15 H13 N1	C2/c	15	C1C2=CC3=CC=CC=C(N3)C=C1C=CC=C2	0.00	0.319	0
BEKLAE	C8 H12 N2 O2	P21/n	14	[O]N1=C2CCCCCCC2=NO1	0.00	0.091	0

Table S3 (continued)

CSD Refcode	Formula	Space Group symbol	Space Group number	SMILES	Energy rank ΔE(CSP) / kJ/mol	$RMSD_{30}$ of CSP match / Å	Numerical rank
BEKMIN	C10 H8 N2 O2	P-1	2	O=C1ON=C2CCc3ccccc3N12	0.14	0.952	1
BEMYEY	C8 H10 N2 O2	P212121	19	O=C1NC2CC=CCC1NC2=O	0.00	0.549	0
BENZEN	C6 H6	Pbca	61	c1ccccc1	0.00	0.295	0
BENZEN03	C6 H6	P21/c	14	c1ccccc1	0.49	0.416	4
BEOXAZ01	C6 H4 N2 O1	Pc	7	O1N=C2C=CC=CC2=N1	2.61	0.313	30
BESSUN	C11 H12 O1	P212121	19	O=C1C2=CC=CC=C1CCCC2	2.66	0.213	2
BEVCEK	C10 H12 O4	P32	145	O=C1OC2CC3COC4OCC1C2C34	4.17	0.199	5
BEXGAM	C7 H8 O3	P21/a	14	C1C2OC2C2OC2C2OC12	0.00	0.164	0
BEZWIM	C8 H10 O3	P212121	19	O=C1OC(=O)C2CCCCC12	4.36	0.715	12
BEZXUZ	C13 H16 O2	P212121	19	O=C1C2CC3CCC41CCC1CC1C34O2	0.00	0.149	0
BIFXOE	C11 H14 O3	P21	4	O=C1CC2CCC34OC3CCC4C2O1	1.12	0.376	1
BIGWUJ	C12 H14 O1	P21/m	11	O=C1CC23CC=CCC12CC=CC3	0.86	0.196	1
BILLEP	C11 H12 N2 O2	P21/c	14	O=C1C2C3OC(C=C3)C2C2=NCCCN12	0.15	0.228	1
BILZUR	C9 H12 O2	P21/n	14	O=C1CCCC2C3COC12C3	1.88	0.606	5
BINDEI	C12 H6 O4	Cmca	64	O=C1CC(=O)c2cc3C(=O)CC(=O)c3cc12	0.00	0.300	0
BIRTUR	C9 H12 O3	P212121	19	O=C1CC2(CCCC2)CC(=O)O1	6.06	0.396	88
BISLOE	C7 H6 O3	P21/c	14	O=C1OC2C3OC3C3C2C13	0.32	0.210	1
BIVZIP01	C16 H8	P21/c	14	C1=CC2=C3C=C3C3=CC=CC3=C3C=C3C2=C1	1.38	1.343	1
BIXKUP	C12 H12 O3	P21/n	14	O=C1c2ccccc2CC21OCCCO2	16.32	0.813	299
BIXLOK	C11 H10 O3	P21/n	14	O=C1Cc2ccccc2C21OCCO2	4.95	1.048	29
BIZWIS	C11 H10 N2 O2	P21/n	14	O=C1C(Oc2ccccc12)=C1NCCN1	7.30	0.429	17
BMEANN	C16 H14	C2/c	15	C1C2=CC=CC=C1C=C1CC(=C2)C=CC=C1	0.48	0.239	2
BNORSO10	C14 H12 O2	P21/c	14	O=C1C2C3C2C2C4C5C6C(C3C12)C4C(=O)C56	2.11	0.172	4
BNZQUI	C6 H4 O2	P21/a	14	O=C1C=CC(=O)C=C1	3.24	0.226	23
BNZQUI02	C6 H4 O2	P21/a	14	O=C1C=CC(=O)C=C1	3.24	0.271	23
BODCOM	C10 H4 O4	P21/n	14	O=C1C=CC(=O)C2=C1C(=O)C=CC2=O	4.63	0.246	4
BOFWEZ	C11 H15 N1 O2	P212121	19	C=C1CCC2CC1C1COC(=O)N1C2	2.66	0.224	3
BOMJIW	C6 H8 F4	Pnnm	58	FC1(F)CCC(F)(F)CC1	0.00	0.180	0
BOPXOT	C10 H8 O4	P21/c	14	O1C2C3OC3C3=C(C12)C1OC1C1OC31	2.68	0.245	2

Table S3 (continued)

CSD Refcode	Formula	Space Group symbol	Space Group number	SMILES	Energy rank ΔE(CSP) / kJ/mol	$RMSD_{30}$ of CSP match / Å	Numerical rank
BOQQUT	C8 H11 N1 O2	P21/a	14	O=C1NC(=O)C2CCCC1C2	0.88	0.163	3
BOQQUT03	C8 H11 N1 O2	P21/c	14	O=C1NC(=O)C2CCCC1C2	0.88	0.345	3
BOSLAX	C9 H9 N1 O1	P212121	19	O=C1CCc2ccccc2N1	0.00	0.388	0
BOSNIG	C13 H14 O3	Pbca	61	O=C1OC2C3CC4(C5CCC(C5)C4=C3)C2O1	0.00	0.108	0
BOXAZD	C8 H5 N1 O3	P21/a	14	O=C1NC(=O)c2ccccc2O1	0.00	0.643	0
BOXBEV	C10 H10 O2	P21/n	14	O=C1CC2C3CC1C(C=C3)C2=O	0.13	0.230	1
BOXGEA	C5 H7 N3	P212121	19	C1CN2N=CC=C2N1	0.00	0.247	0
BUFDOV	C15 H12 O1	P-1	2	O=C1c2ccccc2CCc2ccccc12	0.00	0.264	0
BUGMIZ	C12 H20 N2 O1	P21/c	14	O=C1C2CCCCC2NC2CCCCN12	0.22	0.239	2
BUGTOM	C10 H11 N3 O1	P21/n	14	C1NC=NN2C1COc1ccccc21	6.99	0.331	54
BUHMOH	C16 H16	P212121	19	C1=CC2C(C=C1)C1C2C2C=CC3C(C=C2)C13	0.00	0.271	0
BUKLOK	C12 H14 O3	P21/c	14	O=C1OC2CCCCC34OC3CC=C1C24	0.00	0.259	0
BUKTAC	C16 H14	Pna21	33	C1=CC=C2C=CC3=CC=CC=CC3C2C=C1	3.12	0.208	30
BUKYUB	C15 H13 N1	P21/c	14	C1C2=CC=CC=C1C=C1CC(=C2)C=CC=N1	0.18	0.153	1
BUNSIM11	C6 H12 O1	C2/c	15	C1CCCOCC1	0.00	0.206	0
BUSFUQ	C2 H4 N4	Pca21	29	N1C=NNC=N1	4.67	0.680	3
BUTFUT	C9 H12 O4	P212121	19	O=C1CC2C3CCCOC3OC2O1	0.70	0.440	2
BUTGAA	C8 H10 O4	P21	4	O=C1CC2C3CCOC3OC2O1	0.00	0.218	0
BUVBUR	C7 H5 N1 O1	C2/c	15	O=C1C=CC2=CC=CN12	1.06	0.320	11
BUVCIF	C7 H7 N1 O1	P21/a	14	O=C1CCC2=CC=CN12	1.41	0.270	3
BUVGAC	C10 H8 N2 O2	P21	4	O=C1NC(=O)C2C1Nc1ccccc21	None	None	None
BUXQAO	C14 H16 O1	P21/n	14	C1CCC2C(C3OC3C1)c1ccccc21	2.59	0.451	10
BUYYUP	C12 H10	P21/n	14	C1=CC=Cc2ccccc2C=C1	0.00	0.302	0
BZAZPO	C14 H11 N1	Pnma	62	N1c2ccccc2C=Cc2ccccc12	0.00	0.201	0
BZCBUO	C8 H4 O2	Pbc21	29	O=C1C(=O)c2ccccc12	0.00	0.134	0
BZCOCT	C16 H12	P21/c	14	c1ccc2C3C(C4C3c3ccccc43)c2c1	4.36	0.235	34
BZCYDY01	C14 H12	P41212	92	C1CCC#Cc2ccccc2C#CC1	0.00	0.166	0
BZDIOX02	C12 H8 O2	C2/c	15	O1c2ccccc2Oc2ccccc12	0.08	0.093	1
BZDMAZ	C7 H6 N2	P21nb	33	N1C=Nc2ccccc12	6.87	0.317	42

Table S3 (continued)

CSD Refcode	Formula	Space Group symbol	Space Group number	SMILES	Energy rank ΔE(CSP) / kJ/mol	$RMSD_{30}$ of CSP match / Å	Numerical rank
BZDMAZ03	C7 H6 N2	Pccn	56	N1C=Nc2ccccc12	1.93	0.167	5
BZOXAZ	C8 H5 N1 O3	P21/a	14	O=C1Nc2ccccc2C(=O)O1	0.00	0.239	0
BZOXZO01	C7 H5 N1 O2	P212121	19	O=C1Nc2ccccc2O1	3.69	1.129	6
BZTROP11	C11 H8 O1	P21/n	14	O=C1C=Cc2ccccc2C=C1	1.44	0.363	1
BZTRZO	C7 H5 N3 O1	P212121	19	O=C1NN=Nc2ccccc12	4.93	0.180	20
CACRED	C13 H10 F2	P212121	19	FC1(F)C2=CC=CC=CC1=CC=CC=C2	3.55	0.487	39
CAGKUQ	C16 H12	Pbca	61	C1=Cc2ccccc2c2ccccc2C=C1	0.17	0.135	1
CAJDIC	C10 H10 O2	P212121	19	O=C1C=CC2CC3=CCCC13O2	0.00	0.193	0
CAMWUI	C11 H12 O4	P41	76	O=C1C2OCC(O2)C2C3CC(C4OC34)C12	1.21	0.264	2
CANPAL	C12 H7 F2 N1 O1	P21/n	14	FC1(F)C(=O)Nc2ccc3ccccc3c12	5.97	0.409	50
CAPRYL	C8 H15 N1 O1	Cc	9	O=C1CCCCCCCN1	0.00	0.216	0
CARPOA	C13 H10 O1	P212121	19	O=C1C=CC=Cc2ccccc2C=C1	2.29	0.347	2
CARYUQ	C9 H6 F1 N1 O1	P21/c	14	Fc1ccc2C(=O)C=CNc2c1	0.00	0.250	0
CAWDUZ	C14 H10 O2	Pbca	61	O1C2C=CC1c1cc3C4OC(C=C4)c3cc21	0.00	0.233	0
CAWDUZ01	C14 H10 O2	P21/c	14	O1C2C=CC1c1cc3C4OC(C=C4)c3cc21	0.20	0.899	1
CAXNUL06	C10 H4 F4	P21/n	14	Fc1c(F)c(F)c2ccccc2c1F	1.25	0.318	1
CAXPAT	C9 H4 F3 N1	Pc	7	Fc1nc(F)c2ccccc2c1F	3.81	0.160	11
CAXPUN	C9 H5 F1 O2	Pc	7	Fc1cccc2C=CC(=O)Oc12	2.36	0.899	16
CAXQAU	C9 H4 F2 O2	P21/c	14	Fc1ccc2OC(=O)C=Cc2c1F	0.00	0.140	0
CAXQUO	C9 H4 F2 O3	P21/m	11	FC1(F)C(=O)C=CC2=C1OC(=O)C=C2	1.72	0.166	4
CAZPUQ	C5 H8 N2 O2	P21/c	14	O=C1CCNC(=O)CN1	0.06	0.486	1
CBFBZF	C14 H10 O2	P212121	19	O1C2Oc3ccccc3C2c2ccccc12	7.74	0.532	153
CBFBZF01	C14 H10 O2	Cc	9	O1C2Oc3ccccc3C2c2ccccc12	1.11	0.184	1
CDBALA	C6 H10 N2 O2	C2/c	15	O=C1CCNC(=O)CCN1	5.15	0.365	11
CDECDO10	C10 H10 O2	P-1	2	O=C1CC=CCC(=O)C=CC=C1	0.00	0.287	0
CECGEX	C10 H8 O2	P21	4	O=C1CCC(=O)c2ccccc12	0.00	0.346	0
CEDDUK01	C10 H16 O2	P21/n	14	O=C1CCCCC(=O)CCCC1	0.00	0.154	0
CEDSAH	C9 H14 N2 O1	P21/c	14	[O-]C1=C2CCCCCCC2=[NH+]N1	0.00	0.527	0
CEHZUK	C14 H12 O1	P21/n	14	C1C2OC(=Cc3ccccc13)C=CC=C2	0.18	0.845	1

Table S3 (continued)

CSD Refcode	Formula	Space Group symbol	Space Group number	SMILES	Energy rank ΔE(CSP) / kJ/mol	$RMSD_{30}$ of CSP match / Å	Numerical rank
CEKWEU	C14 H18	P21/c	14	C1CCc2cc3CCCCc3cc2C1	0.00	0.495	0
CELKOT	C11 H9 N1	P-1	2	C1Cc2nc3ccccc3cc12	0.00	0.230	0
CEMMIT	C11 H9 N1	P21/n	14	C1N2C=CC=C2c2ccccc12	0.00	0.254	0
CENRUJ	C9 H8 N2 O2	P21/m	11	O=C1CC(=O)Nc2ccccc2N1	0.00	0.359	0
CEQROI	C9 H7 N1 O2	P212121	19	O=C1Nc2ccccc2C21CO2	0.00	0.152	0
CEYJUN	C10 H8 N2 O2	P21/c	14	[O]N1=C2CCc3ccccc3C2=NO1	0.00	0.301	0
CICSUD	C12 H7 N1 O2	P21/n	14	O=C1NC(=O)c2cc3ccccc3cc12	0.47	0.489	2
CICWEQ	C14 H10 O2	P21/a	14	O1OC2c3ccccc3C1c1ccccc21	0.34	0.163	1
CIFSOB	C11 H14 O2	P21/c	14	O=C1CCC2CC3CCC(=O)C3C12	0.65	0.266	1
CIFSUH	C11 H10 O2	P21/n	14	O=C1C=CC2CC3C=CC(=O)C3C12	1.97	0.547	4
CIGHEF	C15 H14 O1	Pna21	33	C1CC2CC1C1=C2C2OC1c1ccccc21	0.00	0.156	0
CIGKAE	C12 H16 O3	P21	4	O=C1CCC23CCCCC2(C1)OC(=O)C3	0.00	0.171	0
CIJGOR	C10 H14 O2	P21/a	14	O=C1CCC(=O)C2CCCCC12	3.69	0.388	7
CILWUP11	C3 H3 N3	P-1	2	c1cnnnc1	0.49	0.557	2
CIMHUB	C14 H24	P-1	2	C1CCC2CC3CCCCC3CC2C1	0.15	0.268	1
CIMNUH	C5 H2 O3	P41212	92	O=C1C=CC(=O)C1=O	0.00	0.265	0
CIMYUS	C12 H16	C2/c	15	C1CC2=C3CCC(=C1CC2)CC3	0.00	0.137	0
CIPMAP	C13 H14 O1	Pn21a	33	O=C1C2=CC=CC=C1C1CCC2CC1	0.00	0.132	0
CIQCEK	C14 H12	P21/n	14	C1CC#CC#CCCCC#CC#CC1	0.26	0.373	1
CIQKUJ	C9 H7 N1 O1	P21/n	14	O=C1NC=Cc2ccccc12	0.00	0.434	0
CIVXIO10	C3 H6 O1	Pnam	62	C1COC1	0.00	0.350	0
CLPRPR01	C10 H14 N2 O2	P212121	19	O=C1C2CCCN2C(=O)C2CCCN12	0.00	0.450	0
COJMAR	C6 H3 F1 N2 O2	P21/n	14	FC1=CC=CC2=[N+]([O-])ON=C12	2.68	0.265	9
COLDEM	C16 H10	P21/n	14	C1=CC2=CC3=C4C(=CC(=C24)C=C1)C=CC=C3	0.00	0.416	0
COMFAO	C12 H13 N1 O1	P212121	19	O=C1CCC2(CCc3ccccc23)N1	2.52	0.397	3
CONYAH	C13 H10 O1	Pnma	62	C1c2ccccc2Oc2ccccc12	0.82	0.555	1
COQVOW	C11 H8 N2	P212121	19	N1N=Cc2ccc3ccccc3c12	0.00	0.939	0
COUMAR11	C9 H6 O2	Pc21b	29	O=C1Oc2ccccc2C=C1	1.02	0.323	3
COUMAR13	C9 H6 O2	P21	4	O=C1Oc2ccccc2C=C1	1.53	0.281	7

Table S3 (continued)

CSD Refcode	Formula	Space Group symbol	Space Group number	SMILES	Energy rank ΔE(CSP) / kJ/mol	$RMSD_{30}$ of CSP match / Å	Numerical rank
COUMAR18	C9 H6 O2	P212121	19	O=C1Oc2ccccc2C=C1	3.04	0.273	23
COVGIE	C12 H12 O2	P21/n	14	C1OCC2=C1C=CC1=C(COC1)C=C2	2.06	0.305	9
CPENAD01	C5 H6	P21/c	14	C1C=CC=C1	0.87	0.369	11
CPFLUR	C15 H10	Pbcn	60	C1C=C2c3ccccc3c3cccc1c23	4.02	0.201	16
CPYANN	C17 H12	C2/c	15	C1C23C4=CC5=CC=CC=C(C=C2C=CC=C4)C135	0.00	0.135	0
CRBZOL11	C12 H9 N1	Pnma	62	N1c2ccccc2c2ccccc12	0.00	0.262	0
CUBANE01	C8 H8	R-3	148	C12C3C4C1C1C2C3C41	0.00	0.389	0
CUDJIU01	C14 H14 O1	P-421c	114	C1Cc2ccc(CCC3=CC=C1O3)cc2	0.00	0.218	0
CUGBIP	C8 H6 N2 O2	P21/c	14	O=C1C=C([O-])[NH+]=C2C=CC=CN12	0.62	0.441	1
CUKCIU	C4 H8 O2	P21/n	14	C1COCCO1	0.00	0.222	0
CUKCIU01	C4 H8 O2	P21/n	14	C1COCCO1	3.32	0.235	31
CUMJID	C8 H12 N2 O2	P21/n	14	O=C1CCC2CCNC(=O)C2N1	0.00	0.309	0
CUMJOJ	C8 H12 N2 O2	P-421c	114	O=C1CCC2CCNC(=O)C2N1	0.00	0.215	0
CURVES	C16 H11 N1	P212121	19	N1c2ccccc2c2c1ccc1ccccc21	1.71	0.494	1
CUSHIH10	C13 H10 O2	Pccn	56	O=C1CCOc2ccc3ccccc3c12	0.91	0.300	7
CUWYUP	C6 H2 F4	P21/c	14	Fc1cc(F)c(F)c(F)c1	0.98	0.282	4
CUXXAW	C14 H9 N3	P212121	19	c1ccc2nc3N4C=CC=C4C=Cc3nc2c1	0.00	0.324	0
CYCHEX	C6 H12	C2/c	15	C1CCCCC1	1.03	0.771	3
CYDECO	C10 H18 O1	P21/c	14	O=C1CCCCCCCCC1	0.05	0.259	1
CYHEXO	C6 H8 O2	P21	4	O=C1CCC(=O)CC1	0.71	0.681	6
CYTDEC	C14 H28	P-1	2	C1CCCCCCCCCCCCC1	1.60	0.166	6
CYURAC05	C3 H3 N3 O3	C2/n	15	O=C1NC(=O)NC(=O)N1	5.40	0.296	7
DABLIB	C8 H8 F4	Cmca	64	FC1(F)C2CC3C(CC12)C3(F)F	0.00	0.087	0
DABPIF	C14 H16	P21/c	14	C1C2C3C4C1C1C2C2C5CC(C32)C4C15	0.04	0.091	1
DAFJID	C11 H8 N2	P21/n	14	N1c2ccccc2c2cccnc12	0.00	0.433	0
DAGSAG	C14 H24 O1	P21	4	O=C1CCCCCCCCCC=CCC1	0.52	0.249	2
DAJXOB	C9 H8 O3	Pnma	62	O=C1OC(=O)C2=C1C1CCC2C1	0.48	0.383	3
DALBEY	C8 H10 O3	P-1	2	O=C1OCC23OCCC2CC13	0.00	0.329	0
DALBIC	C11 H14 O3	Pbca	61	O=C1OCC23OC4CCCC4C2CC13	1.45	0.343	4

Table S3 (continued)

CSD Refcode	Formula	Space Group symbol	Space Group number	SMILES	Energy rank ΔE(CSP) / kJ/mol	$RMSD_{30}$ of CSP match / Å	Numerical rank
DANJEH	C12 H12 O2	P21/c	14	O=C1C2CC3C4C2C2C1CC(C42)C3=O	0.00	0.186	0
DATJOA	C13 H13 N1 O1	P21/c	14	O=C1Cc2ccccc2C2CN1CC=C2	2.32	0.191	7
DATLER	C5 H2 F3 N1	P-1	2	Fc1cnc(F)c(F)c1	4.21	1.135	179
DATLIV01	C5 H1 F4 N1	Pnma	62	Fc1cc(F)c(F)nc1F	0.00	0.146	0
DAVLIV	C14 H20	P21/n	14	C1CCC(CC1)=C=C=C1CCCCC1	0.00	0.154	0
DAWSUP01	C6 H6 N2 O2	P21/c	14	C=C1NC(=O)C(=C)NC1=O	1.08	0.531	6
DAZCOE	C6 H10 N2	P21212	18	C1CC2CCC1N=N2	0.00	0.258	0
DAZNAP01	C8 H6 N2	Pbca	61	c1ccc2cnncc2c1	2.11	0.525	16
DAZNAQ	C8 H6 N2	P21/n	14	c1cc2ccncc2cn1	3.50	0.878	11
DAZNAR	C8 H6 N2	P41212	92	c1cnc2cccnc2c1	3.59	0.158	26
DAZPNE	C12 H8 N2	P21/c	14	c1ccc2c(c1)nnc1ccccc21	1.31	0.170	1
DAZVEF	C12 H12 O3	P21/c	14	O=C1C2(CC2)C(=O)C2(CC2)C(=O)C21CC2	3.58	0.521	13
DBOXEP11	C14 H10 O1	Pnma	62	O1c2ccccc2C=Cc2ccccc12	0.00	0.136	0
DEBFOI	C12 H11 N1 O1	P21/n	14	O=C1CCC2=Cc3ccccc3CN12	4.18	1.208	76
DEBFUO	C13 H13 N1 O1	P21/c	14	O=C1CCCC2=Cc3ccccc3CN12	2.21	0.388	5
DEPLNA	C12 H16 O3	P21/n	14	C1CCC23COCC2(C1)C1OC1C1OC31	2.73	0.245	11
DEPPRD	C12 H12 O3	P212121	19	C1OCC23C=CC=CC12C1OC1C1OC31	0.00	0.139	0
DEXTIJ	C11 H8 N2	P21/n	14	C1c2cccnc2c2ncccc12	0.28	0.273	1
DEZDUH	C8 H6 F6	Pna21	33	FC1(F)CC2C3CC(F)(F)C2(F)C13F	2.43	0.162	14
DFNAPH10	C10 H6 F2	P21/c	14	Fc1cccc2c(F)cccc12	0.00	0.209	0
DFNAPQ	C10 H4 F2 O2	P-1	2	FC1=C(F)C(=O)c2ccccc2C1=O	0.00	0.814	0
DHCBAN	C14 H10	Pnma	62	c1cc2cccc3C4C=CC4c(c1)c23	0.00	0.108	0
DHPROA	C10 H10 N2 O2	P41212	92	O=C1C2C=CCN2C(=O)C2C=CCN12	0.00	0.461	0
DHURAC10	C4 H6 N2 O2	P21/c	14	O=C1CCNC(=O)N1	0.00	0.139	0
DIAZNP	C8 H6 N2	P21/c	14	c1cc2cnccc2cn1	1.46	0.531	1
DIHIXL10	C4 H4 N2 O2	P21/n	14	O1N=CC2ON=CC12	0.00	0.175	0
DIHWUM	C12 H16 O3	Pn	7	O=C1C2CCC=CC2OC21CCCCO2	1.94	0.292	2
DIJDAE	C12 H10 O3	P21/c	14	O=C1COc2ccccc2C2=C1CCO2	2.21	0.273	2
DIKETE11	C4 H4 O2	P21/c	14	C=C1CC(=O)O1	2.04	0.207	10

Table S3 (continued)

CSD Refcode	Formula	Space Group symbol	Space Group number	SMILES	Energy rank ΔE(CSP) / kJ/mol	$RMSD_{30}$ of CSP match / Å	Numerical rank
DIKPIP03	C4 H6 N2 O2	P21/c	14	O=C1CNC(=O)CN1	0.00	0.156	0
DILCIK	C12 H16	P21/n	14	C1CC2CC1C1=C2C2CCC1C2	0.00	0.217	0
DISBUE	C6 H7 N1 O3	P21/c	14	O=C1CCNC(=O)C2OC12	2.92	0.419	5
DITBOX	C14 H12	P21	4	C1c2ccccc2Cc2ccccc12	0.00	0.227	0
DIUREA04	C4 H6 N4 O2	Cmcm	63	O=C1NC2NC(=O)NC2N1	0.00	0.172	0
DIXNUT	C7 H11 N1 O2	P21/c	14	O=C1NCC2CCCC2O1	3.70	0.210	12
DNNEPH10	C10 H6 N2 O2	P1121/b	14	O=N1c2cccc3cccc(c23)N1=O	None	None	None
DOBYOJ	C7 H9 N1 O3	P21/c	14	O=C1OCCC21CCC=[N+]2[O-]	1.27	0.654	12
DOGPAQ	C6 H8 N2 O2	Pn	7	O=C1NCC2C1CNC2=O	0.00	0.203	0
DOGTIC	C7 H10 N2 O2	P212121	19	O=C1NCC2CC1CNC2=O	0.00	0.127	0
DOJTUR	C8 H11 N1 O2	P21/n	14	O=C1NC2CC=CCC2CO1	0.63	0.234	2
DONTAB	C7 H10 N2 O2	P21/n	14		0.00	0.266	0
DOTRAG	C11 H8 O4	P21/c	14	O=C1C(=O)C2(OCCO2)c2ccccc12	3.68	0.485	3
DSZNDO10	C7 H10 N2 O2	C2/c	15	O=C1NCCC21CCNC2=O	0.00	0.152	0
DUBGUC	C9 H10 O2	Pnma	62	C1OCc2ccccc2CO1	1.62	0.157	5
DUBRAT	C10 H12 N2 O2	P212121	19	O=C1CCC(=O)N2C3CCC(C=C3)N12	0.00	0.097	0
DUBREX	C8 H12 N2 O2	C2/c	15	O=C1CCC(=O)N2CCCCN12	0.00	0.181	0
DUFBOV11	C2 H4 O1	P21/n	14	C1CO1	0.71	0.197	6
DUKVOU	C13 H10 O2	C2/c	15	O=C1CCOc2c1ccc1ccccc21	1.60	0.173	4
DUNVEN	C3 H4 N2 O2	P21/c	14	O=C1CC(=O)NN1	5.63	0.719	93
DUTTAN10	C10 H6 O3	P21	4	O=C1C2OC2C(=O)c2ccccc12	0.00	0.270	0
DUXYUQ	C6 H4 N2 O4	Cmca	64	O=C1CC(=O)N2N1C(=O)CC2=O	4.05	0.819	10
DZBASK	C8 H8 N2	P21/n	14	N1=NC2C3C4C1C1C2C3C41	1.07	0.402	7
DZCDON	C8 H14 N2 O2	Pn	7	O=C1CCCC(=O)NCCCN1	0.61	1.240	5
DZHPDO	C5 H8 N2 O2	P21/c	14	[O]N1=N([O])C2CCC1C2	0.92	0.309	3
DZOCTO	C6 H10 N2 O1	P21/n	14	[O-][N+]1=NC2CCC1CC2	2.87	0.462	41
DZSNON	C7 H10 N2 O2	P21/c	14	O=C1CCC2(CCNC2=O)N1	0.92	0.144	2
DZTCDE	C8 H12 N2	P21/m	11	C1CC2N=NC1C1CCC21	1.90	0.207	1
DZXTUN	C9 H12 N2 O2	C2/c	15	O=C1CCC23CCC(=O)N2CCN13	2.35	0.200	6

Table S3 (continued)

CSD Refcode	Formula	Space Group symbol	Space Group number	SMILES	Energy rank ΔE(CSP) / kJ/mol	$RMSD_{30}$ of CSP match / Å	Numerical rank
EBAXIQ	C13 H15 N1 O1	Pbcn	60	O=C1Nc2ccccc2C21CCCCC2	1.87	0.185	5
EFUMAU	C4 H9 N1	P21/c	14	C1CCNC1	1.49	0.266	12
EGOTEA	C13 H18 O1	P212121	19	C=C1CC23CCC1CC2CC(=O)CC3	2.64	0.255	3
EGOZUX	C13 H15 N1 O1	C2/c	15	O=C1CCC2CCc3ccccc3CN12	3.52	0.337	4
EHOPAU	C5 H6 F4	P21/c	14	FC1CC(F)C(F)C1F	5.22	0.458	31
EKUDUK	C11 H6 O3	C2/c	15	O=C1OC=CC2=C1c1ccccc1O2	2.87	0.154	8
ELUKIH	C5 H5 N7	Pn	7	C1NC2=NC=NN2C2=NNN=C12	2.47	0.581	15
ENANOL	C7 H13 N1 O1	C2/c	15	O=C1CCCCCCN1	1.20	0.376	1
ENIDAH	C7 H10 N2 O2	P-1	2	O=C1NC(=O)C2(CCCC2)N1	0.00	0.277	0
ENXBCO01	C7 H8 O3	P21/c	14	O=C1CC2C(CC3OC23)O1	0.00	0.254	0
EPXCND	C9 H12 O3	Pnma	62	C1C2OC2CC2OC2CC2OC12	0.00	0.138	0
EQIXEI	C9 H6 N4 O1	P21/c	14	O=C1NN=CN2c3ccccc3N=C12	2.26	0.222	2
EQOLUR	C12 H14 O2	P-1	2	C=C1C2CC3OC(=O)C42CCCCC134	0.00	0.114	0
ERITII	C8 H13 N1 O1	P212121	19	O=C1NC2CCCCCC12	0.00	0.053	0
EROGAU	C9 H9 N1 O1	P212121	19	O=C1CCCc2cccnc12	2.21	0.175	2
ETAJUE	C11 H10 O3	P21/n	14	O=C1OC2Cc3ccccc3CC2O1	0.00	0.243	0
ETHLEN10	C2 H4	P21/n	14	C=C	0.11	0.254	5
ETOFUQ	C11 H12 O2	C2/m	12	C1C2C=CC1C1OC3C=CC3OC21	0.00	0.199	0
EVAWEE	C11 H6 N2 O2	P21/c	14	O=C1N2C=CN=C2C(=O)c2ccccc12	0.00	0.182	0
EVOPOV	C10 H6 N2 O1	Pbca	61	O=C1C=Cc2ccccc2C1=[N+]=[N-]	1.51	0.399	3
EXEBEQ	C6 H12 N2 O1	P21/c	14	C1NCC2CNCC1O2	0.51	0.232	1
FACFAQ	C6 H5 F1	P43212	96	Fc1ccccc1	0.00	0.208	0
FACFOE	C6 H4 F2	P21/n	14	Fc1ccccc1F	2.06	0.176	52
FACGEV	C6 H4 F2	P21/c	14	Fc1ccc(F)cc1	18.88	0.508	415
FACJAU	C6 H2 F4	P21/c	14	Fc1cc(F)c(F)cc1F	1.08	1.194	13
FADMIG	C7 H6 N2 O1	P21/n	14	O=C1NNc2ccccc12	3.17	0.487	14
FADXOA	C9 H7 N1 O1	Pn	7	[O-][n+]1cccc2ccccc12	6.34	0.413	193
FAFDAS01	C7 H8 O2	P212121	19	O=C1OCC2CC32CC13	1.00	0.213	1
FAFDAT	C7 H8 O2	P21/c	14	O=C1OCC2CC32CC13	3.29	0.204	26

Table S3 (continued)

CSD Refcode	Formula	Space Group symbol	Space Group number	SMILES	Energy rank ΔE(CSP) / kJ/mol	$RMSD_{30}$ of CSP match / Å	Numerical rank
FAFDIA	C11 H14	C2	5	C1CC21CC12CC21CC12CC1	0.30	0.240	1
FAFDOG	C11 H14	C2/c	15	C1CC21CC12CC21CC12CC1	1.55	0.250	4
FAFKEC	C12 H18 N2	R-3r	148	C1CC2CCC1N1C3CCC(C=C3)N21	5.89	0.300	183
FAFKOM	C12 H20 N2	C2/c	15	C1CC2CCC1N1C3CCC(CC3)N21	0.00	0.225	0
FAHNUZ	C9 H8 O2	P21/c	14	O=C1C=CC2CC1C=CC2=O	0.94	0.652	2
FAJGUS	C7 H8	P21/c	14	C1C2C=CC1C=C2	0.00	0.198	0
FALJEI	C11 H13 N1 O3	C2	5	O=C1OC2(CC(=O)N3CCCCC23)C=C1	1.99	0.292	6
FANNUD	C6 H9 N1 O2	P212121	19	O=C1NC2CCCC1O2	6.89	0.213	13
FANNUL	C14 H14	P21/c	14	C1=CC=CC=CC=CC=CC=CC=C1	0.00	0.298	0
FARMEQ	C12 H8 N4	P21/c	14	C1=CN2C(=NC3=C2N=C2C=CC=CN32)C=C1	3.20	0.251	21
FARTID	C12 H8 O3	P21/c	14	O=C1OCOc2cc3ccccc3cc12	8.65	0.333	275
FECKIK	C9 H12 O2	Pna21	33	O=C1CC2CC=CCC2CO1	1.16	0.234	19
FEJDAA	C11 H10 N4	P21/c	14	C1N=NC2C1CN1C2=Nc2ccccc12	0.00	0.254	0
FELCIK	C6 H10 N2 O2	P212121	19	C1NOC2CONCC2=C1	0.00	0.189	0
FEPNAP	C3 H5 N1 O1	P-1	2	O=C1CCN1	0.00	0.375	0
FEZYEO	C12 H13 N1 O1	P21/n	14	O=C1CCC2CCN1c1ccccc21	1.39	0.176	6
FIBWIW	C12 H16 O2	C2cb	41	O=C1C2CCC(C2)C(=O)C2CCC1C2	2.38	0.351	1
FIBWUI	C12 H10 O2	P21/c	14	O=C1C2C=CC3C2C2C1C=CC2C3=O	0.06	0.175	1
FIGDUX	C10 H7 N3 O2	P21/c	14	O=C1NC=C2Oc3ccccc3NC2=N1	5.66	0.237	45
FIQTIK	C5 H4 N4	Cc	9	c1ccn2nnnc2c1	0.07	0.111	2
FIRLAU	C4 H3 F2 N1	Pbca	61	FC1=CNC=C1F	3.50	0.252	34
FIWYIT	C12 H6 O3	P21/c	14	O=C1OC(=O)c2c1ccc1ccccc21	1.64	0.525	2
FLNAPH	C10 H6 F2	P21/c	14	Fc1cccc2cccc(F)c12	0.00	0.130	0
FLUREN02	C13 H10	Pnma	62	C1c2ccccc2c2ccccc12	1.77	0.320	1
FMANUL01	C11 H8 F2	Pna21	33	FC1(F)C2=CC=CC=C1C=CC=C2	1.78	0.237	7
FOBSEU	C6 H8 N2 O2	P21/n	14	O=C1CCN2N1CCC2=O	1.29	0.414	3
FOCNIU	C8 H8 F4	Cmcm	63	FC1(F)C2CC3C(CC12)C3(F)F	0.00	0.180	0
FOFDOW	C13 H11 N3	P21/c	14	C1CN2c3ccccc3N=C2C2=CC=CN12	0.00	0.384	0
FOLTUX	C10 H12 O4	P212121	19	O=C1CC2OCC3CCOC2(O3)C=C1	1.89	0.208	2

Table S3 (continued)

CSD Refcode	Formula	Space Group symbol	Space Group number	SMILES	Energy rank ΔE(CSP) / kJ/mol	$RMSD_{30}$ of CSP match / Å	Numerical rank
FOLXIN	C10 H19 N3	P21/c	14	C1CN2CCCN3CCCN(C1)C23	1.57	0.436	3
FOMQIK	C9 H11 N1 O1	Pna21	33	O=C1NC2(CCC=CC2)C=C1	3.62	0.206	21
FOPCOC	C16 H16	P21/c	14	C1CC2=CC=CC=C(C2)C2=CC=CC=C1C2	2.44	0.298	6
FOPCUI	C16 H16	Pbca	61	C1CC2=CC=CC=C(C2)C2=CC=CC=C1C2	0.00	0.144	0
FOSXAM	C12 H18 N2 O1	P21/c	14	O=C1N2CCCCCCC31CCCC3=N2	1.13	0.174	3
FOWBAU	C5 H4 O3	P21/m	11	O=C1OC(=O)C2CC12	1.16	0.326	2
FPYRMO	C4 H3 F1 N2 O1	P21/c	14	FC1=CNC(=O)N=C1	1.45	0.317	14
FUBJUJ	C14 H11 N1 O1	P21/c	14	O=C1Nc2ccccc2C2=C1C1CC2C=C1	1.93	0.337	2
FUCOUM	C11 H6 O3	P21/n	14	O=C1Oc2ccc3C=COc3c2C=C1	0.00	0.307	0
FUCOUN	C11 H6 O3	Pna21	33	O=C1Oc2c(C=C1)ccc1OC=Cc21	2.30	0.219	3
FUDYOS	C6 H12 N2 O4	C2/c	15	C1CN2COOCN1COOC2	0.11	0.206	1
FUFBAJ	C8 H12 O2	P-1	2	C1OCC=CCOCC=C1	0.00	0.170	0
FULZIV	C13 H10 O2	P212121	19	O=C1OC2C3C=CC1C2c1ccccc31	0.00	0.140	0
FURACL03	C4 H3 F1 N2 O2	P21/c	14	FC1=CNC(=O)NC1=O	0.77	0.276	2
FURANE10	C4 H4 O1	P41212	92	O1C=CC=C1	6.47	0.438	198
FURCOU	C11 H6 O3	P1	1	O=C1Oc2cc3OC=Cc3cc2C=C1	0.73	0.352	2
FUROPH	C12 H12 O2	Pbca	61	C1CC2=CC=C(CCC3=CC=C1O3)O2	0.00	0.690	0
FURPOP	C13 H13 N1 O1	Cc	9	C1Cc2ccc(CCC3=CC=C1O3)nc2	0.00	0.128	0
FUTDED	C8 H6 N2 O1	Pna21	33	O=C1C=NNc2ccccc12	0.00	0.368	0
GACBUJ	C14 H16 O1	P212121	19	C=C1C=CC23CCC1CC2CC(=O)C=C3	0.00	0.213	0
GACGAU	C8 H8 O3	Pbca	61	O1C=CC2C1OC1OC=CC21	1.91	0.205	4
GACGEY	C10 H10 O4	P21/c	14	O1C=CC2C1OC1OC3OC=CC3C21	0.00	0.211	0
GAFLAB	C2 H2 F2	Pnma	62	FC=CF	0.00	0.442	0
GAFLIJ	C2 F4	P21/n	14	FC(F)=C(F)F	0.00	0.269	0
GAFLOP	C2 H1 F3	P21/n	14	FC=C(F)F	1.01	0.472	43
GAFLUV	C2 H2 F2	P21/c	14	FC=CF	0.14	0.421	1
GAHHAY	C9 H16 N2 O1	P21/c	14	O=C1NCCCN2CCCCC12	1.85	0.146	3
GAWWEH	C10 H10 O2	Pbca	61	C1Cc2cc3OCCc3cc2O1	2.10	0.116	3
GEBRAG	C11 H14 O2	P21/a	14	O=C1CCCCC23CCC(=O)C2C13	0.61	0.234	1

Table S3 (continued)

CSD Refcode	Formula	Space Group symbol	Space Group number	SMILES	Energy rank ΔE(CSP) / kJ/mol	$RMSD_{30}$ of CSP match / Å	Numerical rank
GEDXET	C3 H5 N5	P21/n	14	C1CN2N=NN=C2N1	0.00	0.125	0
GEGTAO	C12 H11 N1 O1	P21/n	14	C=C1CC2Cc3ccccc3N2C1=O	1.95	0.146	5
GEJXEY	C10 H12	Pbca	61	C1CC#CCCCC#CC1	1.65	0.076	3
GEKVEY	C9 H6 O2	Pn	7	O=C1C=CC2=COC=C2C=C1	3.05	0.423	5
GEPBOT	C11 H6 O4	P-1	2	O=C1OCC2=C1c1ccccc1C(=O)O2	1.07	0.467	1
GERZAE	C8 H9 F1 O4	P212121	19	FC1C2OCC(O2)C2CC(=O)OC12	0.00	0.181	0
GESNIB	C7 H6	P212121	19	C1c2ccccc12	0.00	0.236	0
GEYFOF	C11 H12	Iba2	45	C1CCC#CC=CC#CCC1	0.00	0.151	0
GEZKOL	C16 H16	P21/n	14	C1CC(CCC1=C1C=CC=C1)=C1C=CC=C1	0.06	0.162	1
GIGKUC	C10 H12 O4	P212121	19	O=C1CC2C1CC13OCC(COC21)O3	0.00	0.141	0
GIJBIM	C8 H10 F2 N2 O2	P21/c	14	FC1(F)CCC2(CC1)NC(=O)NC2=O	0.00	0.567	0
GIJBOS	C8 H12 F2 O2	P21/n	14	FC1(F)CCC2(CC1)OCCO2	0.00	0.208	0
GILXAD	C13 H15 N1 O1	P21/c	14	O=C1CCCC2Cc3ccccc3CN12	1.21	0.257	2
GILXIL	C13 H11 N1 O1	Pbca	61	O=C1C=CC=C2Cc3ccccc3CN12	9.26	0.386	154
GILXUX	C13 H13 N1 O1	C2/c	15	O=C1CCCC2=C1c1ccccc1CN2	1.55	0.261	4
GINPIF	C12 H12 N2	P212121	19	C1Nc2ccccc2CN2C=CC=C12	0.71	0.213	1
GIPGOE	C9 H9 N1 O3	P21/c	14	O=C1NC2CC(O1)C1=C(C2)OC=C1	0.00	0.190	0
GIPPAW	C8 H8 O4	Cc	9	O1C2C1C1OC1C1OC1C1OC21	1.48	0.300	5
GIQRAZ	C10 H8 O3	P21/c	14	O=C1OC(=O)C2=C1C1CCC2C=C1	3.82	0.207	3
GIRSAB10	C7 H10 N2 O2	P21/c	14	O=C1CC2C(CN1)CNC2=O	2.43	0.198	3
GIYHUR01	C14 H8 N2	P21/c	14	c1cc2cncc3ccc4cncc1c4c23	0.09	1.623	1
GLUTIM	C5 H7 N1 O2	P21/c	14	O=C1CCCC(=O)N1	0.00	0.170	0
GOCGEM	C10 H8 F1 N1 O3	P21/n	14	Fc1ccc2NC(=O)C3(OCCO3)c2c1	7.66	0.311	41
GOCWEA	C6 H10 O4	P21/n	14	C1OCC2OCOCC2O1	1.89	0.194	4
GOGXOP	C8 H6 F2	P-1	2	FC12C3C4C1C1C2C3C41F	0.00	0.469	0
GOHHER	C7 H6 N2 O4	Pbca	61	O=C1NC(=O)C2CC1C(=O)NC2=O	2.97	0.317	1
GOHVUU	C4 H2 N4 O4	P21/c	14	O=C1NC(=O)N2N1C(=O)NC2=O	0.18	1.455	1
GOHWAB	C8 H8	P-1	2	C1Cc2ccccc12	0.00	0.392	0
GOLHAQ	C10 H10 N4	P21/n	14	C1Cc2ccccc2N2N=NN=C2C1	0.00	0.224	0

Table S3 (continued)

CSD Refcode	Formula	Space Group symbol	Space Group number	SMILES	Energy rank ΔE(CSP) / kJ/mol	$RMSD_{30}$ of CSP match / Å	Numerical rank
GOLHIB	C11 H10 N2 O1	Cc	9	O=C1N2C(CC3CN23)c2ccccc12	0.00	0.287	0
GOLLUO01	C11 H10 O2	Pbca	61	O=C1C=CC(=O)C2C3CC(C=C3)C12	2.26	0.252	8
GURCOL	C12 H8 N2	Pca21	29	c1cnc2ccc3ncccc3c2c1	0.00	0.199	0
GUZNUL	C6 H8 F1 N1 O2	P212121	19	FC1C2COC(O2)C2NC12	0.00	0.186	0
GUZPAT	C6 H8 F1 N1 O2	P212121	19	FC1C2COC(O2)C2NC12	0.47	0.276	1
GUZPEX	C6 H8 F1 N1 O2	P212121	19	FC1C2COC(O2)C2NC12	3.56	0.288	8
GUZPIB	C6 H8 F1 N1 O2	P41	76	FC1C2COC(O2)C2NC12	0.04	0.417	1
HABYOY	C10 H12 O3	P21	4	O=C1CCC(O1)=C1CCCCC1=O	1.64	0.669	5
HACFOG	C7 H9 N1 O2	P212121	19	O=C1NC2CC=CCC2O1	3.65	0.229	3
HALKEK	C12 H12 O2	P21/c	14	O=C1CC2CCc3ccccc3C2O1	1.90	0.146	1
HAMGOT	C5 H4 N4	P21	4	C1=CN2N=CN=C2N=C1	0.98	0.219	6
HARFEO	C7 H12 F2 N2	P21/c	14	FC1(F)C2CNCC1CNC2	2.03	0.823	4
HAYXOU	C10 H10	P-1	2	C1Cc2c1ccc1CCc21	1.26	0.377	12
HAYXUA	C11 H10	P21/n	14	C1Cc2c1c1CCc1c1Cc21	0.34	1.451	8
HAYYAH	C12 H12	P21/n	14	C1Cc2c1c1CCc1c1CCc21	0.89	0.225	4
HBZIND01	C11 H7 N1 O1	P21/c	14	O=C1C=CC2=CNc3cccc1c23	0.00	0.164	0
HBZPEN	C14 H10	P21/c	14	C1c2cccc3Cc4cccc1c4c23	4.30	0.240	20
HDPDXZ	C10 H18 N2 O2	P21/c	14	C1CCN2OC3CCCCN3OC2C1	2.37	0.271	21
HEBBEV	C9 H12 O2	C2/c	15	O=C1CCC2CC1CCC2=O	0.00	0.094	0
HEGSUK	C4 H4 N2 O2	Pn	7	[O-][n+]1cccc[n+]1[O-]	0.53	0.351	3
HEKTIA	C4 H2 F2 N2 O3	P212121	19	FC1(F)C(=O)NC(=O)NC1=O	0.00	0.598	0
HEPFUL10	C14 H12	P21/c	14	C1=CC=CC(C=C1)=C1C=CC=CC=C1	0.00	0.178	0
HERMIB	C8 H6 F1 N1 O1	P21	4	Fc1ccc2NC(=O)Cc2c1	5.28	0.649	79
HETNQU01	C12 H12 O2	P212121	19	O=C1C=CC(=O)C2C3CCC(C=C3)C12	2.11	0.189	2
HEWQIK	C8 H4 N4 O2	P21/c	14	O=C1N2C=NC=C2C(=O)N2C=NC=C12	0.11	0.310	1
HEZQUY02	C8 H6 O2	P21/c	14	O=C1OCc2ccccc12	0.11	0.144	1
HIBXOF	C13 H9 N1 O1	P21/n	14	O=C1c2ccccc2Nc2ccccc12	0.00	0.291	0
HIFCIJ	C8 H10 N2	Pbca	61	C1CNc2ccccc2N1	0.00	0.265	0
HIFMUE	C12 H8 N2 O1	P212121	19	O=C1c2ccccc2Nc2ncccc12	2.35	0.871	6

Table S3 (continued)

CSD Refcode	Formula	Space Group symbol	Space Group number	SMILES	Energy rank ΔE(CSP) / kJ/mol	$RMSD_{30}$ of CSP match / Å	Numerical rank
HIKFOW	C11 H13 N1 O3	P21/c	14	O=C1OC2(CCCN3C2CCC3=O)C=C1	0.00	0.200	0
HIKJUJ	C11 H15 N1 O3	P21	4	O=C1OC23OC2CCCC23CCCCN12	1.19	0.182	1
HINPOM	C11 H22 N2 O1	Pbca	61	O=C1CCCCCCNCCCCN1	0.00	0.214	0
HIPXUZ	C8 H12	I2/a	15	C1CC21CC1(CC1)C2	0.00	0.173	0
HIQJOJ	C5 H9 N1 O1	P21/c	14	O=C1CCCCN1	1.32	0.204	4
HIRJAV	C10 H12 O4	P21/n	14	O=C1CCCC(=O)C(=O)CCCC1=O	0.00	0.100	0
HIRQOP	C12 H11 N1 O2	Pnma	62	O=C1CCN2CCC(=O)c3cccc1c23	0.00	0.260	0
HIRSUX	C7 H4 O3	C2/c	15	O=C1Oc2ccccc2O1	0.00	0.186	0
HITBOD	C10 H6 O4	P21/c	14	O=C1Cc2ccc3OC(=O)Cc3c2O1	4.43	0.361	5
HITQOT	C11 H15 N1 O2	P212121	19	O=C1NC2C3CC4CC(C3)CC2(C4)O1	0.18	0.153	1
HIXJEE	C9 H12 O3	P212121	19	O=C1OC2CCC1C1COCC21	0.00	0.229	0
HIYLIK	C14 H9 N1 O1	C2/c	15	O=C1C=CC2=Nc3ccccc3C=CC2=C1	3.73	0.809	28
HMTRAO10	C6 H12 N4 O1	R3m	160	[O-][N+]12CN3CN(CN(C3)C1)C2	0.00	0.196	0
HOBBOP	C7 H10	P21/c	14	C1CC2CC1C=C2	0.00	0.227	0
HODXII	C13 H13 N1 O1	P21/c	14	O=C1CCCCC2=C1Nc1ccccc21	0.00	0.248	0
HOHLAR01	C10 H18 N2 O2	C2/c	15	C1CCC23NCCOC2(C1)NCCO3	0.69	0.232	2
HOLPII	C12 H18 N2 O1	P21/n	14	O=C1NC2(CCCCC2)NC2=C1CCC2	0.00	0.249	0
HOMQIJ	C14 H20	P21/n	14	C1CC2CCC1C1=C2C2CCC1CC2	1.26	0.167	3
HOMQUV	C14 H20 O1	P-1	2	C1CC2CCC1C13OC21C1CCC3CC1	0.00	0.160	0
HOSNEI	C11 H7 N1 O1	Pbca	61	O=C1N2C=CC=C2c2ccccc12	2.05	0.380	3
HOWBAW	C8 H16 N4	P-1	2	C1CN2CN3CCCN3CN2C1	0.16	0.328	2
HOWYID	C11 H11 N1 O2	Pna21	33	O=C1NC2CCOC2c2ccccc12	0.32	0.248	2
HOXRES	C4 F6	P21/c	14	FC1=C(F)C(F)(F)C1(F)F	0.26	0.123	4
HPANDO	C14 H10 O1	P21/a	14	O1C2C1c1ccccc1c1ccccc21	1.95	0.149	2
HPYDZO10	C4 H6 N2 O2	Ibca	73	O=C1CCC(=O)NN1	6.62	0.427	59
HQOXAL01	C8 H6 N2 O1	P212121	19	O=C1Nc2ccccc2N=C1	1.09	0.184	9
HQOXDO04	C8 H6 N2 O2	P21/n	14	O=C1Nc2ccccc2NC1=O	0.00	0.204	0
HURYUQ	C11 H14 O3	P1	1	C=C1CC2COCC3CC(=O)OC23C1	1.20	0.527	3
HURZAX	C13 H18 O2	P-1	2	C=C1CC2CCCC3CCC(=O)OC23C1	1.93	0.299	4

Table S3 (continued)

CSD Refcode	Formula	Space Group symbol	Space Group number	SMILES	Energy rank ΔE(CSP) / kJ/mol	$RMSD_{30}$ of CSP match / Å	Numerical rank
HUWGUB	C10 H10 O3	P-1	2	O=C1OCC23CC4C(CC12)C4C3=O	0.00	0.183	0
HXMTAM10	C6 H12 N4	I-43m	217	C1N2CN3CN1CN(C2)C3	0.00	0.074	0
HXQUIO02	C9 H7 N1 O1	P212121	19	O=C1Nc2ccccc2C=C1	0.54	0.288	5
IBESUD	C8 H11 N1 O2	C2/c	15	O=C1CCCC2=CCON1C2	6.65	0.431	73
IBETAK	C9 H13 N1 O2	C2/c	15	O=C1CCCCC2=CCON1C2	3.35	0.332	13
IBOHIT	C6 H2 N6 O2	P21/c	14	O=C1C2=NNN=C2C(=O)C2=NNN=C12	2.54	0.152	1
ICEANE	C12 H18	P63/m	176	C1C2CC3CC1C1CC2CC3C1	0.00	0.335	0
ICEFON	C8 H4 F4 O2	C2/c	15	Fc1c(F)c(F)c2OCCOc2c1F	1.30	0.395	2
IDIXEY	C4 F4	P21/c	14	FC(F)=C=C=C(F)F	1.69	0.282	6
IDOFAL	C10 H10 O1	P21	4	O=C1C=CC2C3CC(C=C3)C12	0.00	0.194	0
IDOFEP	C10 H10 O1	P21	4	O=C1C=CC2C3CC(C=C3)C12	0.00	0.187	0
IDOREY	C8 H12	P-1	2	C1CC(C1)=C1CCC1	0.00	0.208	0
IDOSAW01	C14 H11 N1 O1	P-1	2	O=C1Nc2ccccc2Cc2ccccc12	0.00	0.311	0
IFILEO	C10 H10 O3	P21/c	14	O=C1OC(=O)C2C3CCC(C=C3)C12	0.00	0.282	0
IHEPUG	C8 H11 N1 O2	P41212	92	O=C1NC(=O)C2CCCCC12	11.10	0.514	39
IHEPUG02	C8 H11 N1 O2	P212121	19	O=C1NC(=O)C2CCCCC12	None	None	None
IJISAX	C9 H16 N2 O2	Pca21	29	C1COC2(O1)C1CNCC2CNC1	0.00	0.158	0
IMARAR	C8 H9 N1 O4	P21/c	14	O=C1OCC2C3COC(=O)N3CC12	6.85	0.402	86
IMAZOL03	C3 H4 N2	P21/n	14	N1C=CN=C1	0.00	0.152	0
IMAZOL22	C3 H4 N2	Aba2	41	N1C=CN=C1	4.97	0.533	24
IMTRAZ	C5 H4 N4	P21/n	14	C1=NN2C=CN=C2N=C1	0.00	0.217	0
INDAZL02	C7 H6 N2	P21	4	N1N=Cc2ccccc12	3.44	0.929	10
INDDON	C9 H6 O2	I41/a	88	O=C1CC(=O)c2ccccc12	1.07	0.095	2
IQUNCO	C7 H11 N1 O1	P21/c	14	O=C1NC2CCC1CC2	0.00	0.142	0
ISATIN04	C8 H5 N1 O2	P21/c	14	O=C1Nc2ccccc2C1=O	0.00	0.112	0
ITIZOA	C4 H10 N2	P21/n	14	C1CNCCN1	2.28	0.345	4
ITIZUG	C4 H9 N1 O1	P212121	19	C1COCCN1	1.19	0.221	3
ITOBAU01	C5 H11 N1	P21/c	14	C1CCNCC1	1.15	0.218	5
IWUDEJ	C10 H13 N1 O2	P212121	19	O=C1CCC2CC3=CCOC3CN12	0.00	0.502	0

Table S3 (continued)

CSD Refcode	Formula	Space Group symbol	Space Group number	SMILES	Energy rank ΔE(CSP) / kJ/mol	$RMSD_{30}$ of CSP match / Å	Numerical rank
IYINAF01	C8 H10 O1	P21/c	14	C1C=CC2CC=CC1O2	0.00	0.262	0
IYUTIH	C12 H16 N2	P21/n	14	C1CCc2nc3CCCCc3nc2C1	0.00	0.279	0
IZALOM	C12 H12 O2	P21/n	14	O=C1CC2Cc3ccccc3CC2O1	0.40	0.262	2
IZIJOQ	C10 H15 N1 O2	P21/c	14	O=C1CCCCCC2=CCON1C2	3.58	0.261	14
JASGIT	C12 H12 O3	Pna21	33	O=C1CCC2=C1CCC1=C(C2)C(=O)OC1	15.77	1.837	741
JAYFEU	C12 H16	Pbca	61	C1C=CCC=CCC=CCC=C1	2.10	0.216	30
JAZDOD02	C12 H11 N1 O1	Pna21	33	O=C1CCCC2=C1c1ccccc1N2	0.28	0.302	2
JEDVET	C6 H9 N1 O1	P21/c	14	O=C1NC2CCCC12	0.96	0.133	5
JEHHEJ	C14 H18	P21/c	14	C1CC2CCCC(C1)C2=C1C=CC=C1	2.35	0.491	11
JEZZOD	C9 H8 N2 O1	I-42d	122	O=C1NC23C4C5C6C4C2(N1)C6C35	0.00	0.162	0
JIBFOP	C10 H14 O2	P21/n	14	O=C1CCCC2C1CCCC2=O	0.00	0.803	0
JICZEA	C15 H12 O1	Pna21	33	C1c2ccccc2C2OC2c2ccccc12	0.00	0.258	0
JIGJUE10	C12 H9 N1 O1	P-1	2	O=C1C=CC2=Cc3ccccc3CN12	0.00	0.292	0
JINZAI	C7 H8 O3	P21	4	O=C1CC2OC(CO1)C=C2	0.04	0.279	1
JITMEE	C13 H16 O1	P212121	19	O=C1C2C=CC1C1CCC=CCCC21	0.00	0.145	0
JIVRIS	C6 H4 O5	P21	4	O=C1CC2C(O1)C(=O)OC2=O	3.30	0.504	2
JIYTUG	C9 H15 N1 O2	P21/n	14	O=C1COCC2CCCCC2N1	0.00	0.227	0
JODCOW	C12 H14 N2 O1	P212121	19	O=C1CNCC2N1CCc1ccccc21	0.99	0.328	1
JOLGUM	C15 H10 O1	P21/c	14	O=C1Cc2cccc3c4cccc(C1)c4c23	2.96	0.421	12
JONSIO	C1 H1 F2 N1	P21/n	14	FC(F)=N	0.33	0.118	2
JONVOX	C13 H12	C2/c	15	C1CC2=C3CC(=CC=C2)C=CC=C13	4.15	0.237	26
JOPLOP	C13 H22 O1	Pbca	61	O=C1CC2CCCCCCCC(C2)C1	0.00	0.232	0
JOSLOU	C13 H11 N1	P21	4	C1c2ccccc2Nc2ccccc12	0.00	0.374	0
JUDZIR	C8 H12 O6	P21/c	14	C1COC23OCCOC2(O1)OCCO3	1.28	0.162	1
JUTCEI	C10 H14 O4	C2	5	C1CC2C(O1)OC1OC3OCCC3C21	0.00	0.288	0
JUVKAM	C8 H8 O2	Pbca	61	O=C1OC(=C2CC2)C21CC2	0.41	0.120	1
JUXJER	C9 H5 F1 O2	P21	4	Fc1ccc2C=CC(=O)Oc2c1	0.72	0.332	4
KAJRIY	C6 H10 N4 O1	P212121	19	[O]N1=NN2CCCCCC2=N1	4.84	0.270	14
KEFGUZ	C16 H12	P21/c	14	C1c2ccccc2C1=C1Cc2ccccc12	0.00	0.624	0

Table S3 (continued)

CSD Refcode	Formula	Space Group symbol	Space Group number	SMILES	Energy rank ΔE(CSP) / kJ/mol	$RMSD_{30}$ of CSP match / Å	Numerical rank
KEJYOP	C9 H11 N3 O1	P21/c	14	O=C1CN2C=CC=C2C2NCCN12	0.00	0.271	0
KEMHIT	C14 H11 N1 O1	P21/n	14	N1C2=CC=CC=C1C=C1OC(=C2)C=CC=C1	2.02	0.416	4
KEMTIG	C13 H12 O2	Pbcn	60	O=C1Oc2ccccc2C2C=CCCC12	1.10	0.182	3
KEMZIL01	C1 H2 O1	P-421c	114	C=O	2.60	0.464	190
KEPKIZ	C5 H2 N6 O2	P212121	19	[O]N1=C2C=CC3=NN=NN3C2=NO1	5.09	0.261	10
KEQSOQ	C7 H14 N2	P212121	19	C1NCC2CNCC1C2	0.15	0.370	1
KIGCEL	C9 H10 O3	C2/c	15	O=C1C=CC2CC3COCC13O2	0.24	0.249	1
KIMCOY	C8 H8 O2	P21/c	14	C1OCC#CCOCC#C1	0.00	0.173	0
KISGAW	C12 H7 N1 O1	P21/c	14	O=C1c2ccccc2c2ncccc12	0.00	0.792	0
KISGUQ	C12 H7 N1 O2	Pc21b	29	O=C1c2ccccc2Oc2ccncc12	0.00	0.193	0
KIXSIT	C13 H11 N1 O1	P212121	19	O=C1C=C2NCC3CC23c2ccccc12	0.00	0.261	0
KIZKOU	C8 H12 N2 O2	P43212	96	O=C1NC2CCCCC2NC1=O	0.00	0.227	0
KOBXOO	C3 H4 N4 O3	P21/n	14	[NH2+]=C1NNC(=O)C1=N(=O)[O-]	12.47	0.753	75
KOGFER	C13 H16 O2	P21/n	14	C=C1C(=O)OC21C1CC3CC(C1)CC2C3	0.37	0.247	1
KOJGOH	C11 H14 O3	P212121	19	O=C1CC2OCC3C2OC21CCCC32	0.47	0.228	1
KOJGUM	C9 H6 N4	C2/c	15	C1=CN2N=C3C=CC=NC3=C2N=C1	3.25	0.613	29
KOJGUN	C11 H12 O4	P21/c	14	O=C1OC2CC(=O)C34CCCC3C1C2O4	0.00	0.259	0
KORWOF	C4 H5 F2 N1 O2	P21/c	14	FC1(F)CNC(=O)OC1	0.13	0.154	1
KOVBEC	C13 H16	Pbcn	60	C1CC21C1(CC1)C12C2(CC2)C21CC2	2.49	0.448	16
KOXRIY	C2 H3 N3 O2	P21/n	14	O=C1NNC(=O)N1	4.31	0.307	24
KOYLIT	C6 H4 N4 O2	Pcab	61		2.25	0.218	10
KUKTEQ	C9 H6 O5	P21/n	14	O=C1OC2C=CC1C1C2C(=O)OC1=O	0.00	0.162	0
KUVCIP	C4 H4 N2	Pna21	33	c1cncnc1	0.30	0.178	2
LADVIV10	C12 H16 O2	P21/n	14	O=C1C2CCC31CCCC13CCC2O1	0.00	0.107	0
LAFJUX	C6 H8 O3	P212121	19	O=C1OCC2CCOC12	0.00	0.554	0
LAKDAD	C10 H10 O3	P212121	19	O1C2C1C1C3C4OC4C2C2OC2C13	0.00	0.220	0
LAKDEH	C10 H10 O3	P-1	2	O1C2C3OC4C5OC(C1C1C3C51)C24	0.00	0.244	0
LANZAC	C11 H12 O1	P21/n	14	O=C1C=C2CCC3CC23C21CC2	0.53	0.187	2
LAXFOH	C3 H2 F2	P21/c	14	FC=C=CF	0.26	0.233	1

Table S3 (continued)

CSD Refcode	Formula	Space Group symbol	Space Group number	SMILES	Energy rank ΔE(CSP) / kJ/mol	$RMSD_{30}$ of CSP match / Å	Numerical rank
LAXFUN	C3 H1 F3	P21/c	14	FC=C=C(F)F	1.36	0.152	11
LECZOL	C12 H6 O3	P65	170	O1C=Cc2c1c1C=COc1c1C=COc21	0.46	0.576	2
LEGPES	C12 H11 N1 O2	P21/c	14	O=C1CCC2N1Cc1ccccc1C2=O	5.80	0.390	32
LEHMOD	C12 H12 O2	Pbca	61	O=C1CCCC2C1Oc1ccccc21	0.00	0.206	0
LEHQIZ	C6 H7 N1 O2	P21/c	14	O=C1NC(=O)C2CCC12	4.35	0.189	8
LENYOS	C10 H12 O3	Pcab	61	C1COc2ccccc2OCCO1	0.00	0.281	0
LERGOG	C6 H5 N3 O1	P21/n	14	O=C1NC=NC2=C1NC=C2	6.81	2.067	23
LEXSIT	C12 H15 N1 O1	P212121	19	C1CC2(CCO1)CNc1ccccc21	0.93	0.184	1
LIJFUG	C5 H6 O3	P212121	19	O=C1CCCC(=O)O1	0.00	0.180	0
LINKOI	C14 H10 O1	Pbca	61	O=C1CCCC#Cc2ccccc2C#C1	0.59	0.249	4
LINMOK	C7 H13 N3	P21/n	14	C1CN2CCN3CCN1C23	2.40	0.269	17
LISXOB	C7 H8 O3	P212121	19	O=C1CC2C3CC3OC2O1	1.28	0.177	1
LIWPIT	C7 H9 N1 O2	P21/c	14	O=C1CC(=O)C2(CCC2)N1	4.19	0.338	9
LOGFAO	C8 H10 O3	Pc	7	O=C1OCCC1=C1CCCO1	5.55	0.348	53
LOVBIH	C13 H8 O2	P21/n	14	O=C1OC=Cc2c1ccc1ccccc21	4.19	0.242	16
LOVWIC	C3 H2 F2	P21/a	14	FC(F)=C=C	1.45	0.476	18
LOVWOI	C3 F4	Ccca	68	FC(F)=C=C(F)F	7.60	0.125	9
LPROGL04	C7 H10 N2 O2	P212121	19	O=C1CNC(=O)C2CCCN12	0.00	0.318	0
LUVCEK	C13 H15 N1 O1	P21/c	14	O=C1Nc2ccccc2C2CCCCC12	0.00	0.219	0
LUXVOP	C10 H12 N4	Ccca	68	C1C2CC3CC(CC1C13N=N1)C12N=N1	2.18	0.166	2
MAGXAV	C5 H4 F1 N1	Pbca	61	Fc1ccccn1	1.06	0.330	8
MAGXEZ	C5 H3 F2 N1	Pn	7	Fc1cccc(F)n1	0.72	0.230	6
MAMXIJ	C13 H9 N1	C2/c	15	c1ccc2cc3ncccc3cc2c1	2.97	0.152	15
MAYXEP	C10 H12 O2	P21/n	14	C1CC2OC1C1C3OC(C=C3)C21	1.40	0.170	7
MAYXIT	C10 H12 O2	P21/a	14	C1CC2OC1C1C3OC(C=C3)C21	1.85	0.185	9
MCYPDE	C16 H12	P21/a	14	c1cc2C=Cc3cccc(C=Cc(c1)c2)c3	0.00	0.208	0
MEBCEC	C10 H15 N1 O2	P21/c	14	O=C1CC2(CCCCC2)CC(=O)N1	2.22	0.147	5
MEGNES	C14 H8 N2	P42/mbc	135	c1cc2cccc3c4nccnc4c(c1)c23	None	None	None
MEKWUV	C15 H18	P21	4	C1CC21CC12CC21CC12CC21CC12CC1	2.35	0.549	4

Table S3 (continued)

CSD Refcode	Formula	Space Group symbol	Space Group number	SMILES	Energy rank ΔE(CSP) / kJ/mol	$RMSD_{30}$ of CSP match / Å	Numerical rank
MEMTED	C12 H10 N2	Pbca	61	N1c2ccccc2Nc2ccccc12	1.36	0.380	1
MEOXAL	C3 H2 O4	P41	76	O=C1OCOC1=O	6.42	0.480	40
METCEN	C16 H14	Pbca	61	C1Cc2cccc(C=Cc3cccc1c3)c2	1.52	0.429	5
METCYP	C16 H16	P21/c	14	C1Cc2cccc(CCc3cccc1c3)c2	0.22	0.445	1
MEXCUP	C5 H4 O3	P212121	19	C=C1CC(=O)OC1=O	0.00	0.170	0
MIBVUQ	C16 H16	P42/mnm	136	C1Cc2ccc(CCc3ccc1cc3)cc2	0.00	0.042	0
MILYIP	C6 H6 N2 O1	Pnma	62	O=C1NC=NC2=C1CC2	4.51	0.319	8
MIMTEI	C11 H11 N1 O1	P-1	2	O=C1CCC2Cc3ccccc3N12	0.00	0.538	0
MINZAN	C8 H7 N1 O2	P21/c	14	O=C1CC2(N1)C=CC(=O)C=C2	0.00	0.362	0
MINZOB	C9 H9 N1 O2	P21	4	O=C1CCC2(N1)C=CC(=O)C=C2	3.27	0.247	7
MIXPEO	C9 H5 N3 O2	P212121	19	O=C1C2=CN=NN2Oc2ccccc12	0.00	0.160	0
MLEICA01	C4 H2 O3	P212121	19	O=C1OC(=O)C=C1	0.18	0.194	1
MNBZDO	C14 H10 O1	Pbca	61	O=C1C=CC2=CC=CC3=CC=CC1=C2C3	1.45	0.144	5
MOFBUF02	C8 H6 N2 O2	P21/c	14	O=C1NC(=O)c2ccccc2N1	0.00	1.303	0
MOGMOM	C8 H10 F1 N1 O1	P212121	19	FC1=CCC2CCCN2C1=O	0.00	0.356	0
MPCYLP10	C16 H12	Pbca	61	c1cc2C=Cc3ccc(cc3)C=Cc(c1)c2	8.14	0.316	189
MTANNL	C11 H10	Fdd2	43	C1C2=CC=CC=C1C=CC=C2	0.47	0.240	1
MUMSET	C4 H1 F3 N2 O1	P21/c	14	FC1=NNC(=O)C(=C1F)F	4.87	0.206	18
MUXRAA	C13 H9 N1 O1	C2/c	15	O=C1C=CC=C2C=Cc3ccccc3N12	0.99	0.607	2
NADVIX	C7 H10	P21/n	14	C1CC21CC12CC1	1.01	0.940	8
NADVUJ	C9 H12	P212121	19	C1CC21CC12CC21CC2	1.01	0.327	13
NADWAQ	C11 H14	P21/c	14	C1CC21CC12C2(CC2)C21CC2	1.22	0.330	4
NADWEU	C8 H10	P21/c	14	C1CC1=C1CC21CC2	5.20	0.356	183
NADWIY	C10 H12	P-1	2	C1CC1=C1CC21CC12CC1	2.95	0.407	15
NADWOE	C10 H12	P21/m	11	C1CC1=C1C2(CC2)C21CC2	1.63	0.386	3
NAGVEZ	C8 H4 F1 N1 O2	P212121	19	Fc1ccc2C(=O)C(=O)Nc2c1	2.51	0.741	1
NAHBIH	C11 H19 N1 O1	P21/c	14	O=C1NCCCCC2CCCCC12	0.00	0.144	0
NAHBON	C9 H15 N1 O1	P21/c	14	O=C1NCCCC2CCCC12	0.00	0.226	0
NAHFOR	C10 H9 F1	Pnam	62	FC12C=CC3C(C=C1)C3C=C2	0.00	0.079	0

Table S3 (continued)

CSD Refcode	Formula	Space Group symbol	Space Group number	SMILES	Energy rank ΔE(CSP) / kJ/mol	$RMSD_{30}$ of CSP match / Å	Numerical rank
NAKHEP	C8 H4 F1 N1 O3	P21/c	14	Fc1ccc2OC(=O)NC(=O)c2c1	0.00	0.385	0
NALDOT	C12 H14 O1	P21/c	14	C1CC23OC(C=C2)C2(CC2)C23CC12	3.36	0.378	7
NALDUZ	C12 H14 O2	Pbca	61	C1OCC23OC(C=C2)C2(CC2)C23CC12	0.45	0.156	1
NAPCBU	C12 H10	P21/c	14	C1Cc2cc3ccccc3cc12	0.00	0.170	0
NAPCPR01	C11 H8	Pnma	62	C1c2cc3ccccc3cc12	0.00	0.262	0
NAPHQU01	C10 H6 O2	P21/c	14	O=C1C=CC(=O)c2ccccc12	2.83	0.775	3
NAPHTA39	C10 H8	P21/a	14	c1ccc2ccccc2c1	0.00	1.191	0
NAPOIM02	C12 H7 N1 O2	P21/n	14	O=C1NC(=O)c2cccc3cccc1c23	0.48	2.310	2
NAPTAN01	C12 H6 O3	P212121	19	O=C1OC(=O)c2cccc3cccc1c23	1.23	0.299	3
NAPTAN02	C12 H6 O3	P21/c	14	O=C1OC(=O)c2cccc3cccc1c23	0.00	0.311	0
NAPTYR11	C8 H6 N2	P21/c	14	c1cnc2ncccc2c1	0.00	0.398	0
NARGEV	C8 H12 O4	P212121	19	C1COC2(CCC3COC2O3)O1	0.00	0.184	0
NAWTAI	C8 H4 N2 O2	P21/c	14	O1C=Nc2cc3OC=Nc3cc12	0.00	0.281	0
NBONAN01	C9 H8 O3	P212121	19	O=C1OC(=O)C2C3CC(C=C3)C12	0.00	0.215	0
NBORAN12	C9 H8 O3	P212121	19	O=C1OC(=O)C2C3CC(C=C3)C12	0.00	0.247	0
NEDDOR	C7 H2 F4 N2 O1	P21/c	14	Fc1c(F)c(F)c2C(=O)NNc2c1F	1.01	2.933	1
NEDDUX	C7 H4 F2 N2 O1	P21/n	14	Fc1ccc2C(=O)NNc2c1F	8.82	0.700	145
NEHQAU	C4 H4 N2 O1	P21/n	14	[O-][n+]1cccnc1	0.00	0.218	0
NENTUV	C13 H12 O2	P212121	19	C1COc2cc3ccccc3cc2OC1	0.00	0.205	0
NICREW	C7 H8	I41cd	110	C1CC21C=CC=C2	0.00	0.187	0
NICRIA	C7 H10	P21	4	C1CC2(CC2)C=C1	3.73	0.291	87
NICROG	C10 H12	Pbca	61	C1CC21C=CC1(CC1)C=C2	0.00	0.168	0
NIHZOT	C12 H16 O2	Pbca	61	O=C1CC2CCC1C13OC1CCCC23	2.93	0.209	11
NIKMEB	C8 H12 O5	P212121	19	C1OCC2OC3COCOC3C2O1	2.23	0.393	4
NIKVUZ	C4 H4 O2	P212121	19	O=C1CCC1=O	3.03	0.279	2
NILYAI	C4 H7 N1 O1	P-1	2	O=C1CCCN1	0.33	0.191	1
NIVZOH	C9 H14 N2 O2	P21/c	14	O=C1NC(=O)C2(CCCCCC2)N1	0.00	0.267	0
NIZVAU	C8 H4 F1 N1 O2	P21/c	14	Fc1cccc2C(=O)C(=O)Nc12	2.26	0.978	9
NODXAH	C9 H10 O3	P21/c	14	O=C1CCCCC#CC(=O)OC1	0.36	0.245	1

Table S3 (continued)

CSD Refcode	Formula	Space Group symbol	Space Group number	SMILES	Energy rank ΔE(CSP) / kJ/mol	$RMSD_{30}$ of CSP match / Å	Numerical rank
NOJVIR	C9 H10	P212121	19	C1C=CCC21C=CC=C2	0.50	0.128	2
NOJVOX	C9 H8	P21/c	14	C1=CC2(C=C1)C=CC=C2	0.00	0.292	0
NOKKUU	C12 H10 O2	P-1	2	O=C1CCC2=C1Cc1ccccc1O2	0.00	0.311	0
NOLDOJ01	C3 H6 N6	P21/n	14	C1CNC2=NN=NN2N1	1.96	0.243	3
NOLJON	C16 H14	C2/m	12	C1CC23C=CC4(CCc5ccc1c2c45)C=C3	0.00	0.146	0
NORFUX	C9 H14 O3	C2/c	15	O=C1COC2CCCCC2OC1	0.00	0.156	0
NORFUX01	C9 H14 O3	P21/n	14	O=C1COC2CCCCC2OC1	9.50	1.605	224
NOSLIS	C7 H8 O3	Pnma	62	O=C1OC2CC=CCC2O1	0.00	0.175	0
NOYWAZ	C8 H8 O2	C2/c	15	O=C1C2CC3C1CC2C3=O	6.21	0.412	71
NPDCBU	C14 H12	P-1	2	C1Cc2cc3cc4CCc4cc3cc12	0.00	0.146	0
NTCHPE	C14 H10	Iba2	45	c1cc2cccc3C4C5C4C5c(c1)c23	5.30	1.739	888
NUHJUW	C10 H6 N4	P21/c	14	c1cnc2nc3cccnc3nc2c1	0.00	0.122	0
NUNLAJ	C7 H12 O4	Pbcn	60	C1COC2(OC1)OCCCO2	0.00	0.142	0
NUNLEN	C9 H12 O4	Pbcn	60	C=C1COC2(OC1)OCC(=C)CO2	0.00	0.227	0
NUQPIZ	C8 H12 O2	P212121	19	O=C1CC2CCCC(C2)O1	1.55	0.250	5
NURVUS	C10 H14 O4	P21/c	14	O=C1OC2COC3(CCCCO3)C1C2	0.00	0.280	0
NUTTIF	C1 H2 N4 O1	P41212	92	O=C1NN=NN1	15.56	0.594	469
NUVZAF	C10 H14 N2 O2	P21/c	14	O=C1NC2(CCC2)C(=O)NC21CCC2	0.00	0.354	0
OBEQUJ	C6 H4 N2 O1	Pbca	61	[N]=N=C1C=CC=CC1=O	1.98	0.391	19
OBIQUN	C11 H11 N1 O2	Fdd2	43	O=C1N2CCCOC2c2ccccc12	2.08	0.150	13
OCASIV	C6 H5 N3 O1	P21/n	14	O=C1NN=CC2=CC=CN12	0.00	0.428	0
OCSHYD	C10 H16 N2 O2	C2/c	15	O=C1NC(=O)C2(CCCCCCC2)N1	0.33	0.185	3
OCYDEO	C9 H14 O3	Pbca	61	O=C1CCCCC(=O)OCCC1	0.00	0.132	0
OGOWOW	C16 H12	P21/c	14	C1c2ccccc2C1=C1Cc2ccccc12	0.00	0.285	0
OHILIC	C10 H6 N2 O1	P21/c	14	O=C1N2C=CC=C2c2ncccc12	0.70	0.257	2
OHULAE	C9 H10 O3	P21nm	31	O=C1OC2C3CCC(C=C3)C2O1	1.30	0.140	9
OLIXAK	C12 H11 N1 O1	P21/c	14	O=C1N2CC=CC(C2)c2ccccc12	1.70	0.427	2
OMEVOR	C11 H7 N1 O1	P21/n	14	O1C=Nc2c1ccc1ccccc21	1.94	0.167	1
OPIKUU	C9 H10 O3	P212121	19	O=C1CCC21C=CC1COC2O1	0.00	0.185	0

Table S3 (continued)

CSD Refcode	Formula	Space Group symbol	Space Group number	SMILES	Energy rank ΔE(CSP) / kJ/mol	$RMSD_{30}$ of CSP match / Å	Numerical rank
OPUSAU	C6 H6 O4	P21/m	11	O=C1OOC(=O)C21CCC2	3.41	0.160	2
OPUSEY	C5 H4 O4	Cmcm	63	O=C1OOC(=O)C21CC2	4.53	0.645	2
OPUSIC	C7 H8 O4	P21/c	14	O=C1OOC(=O)C21CCCC2	0.00	0.289	0
OSEXEQ	C9 H6 N6	P-1	2	C1=CN2C(=N1)N1C=CN=C1N1C=CN=C21	0.00	0.502	0
OSITER	C8 H5 N1 O3	P21/n	14	O=C1NC2=C(C=COC2=O)C=C1	1.01	0.601	4
OTOGEL	C9 H8 O1	Cc	9	O=C1C2C=CC3C(C=C2)C13	7.22	0.196	88
OXAZDO	C3 H3 N1 O3	C2/c	15	O=C1CNC(=O)O1	0.00	0.176	0
OXAZIL11	C3 H5 N1 O2	P21/c	14	O=C1NCCO1	0.00	0.214	0
OXBOCT	C5 H8 O3	P21/c	14	C1OCC2OC2CO1	0.38	0.191	2
OXETES	C11 H13 N3	P21/c	14	C1CCN2C(=Nc3ccccc23)NC1	2.45	0.366	3
OXHEPA03	C8 H6 O4	P212121	19	O=C1OC(=O)C2C3OC(C=C3)C12	0.32	0.178	1
PABRAL	C11 H12 N2 O2	C2/c	15	O=C1CN2CCOC2c2ccccc2N1	2.64	0.513	3
PAHYON	C3 H4 N2 O2	C2/c	15	O=C1CNC(=O)N1	0.09	0.481	1
PAMTUS01	C16 H16	P21/n	14	C1Cc2ccccc2CCc2ccccc12	1.17	0.102	2
PARBAC03	C3 H2 N2 O3	P21/n	14	O=C1NC(=O)C(=O)N1	5.02	0.543	38
PAZDID	C6 H9 N3 O1	Pna21	33	O=C1NCCN2CCN=C12	5.19	0.365	58
PAZROZ	C5 H6 O4	P212121	19	O=C1OC2COCC2O1	4.06	0.249	1
PCYDOL01	C16 H12	P21/c	14	C1=Cc2ccc(cc2)C=Cc2ccc1cc2	0.14	0.095	8
PECWIE	C10 H12 O4	P21	4	O=C1CC2CC3(CCC2O1)CC(=O)O3	0.52	0.559	1
PECWOK	C10 H12 O4	P21/n	14	O=C1CC2(CCC3(CC2)CC(=O)O3)O1	0.00	0.131	0
PENSOR	C10 H14 O4	P21/c	14	C1COC2(CC3OCC4CC2OC34)O1	0.00	0.224	0
PERANT10	C14 H24	P21/c	14	C1CCC2CC3CCCCC3CC2C1	4.26	0.161	8
PERLAA	C7 H13 N3	P-1	2	C1CCN2CCN=C2NC1	1.21	0.176	1
PEWMIN	C10 H18 O3	P21/a	14	C1CCC2OCCOCCOC2C1	0.00	0.201	0
PEZFAE	C12 H17 N1 O1	P21/c	14	O=C1CC23CC4CC(CC(C4)C2N1)C3	0.00	0.199	0
PEZFIM	C11 H11 N1 O1	P212121	19	O=C1CC2Cc3ccccc3C2N1	0.00	0.326	0
PHALIM04	C8 H5 N1 O2	P21/n	14	O=C1NC(=O)c2ccccc12	1.55	0.206	8
PHENAN08	C14 H10	P21	4	c1ccc2c(c1)ccc1ccccc21	0.00	0.273	0
PHENAT02	C13 H9 N1	P212121	19	c1ccc2c(c1)cnc1ccccc21	0.00	0.268	0

Table S3 (continued)

CSD Refcode	Formula	Space Group symbol	Space Group number	SMILES	Energy rank ΔE(CSP) / kJ/mol	$RMSD_{30}$ of CSP match / Å	Numerical rank
PHENAZ04	C12 H8 N2	P21/n	14	c1ccc2nc3ccccc3nc2c1	0.00	1.193	0
PHENAZ11	C12 H8 N2	P21/n	14	c1ccc2nc3ccccc3nc2c1	1.88	0.104	5
PHTHAO	C8 H4 O3	Pna21	33	O=C1OC(=O)c2ccccc12	0.00	0.165	0
PHYPHM	C8 H9 N1 O2	P212121	19	O=C1NC(=O)C2CC=CCC12	0.00	0.632	0
PIGRAY	C10 H14 O4	P21/c	14	O=C1CC2OC3CCCOC3CC2O1	0.00	0.513	0
PIGTUX	C13 H14 F1 N1	Pbcn	60	Fc1cccc2C3=C(CCCCC3)Nc12	9.12	0.283	35
PIGWEK	C12 H12 F1 N1	C2/c	15	Fc1cccc2C3=C(CCCC3)Nc12	5.17	0.258	9
PIGWIO	C12 H12 F1 N1	P212121	19	Fc1ccc2C3=C(CCCC3)Nc2c1	1.17	0.196	3
PINNOP02	C11 H6 N2 O1	P21/c	14	O=C1c2cccnc2c2ncccc12	1.26	0.234	1
PINXAL	C2 H2 N2 O2	P21/n	14	[O]N1=CC=NO1	2.27	0.929	43
PIWLEO	C8 H12 F1 N1 O2	P212121	19	FC1CCCCC21COC(=O)N2	0.00	0.213	0
PLEIDN14	C14 H10	P21/c	14	C1=Cc2cccc3cccc(C=C1)c23	1.33	0.230	6
POBXAI	C6 H4 N2 O3	P21/c	14	O=C1NC(=O)C2=COC=C2N1	3.36	1.845	9
POSQET	C14 H18	P-1	2	C1CC2C=CC1C1C3CCC(C=C3)C21	0.19	0.240	1
POVZUW	C10 H18	P21/n	14	C1CCC2CCCCC2C1	0.00	0.211	0
PRELAN	C14 H24	C2/c	15	C1CCC23CCCCC2(C1)CCCC3	0.00	0.128	0
PRHDNA01	C6 H8 N2 O2	P212121	19	O=C1NC(=O)N2CCCC12	0.00	0.076	0
PRMDIN	C4 H4 N2	Pna21	33	c1cncnc1	0.31	0.203	2
PRMDIN20	C4 H4 N2	P21/n	14	c1cncnc1	1.81	0.299	17
PTERID11	C6 H4 N4	Pna21	33	c1cnc2ncncc2n1	0.00	0.215	0
PTOXEC	C5 H10 O5	Pbcn	60	C1OCOCOCOCO1	2.94	0.155	3
PUGDEB	C6 H3 F3	C2/c	15	Fc1cccc(F)c1F	0.00	0.761	0
PUMVOJ	C8 H7 N1 O3	P21/c	14	O=C1NC(=O)C2C3OC(C=C3)C12	3.84	0.385	4
PURINE	C5 H4 N4	Pna21	33	N1C=Nc2ncncc12	0.00	0.234	0
PVVAWA01	C6 H3 F3	I2/a	15	Fc1cc(F)cc(F)c1	0.22	0.911	1
PYMDON	C4 H4 N2 O1	P41212	92	O=C1NC=CC=N1	8.47	0.247	134
PYPCPE	C15 H11 N1	Fdd2	43	c1cc2C=Cc3ccc(cc3)C=Cc(c1)n2	0.00	0.145	0
PYRAZI	C4 H4 N2	Pmnn	58	c1cnccn1	0.00	0.290	0
PYRAZI03	C4 H4 N2	P21/n	14	c1cnccn1	0.00	0.400	0

Table S3 (continued)

CSD Refcode	Formula	Space Group symbol	Space Group number	SMILES	Energy rank ΔE(CSP) / kJ/mol	RMSD$_{30}$ of CSP match / Å	Numerical rank
PYRCEN	C14 H12	P21/n	14	C1Cc2ccc3CCc4ccc1c2c34	0.00	0.499	0
PYRDNO16	C5 H5 N1 O1	P41212	92	[O-][n+]1ccccc1	0.00	0.232	0
PYRENE10	C16 H10	P21/c	14	c1cc2ccc3cccc4ccc(c1)c2c34	2.76	0.852	25
PYRIDO02	C5 H5 N1 O1	P212121	19	O=C1NC=CC=C1	1.17	0.272	4
QAJYIJ	C5 H4 N4 O1	Pna21	33	O=C1NC2=NC=NN2C=C1	2.03	0.217	5
QARVOV	C4 H3 N1 O3	P21/c	14	O=C1NC=CC(=O)O1	0.00	0.535	0
QEBFIM	C14 H10 O1	P212121	19	O1C2C=CC1c1cc3cccc3ccc21	0.00	0.734	0
QEGSOL	C8 H8 O3	P41212	92	O1C2C3OC4C5OC(C1C35)C24	0.88	0.192	1
QEJQEC	C14 H15 N1	P212121	19	C1CCC2=C(C1)N1CCc3cccc2c13	0.32	0.400	1
QEJQUS	C13 H13 N1	Pnma	62	C1CC2=C(C1)c1cccc3CCN2c13	6.09	0.299	20
QEKQIG	C11 H14 N2 O2	C2/c	15	C1CC2(CCC3CON=C23)C2=NOCC12	4.84	0.824	16
QELCEP01	C12 H14 N2 O1	Pbca	61	O=C1NC2(CCCC2)Nc2ccccc12	0.00	0.427	0
QIBCEK	C12 H8 N2 O1	C2/c	15	O=C1NN=Cc2c1ccc1ccccc21	None	None	None
QIBCIO	C12 H8 N2 O1	C2/c	15	O=C1NN=Cc2ccc3ccccc3c12	0.00	0.227	0
QIBMUJ	C13 H13 N1 O1	P21/a	14	O=C1C=CC=CC2=C1NC1=C2CCCC1	6.58	0.220	32
QICTUU	C12 H9 N1 O2	Pc	7	O=C1CCC(=O)C2=Cc3ccccc3N12	7.65	0.217	152
QICWOO	C11 H9 N1 O2	P21/n	14	O=C1NC(=O)C2C1Cc1ccccc21	1.43	0.263	1
QIFHES	C11 H14 O2	Pbca	61	O=C1CCC(=O)C2C3CCC(C3)C12	2.17	0.417	13
QISRUG	C12 H12 O2	Pbca	61	O=C1C2(CC=CC2)C(=O)C21CC=CC2	1.98	0.247	2
QIVGAE	C8 H10 N2 O3	P21/c	14	O=C1NC(=O)C2=C(CCCOC2)N1	3.10	0.737	3
QIWJEM	C8 H8 O4	Pna21	33	O=C1OC(=O)C2C3CCC(O3)C12	2.41	0.290	16
QOPYOK	C8 H12 N2 O2	P21/c	14	O=C1NC(=O)C2(CCCCC2)N1	5.01	0.402	3
QOTWUR	C11 H12 O4	P212121	19	C1C2C3C4OC5OC6OC(O4)C2C6C1C35	0.00	0.160	0
QOTXAY	C10 H10 O4	Pnma	62	O1C2OC3OC4OC1C1C2C2C3C4C12	2.35	0.169	8
QQQAPG02	C7 H12	P21/m	11	C1CC2CCC1C2	0.84	0.274	4
QQQCIS01	C3 H6	Cmc21	36	C1CC1	0.52	0.345	1
QQQGMJ01	C9 H8 O1	P21/c	14	O=C1CCc2ccccc12	4.15	0.393	25
QUDREN	C9 H12 O3	P21	4	O=C1CC2OCOCC3CC1C23	0.00	0.271	0
QUHFUW	C6 H6 F6	P21	4	FC1C(F)C(F)C(F)C(F)C1F	2.18	0.304	1

Table S3 (continued)

CSD Refcode	Formula	Space Group symbol	Space Group number	SMILES	Energy rank ΔE(CSP) / kJ/mol	$RMSD_{30}$ of CSP match / Å	Numerical rank
QUHZUO	C12 H11 N1 O1	P21/n	14	O=C1CCCC2=C1Nc1ccccc21	2.98	0.341	31
QULNIV	C9 H12 O2	P6122	178	O=C1CCC2CC1CCC2=O	2.40	0.149	7
QUYZIU	C14 H16	P-1	2	C1CC21C=CC1C3C=CC(C21)C13CC1	0.00	0.156	0
RAFSID	C12 H16	P-1	2	C1CC2C1C1C2C2C3CCC3C12	0.00	0.846	0
RAVSOW	C6 N6 O3	P63/m	176	O=C1C(=[N+]=[N-])C(=O)C(=[N+]=[N-])C(=O)C1=[N+]=[N-]	0.00	0.070	0
REBCUX	C7 H11 N3 O1	P21/n	14	O=C1NN=C2CCCCCN12	1.56	0.335	2
REJVAE01	C5 H9 N3	P21/c	14	C1CN2CCN=C2N1	0.45	0.348	1
REJVEI	C6 H11 N3	C2/c	15	C1CNC2=NCCN2C1	0.25	0.332	1
REQQUA	C2 H3 N3 O1	Pn	7	O=C1NC=NN1	4.05	0.191	44
RETTUG	C8 H10 O4	P21	4	C1C2OC3OC3CC2OC2OC12	0.00	0.273	0
REVDIG	C10 H12 O4	P21/n	14	C1COC2(O1)C=CC1(OCCO1)C=C2	0.00	0.354	0
REWHIL	C6 H8 N4 O1	Pca21	29	O=C1CCCCN2N=NN=C12	0.01	0.129	1
REWHOR	C5 H6 N4 O1	Pna21	33	O=C1CCCN2N=NN=C12	8.32	0.198	109
REYRUK	C13 H16 O2	Pna21	33	C=C1CC23CC1C(=O)CC2CCCC3=O	1.75	0.149	1
REZVOK	C7 H6 N2	P212121	19	N1C=Cc2ccncc12	1.21	0.774	3
RIGZAJ	C10 H9 N1 O3	P212121	19	O=C1CCC2N1CC1=C(OC=C1)C2=O	1.96	0.322	2
RIJGAS	C14 H22 O1	P212121	19	O=C1CCCC2CCC3CCCCC3C12	0.00	0.181	0
RIPSER	C12 H17 N1 O2	P21/n	14	O=C1CCC2C(C1)C1CCCCN1C2=O	2.60	0.300	8
RIQHEE	C9 H12	Pbca	61	C1CC2C=CC1C12CC1	0.00	0.118	0
RIQHII	C9 H10	P21/c	14	C1CC21C1C=CC2C=C1	0.00	0.200	0
RIQHOO	C9 H14	P21/n	14	C1CC2CCC1C12CC1	0.41	0.362	2
RIQVOC	C14 H20	Pbca	61	C1CCC#CCCCCCC#CCC1	0.00	0.258	0
RITNOY	C5 F5 N1	P21/c	14	Fc1nc(F)c(F)c(F)c1F	0.00	0.825	0
ROHBUL	C5 H8 N4 O2	Pbca	61	O=C1NC2CC(N1)NC(=O)N2	0.00	0.306	0
ROPPEU	C10 H12 O2	P21/c	14	O=C1C2CCCC1C1OC2C=C1	2.49	0.691	5
ROPPIY	C9 H10 O2	P21/c	14	O=C1C2CCC1C1OC2C=C1	0.00	0.259	0
ROPPUK	C11 H14 O1	Pccn	56	O=C1C2CCCC1C1CC2C=C1	1.65	0.147	2
ROTRAW	C11 H12 N4	P21/c	14	C1Cc2ccccc2CCC2=NN=NN12	6.84	0.933	57
RUFNUD	C12 H16 O3	Cc	9	O=C1CCCC2=C1CC1CCCOC1O2	4.20	0.366	20

Table S3 (continued)

CSD Refcode	Formula	Space Group symbol	Space Group number	SMILES	Energy rank ΔE(CSP) / kJ/mol	$RMSD_{30}$ of CSP match / Å	Numerical rank
RUHTET	C9 H11 N1	P21/c	14	C1CC#CCNCC#CC1	0.00	0.239	0
RUHTEV	C10 H12 O3	Pbca	61	O=C1OC2OCCC32CC=CCC13	1.60	0.190	3
RUHTOD	C8 H10 N2	P21/n	14	C1NCC#CCNCC#C1	1.34	0.344	1
RUJNOB	C14 H10 N2	C2/c	15	C1N2c3ccccc3N=C2c2ccccc12	7.01	0.591	83
RUJYIG	C10 H6 N2 O1	P21/c	14	O=C1N2C=CC=C2c2cccnc12	1.10	0.290	5
RUNBAF	C8 H10 O4	Pca21	29	O=C1CC2C3CCOC3OC2O1	0.00	0.111	0
RUVQII	C4 H5 N1	Pnma	62	N1C=CC=C1	0.00	0.253	0
RUVXAH	C8 H12 O2	P21/n	14	C1CC2OC1C1CCC2O1	0.00	0.162	0
RUVZEN	C6 H7 N1 O1	P212121	19	O=C1NC2CC1C=C2	3.14	0.160	8
RUWHEW	C12 H16 O2	Pbca	61	O=C1CCCC2C3CCCC(=O)C3C12	0.94	0.286	1
RUWHIA	C12 H16 O2	P21/n	14	O=C1CCCC2C1C1CCCC(=O)C21	4.37	0.395	6
SACBAA02	C1 O2	Pa3	205	O=C=O	0.00	0.223	0
SADBII	C10 H14 N2	P21/c	14	C1CC2C=CC1C1CCC2N=N1	0.00	0.158	0
SADHUA	C7 H8	P21/c	14	C1CC2C34CC23C14	0.00	0.162	0
SADJEM	C8 H10	P21/c	14	C1CC2C34CC23C4C1	0.00	0.166	0
SAJHIV	C6 H7 N3 O1	P21/n	14	O=C1NCCC2=CN=CN12	2.10	0.387	3
SALLIB	C10 H13 N1 O1	Pbca	61	C1CC2CCC3=NOC(C=C1)C23	1.50	0.290	2
SALLOH	C11 H15 N1 O1	P21/c	14	C1CC2CCC=CC3ON=C(C1)C23	0.00	0.072	0
SALMEY	C10 H11 N1 O1	P21/c	14	C1CC2=NOC3C=CC=CC1C23	2.57	0.193	4
SALMIC	C11 H13 N1 O1	P21/c	14	C1CC2C=CC=CC3ON=C(C1)C23	0.00	0.159	0
SATCUL	C4 H6 N2 O2	P21/c	14	O=C1NCCNC1=O	5.78	0.214	13
SATMIK	C14 H16 O1	P-1	2	O=C1C2CCCCC2Cc2ccccc12	0.00	0.223	0
SAWZAS	C12 H12 O2	P21/c	14	O=C1C2C3C4C=CCC4C(C23)C21CO2	0.54	0.158	1
SAYTER	C11 H14 O2	P21/n	14	O=C1CCCCC23OC(CC12)C=C3	0.24	0.401	1
SCPCBU01	C12 H16	P21/c	14	C1CC21C1(CC1)C1(CC1)C12CC1	0.56	0.618	18
SEGBUB	C7 H12 O4	P21/c	14	C1OCC2(CO1)COCOC2	0.46	0.243	1
SEGCAI01	C4 H6 O3	P21/n	14	O=C1OCCCO1	0.00	0.187	0
SELCAP	C13 H12 O1	P21/c	14	O=C1CCCC#CCc2ccccc12	3.58	0.330	3
SELCES	C11 H9 N1 O3	P21	4	O=C1OC(=O)N2Cc3ccccc3CC12	0.00	0.239	0

Table S3 (continued)

CSD Refcode	Formula	Space Group symbol	Space Group number	SMILES	Energy rank ΔE(CSP) / kJ/mol	$RMSD_{30}$ of CSP match / Å	Numerical rank
SEMMEE	C13 H13 N1	P21	4	C1CCc2nc3ccccc3cc2C1	0.00	0.170	0
SEMSEJ	C11 H8 N2	P21/n	14	N1c2ccccc2c2cnccc12	5.95	0.599	45
SEMSEJ01	C11 H8 N2	Pna21	33	N1c2ccccc2c2cnccc12	1.36	0.217	3
SEMVEL	C16 H10	P21/c	14	C12=C3C4=CC=C3C=CC=C1C=CC2=CC=C4 SEMVEL	0.00	0.210	0
SIBJIX	C7 H8 O3	P212121	19	O=C1CC2C(CC3OC23)O1	0.00	0.393	0
SIHVAG	C8 H10 N2 O2	C2/c	15	O=C1NC2(CC2)C(=O)NC21CC2	0.00	0.400	0
SINDNC	C15 H12	Pbca	61	C1=CC2C(C=C1)C12C=Cc2ccccc12	0.00	0.346	1
SISFUY	C3 H2 N6	P212121	19	C1=NN2C=NN=C2N=N1	14.33	0.633	386
SIWFUZ	C12 H8	P21/n	14	C1c2cc3cc4Cc4cc3cc12	0.85	0.303	5
SIZZUW	C12 H7 F1 N2	C2/c	15	Fc1ccc2nnc3ccccc3c2c1	0.00	0.245	0
SOCHIB	C10 H12 O3	P21/c	14	O=C1CCCC2C3OC(CC3=O)C12	0.00	0.234	0
SOFTEM	C10 H10 O3	P212121	19	O1C2C1C1C3OC3C3C4OC4C2C13	0.00	0.234	0
SOKPEP	C8 H16 N4 O2	P21/c	14	C1CN2NC3COCCN3NC2CO1	3.55	0.267	17
SOWQOL	C9 H9 N1 O1	P21/c	14	O=C1NCCc2ccccc12	2.38	0.228	9
SPIRDO	C11 H14 O1	P212121	19	O=C1C=CC2(CCCCC2)C=C1	0.00	0.104	0
SPIROC	C15 H20	Pbca	61	C1CC21C1(CC1)C1(CC1)C1(CC1)C12CC1	7.05	0.431	33
STZPDZ10	C5 H4 N4	P21/c	14	C1=CC2=NN=CN2N=C1	0.00	0.259	0
SUCANH12	C4 H4 O3	P212121	19	O=C1CCC(=O)O1	0.00	0.142	0
SUCCIN03	C4 H5 N1 O2	Pbca	61	O=C1CCC(=O)N1	1.29	0.219	3
SUKXEB	C12 H12	R-3c	167	C12C3C1C1C4C2C2C3C1C1C2C41	0.00	0.130	0
SURHOC	C11 H12 O1	Pbca	61	O=C1C2=C1CCCC#CCCC2	0.00	0.162	0
SURSED	C12 H20 N2	P-1	2	C1CC2CC(C1)N1C3CCC(CC3)N21	3.02	0.295	12
SUSCOY	C7 H4 F2	Pbca	61	FC1(F)c2ccccc12	1.22	0.187	2
SUSDAL	C4 H6	P21/c	14	C=C1CC1	1.82	0.348	23
SUVCUJ	C11 H13 N1 O1	P21/c	14	O=C1CCCCc2ccccc2N1	0.00	0.123	0
SUVDAQ	C11 H13 N1 O1	Pbca	61	O=C1CCCc2ccccc2CN1	4.24	0.427	18
SUWCOC	C16 H14	P21/c	14	C1CC2c3ccccc3C1c1ccccc21	0.00	0.097	0
SUZGOK	C11 H13 N3 O1	Pbca	61	O=C1NC2(CCCC2)Nc2ncccc12	0.00	0.381	0
TADKUH	C10 H10 N2 O1	P212121	19	O=C1NC2C1NCc1ccccc21	7.13	0.957	39

Table S3 (continued)

CSD Refcode	Formula	Space Group symbol	Space Group number	SMILES	Energy rank ΔE(CSP) / kJ/mol	$RMSD_{30}$ of CSP match / Å	Numerical rank
TAFKAM	C4 H6 N4 O1	P21/n	14	C1CNC2=NON=C2N1	8.38	0.122	46
TAGQIB	C11 H8 N2 O1	P21/n	14	O=C1Nc2cc3ccccc3cc2N1	5.62	1.270	12
TAGQOH	C7 H4 F2 N2 O1	P21/c	14	Fc1cc2NC(=O)Nc2cc1F	0.00	0.399	0
TANGUK01	C11 H11 N1 O3	P21/c	14	O=C1Nc2ccccc2C21OCCCO2	0.92	0.284	1
TANMAT	C6 H8 O3	P41212	92	C1OC2C3COC2C1O3	1.89	0.154	4
TARZUH	C9 H7 N3	P21/n	14	N1c2ccccc2N2C=CN=C12	5.89	0.605	15
TAVPEN	C16 H10	P21/c	14	c1ccc2c(c1)C=C1c3ccccc3C=C21	0.00	1.088	0
TAVTUH	C13 H16 O2	P-1	2	C1CC2=C(C1)C1(OCCO1)C1=C2CCC1	12.03	1.751	795
TAXNIR	C13 H14 O2	P21/n	14	O=C1CCc2ccc3OCCCCc3c12	0.00	0.158	0
TAXRIT	C11 H6 O2	P21/n	14	O=C1C=CC(=O)c2cc3Cc3cc12	1.70	0.364	5
TAZCAZ	C8 H5 N5	P21cn	33	c1cc2ncnc3ncnc(c1)n23	1.19	0.222	5
TAZPYD10	C5 H4 N4	P21	4	C1=CC2=NC=NN2N=C1	0.00	0.300	0
TAZTCD01	C8 H16 N4	I-42m	121	C1CN2CN3CCN(CN1C3)C2	0.70	0.643	1
TCDECA	C12 H10 O3	P21/c	14	O=C1OC(=O)C2C3C=CC(C4C=CC34)C12	0.12	0.202	1
TCDEDO10	C12 H12 O2	P21/c	14	O=C1C(=O)C23CC=CCC12CC=CC3	0.62	0.138	1
TCDEOC	C10 H10 O2	P21/n	14	O=C1CC2C3C=CCC(C13)C2=O	2.50	0.194	9
TCDEPX	C12 H16 O2	Pbca	61	C1CC23CCC41OC14CCC2(CC1)O3	2.11	0.385	8
TCDODO	C13 H16 O2	C2/c	15	O=C1CC2C3CCC(C3)C32CC1CC3=O	0.00	0.195	0
TCDODP	C12 H12 O2	P21/n	14	O=C1C2CC=CC3C4CC(C14)C(=O)C23	2.24	0.186	6
TCTDON	C13 H18 O1	P21/c	14	O=C1CC2CCCC3CCCC(=C1)C23	0.00	0.100	0
TCUNDO	C11 H14 O2	P212121	19	O=C1C2CC3CC(C2)CC(C3)C1=O	2.18	0.237	3
TCUNDP	C11 H16 O1	P21/c	14	O=C1CC2CC3CCC2C(C3)C1	0.00	0.143	0
TEKQAB01	C4 H3 N1 O2	C2/c	15	O=C1NC(=O)C=C1	2.61	0.208	21
TEMCAP	C6 H8 O2	C2/m	12	C1C2CC3OC1C3O2	0.00	0.133	0
TEOXDE	C6 H12 O4	P-1	2	C1COCOCCOCO1	1.07	0.167	3
TETDAM08	C6 H12 N2	P63/m	176	C1CN2CCN1CC2	0.00	0.205	0
TETRAZ02	C2 H2 N4	P21/n	14	c1nncnn1	0.05	0.536	1
TETZOL02	C1 H2 N4	P1	1	N1C=NN=N1	0.00	0.815	0
TEVWUM	C12 H12 O3	P21/m	11	C=C1C2OC2C(=C)C2OC2C(=C)C2OC12	0.00	0.178	0

Table S3 (continued)

CSD Refcode	Formula	Space Group symbol	Space Group number	SMILES	Energy rank ΔE(CSP) / kJ/mol	$RMSD_{30}$ of CSP match / Å	Numerical rank
TEZPDZ10	C4 H3 N5	P21/n	14	C1=CC2=NN=NN2N=C1	0.00	0.097	0
TFBENQ02	C6 F4 O2	P21/c	14	FC1=C(F)C(=O)C(=C(F)C1=O)F	0.00	0.552	0
TFMETH02	C1 F4	C2/n	15	FC(F)(F)F	4.40	1.332	288
TICJEU	C16 H16	P21/c	14	C1Cc2ccc3CCCc4ccc(C1)c2c34	1.85	0.339	8
TIFKOJ	C11 H7 N1 O3	Pbca	61	C=C1N2C(OC1=O)c1ccccc1C2=O	0.00	0.124	0
TIFXOV	C7 H10	P21/m	11	C1CC(C1)=C1CC1	1.10	0.215	2
TIHWIQ	C10 H12	P-1	2	C1CC21CC2=C1CC21CC2	4.85	1.282	90
TIMCHX	C6 H9 N3	P21/c	14	N1C2C1C1NC1C1NC21	0.00	0.218	0
TIPVIZ	C8 H7 N3	P21/n	14	N=C1NC(=N)c2ccccc12	13.01	0.644	51
TISMUG	C13 H19 N1 O1	P21/n	14	O=C1CCCCCCCCC2=C1C=CN2	1.07	0.392	3
TISNAN	C13 H19 N1 O1	P21/n	14	O=C1CCCCCCCCC2=CC=C1N2	0.00	0.315	0
TIXQEY	C14 H8 N2	P21/c	14	c1ccc2c(c1)N=C1c3ccccc3N=C21	1.68	0.418	6
TMENBZ02	C15 H18	P21/n	14	C1Cc2c(C1)c1CCCc1c1CCCc21	3.48	0.353	8
TOAZCD	C5 H9 N1 O5	P21/c	14	O=C1NCOCOCOCO1	0.00	0.157	0
TOHZUL	C11 H12 O2	P-1	2	O=C1CC2C3CC4C2CC(=O)C4C13	0.00	0.199	0
TOMCOP	C11 H7 N1 O3	Fdd2	43	O=C1ON=C2COc3ccccc3C=C12	0.00	0.165	0
TORQIC	C8 H7 N1 O1	Pbca	61	O=C1CCc2ncccc12	2.62	0.167	7
TOXCDP	C8 H16 O4	P-1	2	C1COCCOCCOCCO1	2.88	0.256	9
TOXCTD	C10 H20 O4	P21/n	14	C1COCCOCCCOCCOC1	3.76	0.469	18
TOXDEC	C6 H10 O4	C2/c	15	C1COC2OCCOC2O1	0.00	0.111	0
TOXOCN	C4 H8 O4	C2/c	15	C1OCOCOCO1	1.34	0.781	4
TOXSTD	C10 H16 O4	P21/c	14	C1COC2(CCC3(CC2)OCCO3)O1	1.35	0.166	5
TOXTDC	C10 H20 O4	P21/n	14	C1CCOCOCCCCOCOC1	0.50	0.151	2
TOYSIL	C4 F4 N2	Pna21	33	Fc1nc(F)c(F)c(F)n1	2.54	0.123	1
TRAZOL02	C2 H3 N3	Pbca	61	N1C=NC=N1	0.35	0.204	2
TRIZIN	C3 H3 N3	R-3c	167	c1ncncn1	0.00	0.136	0
TRIZIN05	C3 H3 N3	C2/c	15	c1ncncn1	0.00	0.393	0
TROXAN11	C3 H6 O3	R3c	161	C1OCOCO1	0.41	0.130	1
TRQUIN	C10 H10	R-3	148	C1=CC2C=CC3C=CC1C23	0.00	0.089	0

Table S3 (continued)

CSD Refcode	Formula	Space Group symbol	Space Group number	SMILES	Energy rank ΔE(CSP) / kJ/mol	$RMSD_{30}$ of CSP match / Å	Numerical rank
TSCPCP02	C9 H12	P63/m	176	C1CC21C1(CC1)C12CC1	0.00	0.068	0
TUCNIQ	C11 H12 O3	P21/c	14	O=C1C2CC3COCC3(C=C2)C21CO2	1.99	0.279	2
TUGQAP	C13 H9 N3	P21/n	14	c1ccc2N3C=CN=C3C3=CC=CN3c2c1	0.00	0.230	0
TUNWUV	C10 H9 N1 O3	P21/c	14	O=C1Nc2ccccc2C21OCCO2	9.55	1.103	55
TUNYUY	C8 H10 N2 O2	P212121	19	O=C1C2CCN2C(=O)C2CCN12	0.00	0.333	0
TUVWOX	C11 H8 O2	P-1	2	O=C1OC2Cc3ccccc3C2=C1	2.14	0.321	1
TUWWUD	C9 H5 F2 N1 O1	Pnma	62	FC1(F)C(=O)C=Cc2ncccc12	0.00	0.194	0
TXBNON	C6 H8 O4	P21/c	14	O=C1CC2OCCOC2O1	0.00	0.166	0
TXCTDD	C10 H16 O4	P21/c	14	C1OCC=CCOCOCC=CCO1	3.05	0.385	11
TXTATD	C9 H15 N1 O3	P21/c	14	C1CC2OCCC3OCCC(O1)N23	0.00	0.107	0
UCICIV	C12 H12 O2	P21/n	14	O=C1CC2CC1C1C=CC=CC2C1=O	0.00	0.222	0
UDALIV	C4 H12 N6	P21/n	14	C1NNCN2CNNCN12	0.00	0.357	0
UDEXUZ	C8 H9 F1 O5	P212121	19	FC1OCOC2C1OC1CC(=O)OC21	7.41	0.725	18
UFEMEX	C10 H8 O2	P21/c	14	O=C1C=CC2C3C=CC(C12)C3=O	1.76	0.182	11
UFIYUD	C5 H6 N2 O3	P212121	19	O=C1NC2COC(=O)C2N1	5.30	0.504	7
UHACIP	C9 H10 O4	P212121	19	O=C1CC23CCCC2(CC(=O)O3)O1	0.00	0.183	0
ULIMEH	C11 H14 O2	C2/c	15	O=C1CC2OC3C1CCCCC3=C2	2.06	0.329	6
UMERAF	C9 H12 N4	P21/n	14	C1C2N=NC3CC4N=NC1C4CC23	0.86	0.132	5
UMILUZ	C9 H6 O2	P21/n	14	O=C1C=COc2ccccc12	3.53	0.559	38
UMIMIO	C9 H5 F1 O2	P1	1	Fc1ccc2OC=CC(=O)c2c1	0.00	0.397	0
UNDECO	C11 H20 O1	P21/c	14	O=C1CCCCCCCCCC1	1.46	0.308	6
UNEFEA	C14 H10 N2	P21/c	14	N1c2ccccc2C2=C1c1ccccc1N2	0.27	0.376	1
UQILOW	C11 H10 N2 O1	P21	4	C1OCC2=Nc3ccccc3N2C=C1	0.00	0.661	0
URACIL	C4 H4 N2 O2	P21/a	14	O=C1NC=CC(=O)N1	1.34	0.241	18
URAZIX	C9 H8 O2	P21/c	14	O=C1CCOc2ccccc12	0.69	0.184	1
URICAC	C5 H4 N4 O3	P21/a	14	O=C1NC(=O)C2=C(N1)NC(=O)N2	8.24	0.957	105
UTUSUZ	C9 H6 O4	P21/n	14	O=C1OCc2cc3OCOc3cc12	0.00	0.365	0
UWUYAO	C9 H13 N1 O1	P-1	2	O=C1NC2CC2CC2CCC12	0.00	0.270	0
UXAZID	C4 H3 N5	P21/c	14	C1=CN2N=NN=C2N=C1	0.34	0.229	2

Table S3 (continued)

CSD Refcode	Formula	Space Group symbol	Space Group number	SMILES	Energy rank ΔE(CSP) / kJ/mol	$RMSD_{30}$ of CSP match / Å	Numerical rank
UZIKEV	C11 H11 N1 O1	Pbca	61	O=C1CCc2cccc3CCN1c23	4.41	0.360	35
UZOVOV	C11 H12 O3	Aba2	41	O=C1OC23CC=CCC2(CC=CC3)O1	1.65	0.049	5
UZOVUB	C11 H16 O3	C2221	20	O=C1OC23CCCCC2(CCCC3)O1	0.73	0.125	2
UZOWAI	C11 H8 O3	C2/c	15	O=C1OC23C=CC=CC2(O1)C=CC=C3	0.37	0.345	1
VACCEH	C6 H8	Pbca	61	C1C=CCC=C1	0.20	0.115	1
VAJDIT	C12 H16 O2	C2/c	15	O=C1CC2CCCC(=O)C32CCCC13	0.00	0.492	0
VAJGOC	C5 H8	R-3	148	C1CC21CC2	1.47	0.212	26
VAJGUI	C7 H10	Pbcn	60	C1CC2CC32CC13	0.00	0.284	0
VAJHAP	C11 H10	P21/c	14	C1C2c3ccccc3C3CC123	2.38	0.237	9
VAKJOG	C8 H6 N2 O2	P21	4	[O]N1C=CN([O])c2ccccc12	0.00	0.697	0
VALZIR	C9 H8	C2/c	15	C1Cc2cc3Cc3cc12	1.25	0.232	6
VAMZIT01	C10 H10	P21/n	14	C1=CC2C3C4C1C1C2C3C41	0.96	0.254	5
VANLAX	C10 H10 O4	P21/c	14	O=C1CC2OC3=C(C2O1)C(=O)CCC3	1.73	0.241	1
VANYEO	C8 H6 O2	P-1	2	O=C1C=CC2C1C=CC2=O	3.34	0.260	14
VARYUI	C15 H18	P21/n	14	C1C2CC3CC1CC(C2)C3=C1C=CC=C1	1.45	0.156	4
VARZOD	C13 H16 O1	P212121	19	O=C1CCCC(CCC1)=C1C=CC=C1	4.92	0.290	13
VATXAP	C12 H19 N1 O2	P21/n	14	O=C1NC2(CCCC2)OC2CCCCC12	2.18	0.373	4
VAWFUX	C11 H8 N2	Ia	9	N1c2ccccc2c2ncccc12	0.00	0.225	0
VAWTOE	C10 H12 O3	C2/c	15	O=C1CC2OCC3=CCCCC23O1	2.03	0.102	1
VEBFIR	C8 H11 N1 O2	P21cn	33	O=C1CCN2CC1CCC2=O	1.35	0.242	10
VEDTON	C12 H14 O2	C2/c	15	O=C1CCC2C3CC4C2CC(=O)C4C13	0.13	0.195	1
VEFCAK	C14 H16	P21/c	14	C1CC=C2C(=CC1)C1=CCCCC=C21	0.00	0.160	0
VEHCET	C6 H8 F4	P21/n	14	FC1CC(F)C(F)CC1F	1.09	0.282	1
VENYUI	C12 H12	Pbcn	60	C=C1C2C3C2C(=C)C2C1C2C3=C	0.00	0.099	0
VENZAP	C9 H6 O3	Pbcn	60	O=C1C2C3C2C(=O)C2C1C2C3=O	0.00	0.218	0
VEXPUJ01	C10 H8 O2	P21/n	14	O1C2C3OC3c3ccccc3C12	0.00	0.196	0
VEYYII	C9 H10 O3	P21/c	14	O=C1CCCC23CCC(=O)C12O3	0.32	0.294	1
VEYYUU	C11 H20 O2	P21/c	14	C1CCCCC2(CCC1)OCCO2	0.85	0.261	1
VICPAY	C16 H14	P21/n	14	C1C2Cc3ccccc3C2c2ccccc12	6.56	0.345	59

Table S3 (continued)

CSD Refcode	Formula	Space Group symbol	Space Group number	SMILES	Energy rank ΔE(CSP) / kJ/mol	$RMSD_{30}$ of CSP match / Å	Numerical rank
VIDHAR10	C8 H10 N2 O2	P21/n	14	O=C1NC(=O)N2CCC3CC1C23	5.32	0.342	31
VIGKUT	C12 H6 O3	C2/c	15	O1C=C2C(=C1)C1=COC=C1C1=COC=C21	1.32	0.258	2
VIGTUA	C12 H8 N2	P21/n	14	c1ccc2nc3C4C5C4C5c3nc2c1	7.41	0.216	71
VIHJUR	C12 H11 N3	P21/c	14	C1C2NC3=Nc4ccccc4N3C1C=C2	0.75	0.476	3
VIKNAE	C10 H10 O4	P21/a	14	O=C1CCC2(O1)OC(=O)C1CC=CC21	1.77	0.326	2
VILQOY	C12 H14 O2	P21/n	14	O=C1CC23C=CC1CC2CCCC3=O	0.00	0.151	0
VINGOO	C9 H16 O2	Pbca	61	O=C1CCCCCCCCO1	0.00	0.069	0
VINSAN	C8 H3 F4 N1	C2/c	15	FC1=C(F)C2=CNC=C2C(=C1F)F	0.00	0.168	0
VIYXUZ	C11 H11 N1 O1	P212121	19	O=C1CC2Cc3ccccc3C2N1	0.00	0.339	0
VOBJAX	C8 H12 O4	P212121	19	O=C1CCOC2(COCCO2)C1	0.00	0.313	0
VOBJEB	C4 H4 N2	P21/n	14	c1ccnnc1	0.28	0.969	4
VOPHEN	C14 H16	P21/c	14	C1CC21C(=C1C3(CC3)C31CC3)C12CC1	2.53	0.377	4
VOPJOB	C7 H2 F4 N2 O1	P21/c	14	Fc1c(F)c(F)c2NC(=O)Nc2c1F	2.26	2.111	6
VOPPAU	C11 H8 O2	P-1	2	O=C1CCC2=C1c1ccccc1O2	0.50	0.200	1
VORSUQ	C8 H10	Pbca	61	C=C1C=CCC21CC2	0.00	0.170	0
VOXLOJ	C14 H8 O2	P21/n	14	O1C=Cc2ccc3OC=Cc4ccc1c2c34	0.00	0.263	0
VUXQAI	C7 H10 O5	C2221	20	C1OCC2OC3OCOC3C2O1	0.00	0.356	0
WACYAB	C12 H16 O2	C2/c	15	C1C=CCC2OC2CC=CCC2OC12	9.81	0.721	150
WACYEF	C12 H16 O3	P21	4	C1C=CCC2OC2CC2OC2CC2OC12	10.06	0.975	70
WAFHAO01	C4 H2 N2 O4	P21/n	14	O=C1NC(=O)C(=O)NC1=O	4.92	0.748	9
WAHBOX	C5 H4 N4 O2	P-1	2	O=C1NC(=O)N2C=NC=C2N1	4.37	0.292	12
WAPTOW	C11 H16 N2	P21/n	14	C1CC2CC1N1C3CCC(C=C3)N21	0.00	0.218	0
WAPTUC	C11 H18 N2	P21/n	14	C1CC2CCC1N1C3CCC(C3)N21	0.00	0.321	0
WAPVAK	C12 H20 N2	P21/c	14	C1CC2CCC(C1)N1C3CCC(C3)N21	0.00	0.210	0
WAPVIS	C13 H22 N2	P21/n	14	C1CC2CCC(C1)N1C3CCC(CC3)N21	0.00	0.309	0
WARPOW	C10 H7 N3 O1	P21/n	14	N1c2ncccc2Oc2cccnc12	0.11	0.565	1
WAXBOO	C11 H14 N2 O1	P21/c	14	C1CN2CCOc3ccccc3C2N1	0.00	0.454	0
WECCEO	C8 H10 N2 O2	P21/c	14	O=C1C(=O)C2=C1NCCCCN2	0.00	0.217	0
WEHYAK	C4 H5 N1 O3	P21/n	14	O=C1COC(=O)CN1	0.10	0.189	1

Table S3 (continued)

CSD Refcode	Formula	Space Group symbol	Space Group number	SMILES	Energy rank ΔE(CSP) / kJ/mol	$RMSD_{30}$ of CSP match / Å	Numerical rank
WEMWAN	C11 H16 O2	P41212	92	O=C1CCCC2CC1CCCC2=O	0.00	0.293	0
WEPZOJ	C9 H6 N4 O2	P21/n	14	O=C1NNC(=O)c2cc3N=CNc3cc12	0.00	0.199	0
WEXREY	C6 H5 N3	Pna21	33	N1C=Nc2ncccc12	0.00	0.281	0
WIGBOE	C10 H18 N4	P21/n	14	C1CN2CCN3CCN4CCN1C2C34	0.00	0.222	0
WIGYIV	C8 H6 N2 O1	P21/c	14	O=C1NN=Cc2ccccc12	1.17	0.235	1
WIKCAU	C5 H4 O2	Pna21	33	O=C1OC=CC=C1	2.41	0.473	35
WIPVEW	C12 H12 O2	P21212	18	O=C1C2=C1CCCC1=C(CCC2)C1=O	0.14	0.777	3
WIXYUY	C12 H14 O2	P21/n	14	C1CC2(C1)COc1ccccc1OC2	0.00	0.186	0
WOCGOK	C8 H4 F1 N1 O2	P21/c	14	Fc1ccc2NC(=O)C(=O)c2c1	0.00	0.273	0
WOLGOV	C10 H9 N1 O4	P212121	19	O=C1CC2C3C(C1CC2=O)C(=O)NC3=O	1.42	0.478	4
WUDJOU	C14 H16 O1	P21/c	14	O=C1C2(CC2)C2(CC2)C21C1(CC1)C12CC1	0.20	0.379	1
WUDJUA	C10 H12 O2	P-1	2	O=C1CCC2(O1)C1(CC1)C12CC1	1.12	0.923	3
WUTSEJ	C7 H8 O2	P21/c	14	O=C1CC2OC(C1)C=C2	2.21	0.121	8
XAMRUY	C11 H14 N2 O2	P21/n	14	O=C1OCCCN2C=C3CC1CCC3=N2	0.00	0.170	0
XAMZIV	C10 H10 O3	P212121	19	O1C2C1C1C3C4OC4C2C2OC2C13	0.00	0.247	0
XAYMIT	C10 H6 N4	P212121	19	c1cnc2c(ccc3nccnc23)n1	0.00	0.164	0
XDHURC	C3 H4 N2 O3	P212121	19	O=C1CONC(=O)N1	2.47	0.192	14
XENSAL	C9 H10 O2	P212121	19	O=C1C2CC3COC(C=C2)C13	0.00	0.233	0
XERBEB	C3 H3 N3 O2	Pbca	61	O=C1NC=NC(=O)N1	0.00	0.369	0
XESTUM	C13 H10 N2	P21/c	14	C1CC21c1cccnc1c1ncccc21	0.00	0.186	0
XEZHAL	C12 H10 O2	Pbca	61	O=C1C2C=CC(C3C2C2C4C3C24)C1=O	0.00	0.111	0
XIHYOD	C13 H12 O2	P21/c	14	O=C1C=CC2C3C=CC4(CCCC124)C3=O	0.00	0.251	0
XIWDOW	C8 H14 N2 O4	P21	4	O=C1COCC(=O)NCCOCCN1	0.00	0.233	0
XOFTER	C8 H12 N2 O2	C2/c	15	O=C1CCC2NC(=O)CCC2N1	1.66	0.537	7
XOLQOF	C10 H6 N2 O2	P21/n	14	O=C1N2C=CC=C2C(=O)N2C=CC=C12	0.00	0.259	0
XOQLOF	C9 H4 F3 N1	P21/n	14	Fc1cc(F)c2ncccc2c1F	0.00	0.117	0
XOQLUL	C9 H5 F2 N1	Pc	7	Fc1cc(F)c2cccnc2c1	0.04	0.698	1
XUGHUD	C8 H6 N2 O1	P-1	2	[NH2+]=C1[N-]C(=O)c2ccccc12	0.00	1.745	0
XUGHUD01	C8 H6 N2 O1	P21/n	14	N=C1NC(=O)c2ccccc12	None	None	None

Table S3 (continued)

CSD Refcode	Formula	Space Group symbol	Space Group number	SMILES	Energy rank ΔE(CSP) / kJ/mol	$RMSD_{30}$ of CSP match / Å	Numerical rank
XULDUD	C6 H6 O1	Pbca	61	C1CC2=COC=C12	0.27	0.170	3
XURWIS	C10 H12 O3	P21/n	14	O=C1CC2CCCC3OC(=O)C1C23	0.00	0.377	0
XUZXUM	C8 H8 O2	P212121	19	O=C1CC2C3OC(C=C3)C12	1.02	0.199	2
XZBTZO	C8 H4 N4 O2	Pbca	61	[O-]C1=C2N=Nc3ccccc3[N+]2=NO1	2.07	0.199	1
YAPMEI	C8 H10	Pna21	33	C=C1C2(CC2)C21CC2	0.00	0.182	0
YAXQAS	C12 H9 N1 O2	P21/n	14	O=C1NC2=C(CCc3ccccc23)C1=O	3.23	0.340	5
YAZKOZ	C10 H18 N2 O2	P21/c	14	O=C1CCCCC(=O)NCCCCN1	1.81	0.779	6
YEHHEY	C10 H14 O3	P-1	2	O=C1CCCCC2C(COC2=O)C1	0.00	0.242	0
YERJIO	C6 H8 N2 O2	Pccn	56	O=C1NC2CCC1NC2=O	0.14	0.177	1
YERJOU	C6 H8 N2 O2	P21212	18	O=C1NC2CCC1NC2=O	0.00	0.114	0
YETRIY	C12 H12 O2	C2/c	15	O=C1OC2CC34CC=CCC3(C=C2)C14	2.17	0.230	8
YIBCOD	C11 H11 N1 O1	P21/n	14	O=C1C2=CC=CN2C2C3CCCC123	0.26	0.182	1
YIGTEO	C8 H6 O4	P21/n	14	C1Oc2cc3OCOc3cc2O1	1.25	1.658	7
YIMNEO	C12 H12 O3	P21/c	14	O=C1CCC23OC4(CCC(=O)C4C12)C=C3	0.00	0.170	0
YIRFUA	C12 H12 O1	P212121	19	O=C1CCC2CC2c2ccccc12	0.00	0.102	0
YITWOQ	C9 H6 O3	P212121	19	O=C1COC(=O)c2ccccc12	0.00	0.216	0
YOBQAH	C4 H5 N1 O2	P21/c	14	C=C1CNC(=O)O1	1.76	0.243	4
YOKZAB	C11 H9 N1 O3	P21/c	14	O=C1CCC2OC(=O)c3ccccc3N12	0.00	0.252	0
YONFAK	C13 H11 N1 O1	P21/c	14	O=C1CCCc2nc3ccccc3cc12	0.00	0.177	0
YOPXIL	C8 H11 N1 O1	P21	4	O=C1NCC2C=CCCC12	0.00	0.188	0
YOWLEC01	C11 H14 N4	Pbca	61	C1N2CN3CN1CN(C2)c1ccccc31	0.00	0.360	0
YOZKUT	C9 H12 O2	P41212	92	O=C1CC2CC(C1)CC(=O)C2	2.16	0.102	5
YULDIU	C12 H16 O2	P21/n	14	O=C1CC23CCCCC2(C1)CC(=O)C3	0.00	0.155	0
YUQWOX	C11 H7 N3	P21/c	14	N=C1c2cccnc2c2ncccc12	15.05	1.127	148
YUXLEI	C13 H9 N1 O1	Pna21	33	O1c2ccccc2C=Nc2ccccc12	0.39	0.212	2
YUYFAZ	C9 H13 N1 O3	Pbca	61	O=C1OCC2ON3CCCCC3C12	0.12	0.222	1
YUYFED	C10 H15 N1 O3	P212121	19	O=C1OCCC2ON3CCCCC3C12	0.00	0.172	0
YUYLUZ	C10 H8 O4	P21/c	14	O=C1C2OC=CC1C1OC=CC2C1=O	4.98	0.170	3
ZASGUY	C4 H4 N2 O2	P21/c	14	O=C1NC=CNC1=O	0.66	0.313	2

Table S3 (continued)

CSD Refcode	Formula	Space Group symbol	Space Group number	SMILES	Energy rank ΔE(CSP) / kJ/mol	$RMSD_{30}$ of CSP match / Å	Numerical rank
ZEFXIR01	C7 H6 N2 O1	P21/n	14	O=C1Nc2ccccc2N1	0.02	0.317	1
ZELDOJ	C6 H2 F4	P21/n	14	Fc1ccc(F)c(F)c1F	0.86	0.293	8
ZELDOJ01	C6 H2 F4	C2/c	15	Fc1ccc(F)c(F)c1F	0.34	2.168	1
ZETGOX	C9 H12 O4	P212121	19	O=C1OC2COCOCC3CC2C13	0.00	0.146	0
ZETGUD	C10 H12 O4	P212121	19	O=C1CCC2CC3C2C(CO1)OC3=O	0.00	0.238	0
ZOBXIX	C11 H13 N1 O1	P-1	2	O=C1NC2C3CC4C5CC(C2C35)C14	0.26	0.262	1
ZOBXOD	C11 H13 N1 O1	P21/c	14	O=C1NC2C3CC4C5C3CC2C5C14	0.00	0.114	0
ZOFPAL	C3 F4	Pbcn	60	FC1=C(F)C1(F)F	0.76	0.221	7
ZOKQIC	C11 H13 N1 O3	P21	4	O=C1CCC2CC=CCC32COC(=O)N13	1.37	1.471	3
ZORQIG	C15 H14	P21/c	14	C1C2C3C4C1C1C2C2(C=C2)C3C4C21C=C2	0.00	0.205	0
ZOTCTD10	C11 H14 N2 O2	C2/c	15	O=C1CC2CCC3CC(=O)N4CCN1C234	0.00	0.169	0
ZOYLII13	C8 H7 N1 O1	P21/c	14	O=C1Cc2ccccc2N1	3.86	0.232	19
ZTCDON10	C9 H13 N1 O1	P21/c	14	O=C1NC2CC3CCC2C1C3	3.63	0.304	13
ZTNONX	C7 H8 N2 O2	P21/n	14	[O]N1=N([O])C2C3CC4C(C24)C13	0.00	0.152	0
ZUFJER	C8 H2 F4 N2	P21/n	14	Fc1c(F)c(F)c2nccnc2c1F	1.38	0.276	2
ZUFJIV	C8 H3 F3 N2	Pca21	29	Fc1cc(F)c2nccnc2c1F	1.50	0.232	4
ZUMKUN	C13 H16 O2	P21/c	14	O=C1C2CCC31CCCCC13OC2C=C1	2.32	0.158	5
ZUNTAD	C9 H8 O4	P212121	19	O=C1C2CCCC(C1=O)C(=O)C2=O	0.00	0.201	0
ZUTKEG	C4 H3 N5 O1	Pnma	62	O=C1NC=CC2=NN=NN12	0.00	0.240	0
ZZZJIE01	C10 H6 O2	Pc	7	O=C1C=Cc2ccccc2C1=O	2.40	0.254	1
ZZZKEA01	C6 H4 N2 O2	P-1	2	[O-][N+]1=C2C=CC=CC2=NO1	0.00	0.196	0
ZZZRNY02	C11 H8 N2	P212121	19	N1c2ccccc2c2ccncc12	3.39	0.250	20
ZZZSAE01	C8 H8	Aba2	41	C1=CC=CC=CC=C1	0.00	0.119	0
ZZZVYE01	C5 H10	P21/c	14	C1CCCC1	0.43	0.834	3
ZZZWEO02	C4 H8	I2/m	12	C1CCC1	1.20	0.766	6
ZZZWGK01	C6 H10 O1	P212121	19	O=C1CCCCC1	0.26	0.125	4
ZZZWGK04	C6 H10 O1	P21/n	14	O=C1CCCCC1	1.79	0.183	26
